# Toward Near-Infrared Emission in Pt(II)-Cyclometallated Compounds: From Excimers’ Formation to Aggregation-Induced Emission

**DOI:** 10.1021/acs.inorgchem.2c03490

**Published:** 2023-01-25

**Authors:** Ariadna Lázaro, Ramon Bosque, Jas S. Ward, Kari Rissanen, Margarita Crespo, Laura Rodríguez

**Affiliations:** † Departament de Química Inorgànica i Orgànica, Secció de Química Inorgànica, Universitat de Barcelona, Martí i Franquès 1-11, E-08028 Barcelona, Spain; ‡ Institut de Nanociència i Nanotecnologia (IN2UB), Universitat de Barcelona, 08028 Barcelona, Spain; § Department of Chemistry, University of Jyvaskyla, P.O. Box 35, 40014 Jyväskylä, Finland; ∥ Institut de Biomedicina de la Universitat de Barcelona (IBUB), 08028 Barcelona, Spain

## Abstract

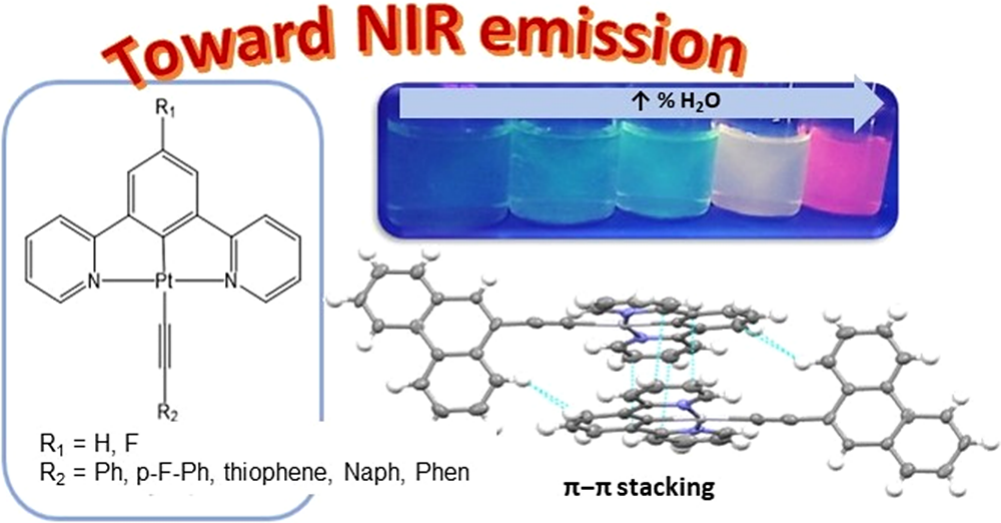

Two series of Pt(II)-cyclometallated compounds containing N^C^N tridentate and alkynyl-chromophore ligands have been synthesized and structurally characterized. The N^C^N ligands differ on the presence of R_1_ = H or F in the central aromatic ring, while six different chromophores have been introduced to the alkynyl moiety. Single-crystal X-ray structures for some of the compounds reveal the presence of weak intermolecular contacts responsible for the formation of some dimers or aggregates. The photophysical characterization shows the presence of two emission bands in solution assigned to the ^3^π–π* transition from the N^C^N ligands mixed with ^3^MLCT/^3^ILCT transitions (higher energy band) in deaerated samples. The formation of excimers has also been identified as a broad band at longer wavelengths [near-infrared (NIR) emission] that becomes the main emission band for compounds containing phenanthrene as the chromophore. NIR emission behavior has also been explored using acetonitrile/water mixtures, and the presence of aggregates that emit at ca. 650 nm has also been detected.

## Abstract 2

Two series of Pt(II) complexes have been synthesized and their photophysical data carefully analyzed in solution and powder. Aggregation studies were performed in acetonitrile/water mixtures. Infrared emitters were obtained through excimers’ formation or aggregation.

## Introduction

Luminescent transition metal complexes display several applications in a variety of fields such as device fabrication, molecular probes, sensors, or organic light-emitting diodes (OLEDs) among others, and the research in this field has rapidly increased in the last few years due to their intrinsic photophysical properties.^1−3^ They have been observed to display in some cases better properties in comparison to organic fluorophores, such as enhanced photostability (that allows continuous exposure of the complexes to irradiation), long luminescence lifetimes (from hundreds of nanoseconds to microseconds or even milliseconds) that allow the elimination of interference from the autofluorescence background, and the possibility to tune room temperature phosphorescence.

The d^8^ platinum(II) complexes are particularly relevant since they present unique supramolecular self-assembly properties that are not observable in the octahedral d^6^ and tetrahedral d^10^ metal complexes. They present square-planar geometries that can undergo face-to-face intermolecular interactions through ligand–ligand and Pt(II)···Pt(II) interactions, which can give rise to new electronic excited states that produce red-shifted emission from triplet metal–metal-to-ligand charge-transfer (^3^MMLCT) or excimeric ^3^IL excited states in addition to the one arising from the mononuclear Pt(II) moiety.^1,4−12^ These assemblies are of great relevance to modulate the resulting photophysical properties, both regarding their energy and emission intensity by subtle changes to their environment. It should be taken into consideration that aggregation can happen in the ground state (dimers) or the excited state (excimers). Additionally, Pt(II), as a heavy atom, induces strong spin–orbit coupling, favoring the population of the T1 triplet excited state by enhanced intersystem crossing from S1 → T1 and producing phosphorescence at room temperature.^4,13^

The use of cyclometallating ligands is a convenient strategy to favor luminescence since the strong field of these ligands tends to favor emission efficiencies as they raise the energies of deactivating metal-centered states, reducing non-radiative deactivation pathways. Tridentate cyclometallated ligands are particularly relevant since they have been observed to induce higher rigidity on the complex with respect to bidentate ligands, inhibiting distortion and reducing non-radiative deactivation processes.^3^

The nature of the cyclometallated ligands and co-ligands and the ionic or neutral character of the molecules are extremely relevant to modulate the absorption and emission properties. Thus, the study of Pt(II)-cyclometallated compounds is currently a highly relevant research field in order to achieve high luminescence (mainly from the triplet excited state, i.e., phosphorescence) quantum yields, color tunability, and stability.^7,14,15^ In particular, C^N^N and N^C^N coordination modes are more commonly found in tridentate cyclometallating ligands, and they have been observed to exhibit intense luminescence and versatile emissive excited states, including not only intraligand (IL) (π–π*) excited states but also the excimeric excited states.^16−18^ Among them, N^C^N coordination seems to favor higher emission intensities and quantum yields.^9^ Fewer efforts have been made on the analysis of the co-ligand that occupies the fourth coordination position at the Pt(II) center, even though they can also have an influence on their resulting assemblies and luminescent properties.^19^

An interesting tangent in the field is the modulation of the chemical structures and assemblies to shift the emission to the red since, for example, OLEDs and materials that emit in the infrared (IR) or near-infrared (NIR) region represent a challenging target due to the favored deactivation processes in low-energy emissive populated states.^20^ IR and NIR emission is of vital importance in several relevant applications such as full-color displays, remote sensing of environmental conditions, night-vision displays, bio-chemosensors, in vivo imaging, light-fidelity (Li-Fi) communication, or security authentication devices, and it is mostly explored with pure organic molecules.^21−25^ Different strategies can be followed to modulate the final emission of the complexes to red, with excimers’ formation and aggregation-induced emission (AIE) being popular design methodologies.^10,20,22,26−30^

In this work, we have designed and synthesized two series of Pt(II)-cyclometallated complexes containing tridentate N^C^N ligands with an alkynyl chromophore as the co-ligand occupying the fourth coordination position. The different chromophores have been chosen in order to evaluate how the electron-withdrawing character (fluorine), soft atom (sulfur in thiophene), or extended aromaticity (benzene, naphthalene, and phenanthrene) can affect the resulting packing through intermolecular contacts affecting their luminescence. Additionally, the two series of compounds differ on the presence of a H or F atom at the central benzyl ring, which could also confer different intermolecular forces in the packing. The differences in the resulting photophysical properties depending on the N^C^N ligand and co-ligands have been analyzed in detail together with the resulting AIE processes, with those being observed to shift the emissions up to ca. 700 nm, thanks to excimer and aggregate formation.

## Results and Discussion

### Synthesis and Characterization

**Scheme 1:**
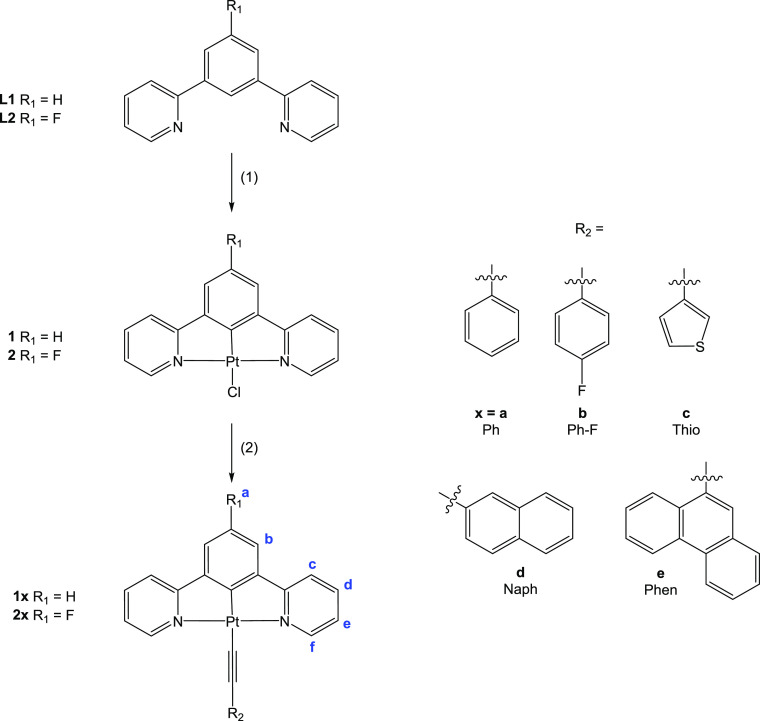
Synthesis of Cyclometallated Platinum(II) Compounds (1) K_2_PtCl_4_, acetic acid/water 9:1, microwave heating, 160 °C, 30 min. (2) R_2_-C≡C-H, NaOH, methanol, r.t., 24 h. The labeling convention used for NMR results is shown for the new compounds.

All compounds were synthesized by following the route summarized in Scheme 1. As reported in the literature, the corresponding precursors **1** and **2** were reacted with different alkynyl aromatic ligands with sodium hydroxide as a base.^31^ Final compounds **1x** and **2x** were obtained as orange solids with moderate yields (54–80%) after precipitation and washing with water, methanol, and hexane.

Characterization of the synthesized compounds by ^1^H NMR spectroscopy showed the disappearance of the terminal alkynyl proton (R_2_-C≡C-***H***) from the corresponding alkynyl aromatic ligand. This observation together with the appearance of the aromatic protons of the new ancillary ligand confirms the correct substitution of the chlorido and formation of the desired platinum complexes. A significant downfield shift in the pyridine proton (H^f^) of ca. 0.10–0.25 ppm is observed when compared with the precursors (9.36–9.38 ppm). However, its coupling constant with the platinum atom is not affected since the ligand *trans* to the nitrogen of the pyridine bond is not exchanged (Figures S1–S9). For family **2x**, all compounds were also characterized by ^19^F NMR spectroscopy, observing only one signal as a triplet due to the coupling with the two adjacent aromatic protons of the central ring. A second signal is present in the NMR spectrum of compound **2b** due to the aromatic *p*-fluorobenzene moiety (Figures S10–S15).

The C≡C (around 2070 cm^–1^) vibration as well as the disappearance of the band assigned to the stretching of the terminal proton of the free alkynyl moiety (around 3300 cm^–1^) was determined by IR spectroscopy. Further confirmation of the successful formation of the products was checked by electrospray ionization [ESI(+)] mass spectrometry finding the protonated molecular peak for all cases (Figures S16–S24).

**Figure 1:**
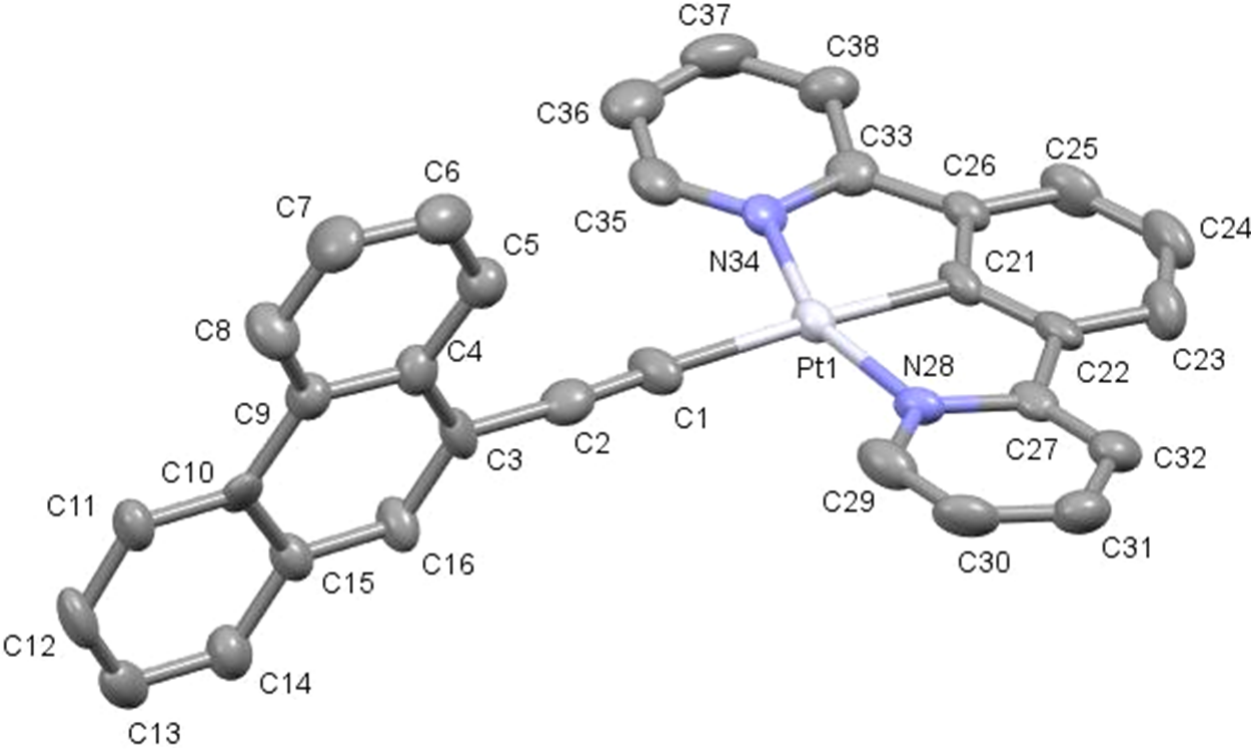
Molecular structure of compound **1e**. Selected bond lengths (Å) and angles (°) with estimated standard deviations: Pt(1)–N(34): 2.038(7); Pt(1)–C(21): 1.939(7); Pt(1)–N(28): 2.035(7); Pt(1)–C(1): 2.077(8); C(1)–C(2): 1.181(11); N(34)–Pt(1)–C(21): 79.4(3); C(21)–Pt(1)–N(28): 79.7(3); N(28)–Pt(1)–C(1): 100.2(3); C(1)–Pt(1)–N(34): 100.9(3); Pt(1)–C(1)–C(2): 177.71(9). The thermal ellipsoids are drawn at the 50% probability level, and hydrogen atoms are omitted for clarity.

**Figure 2:**
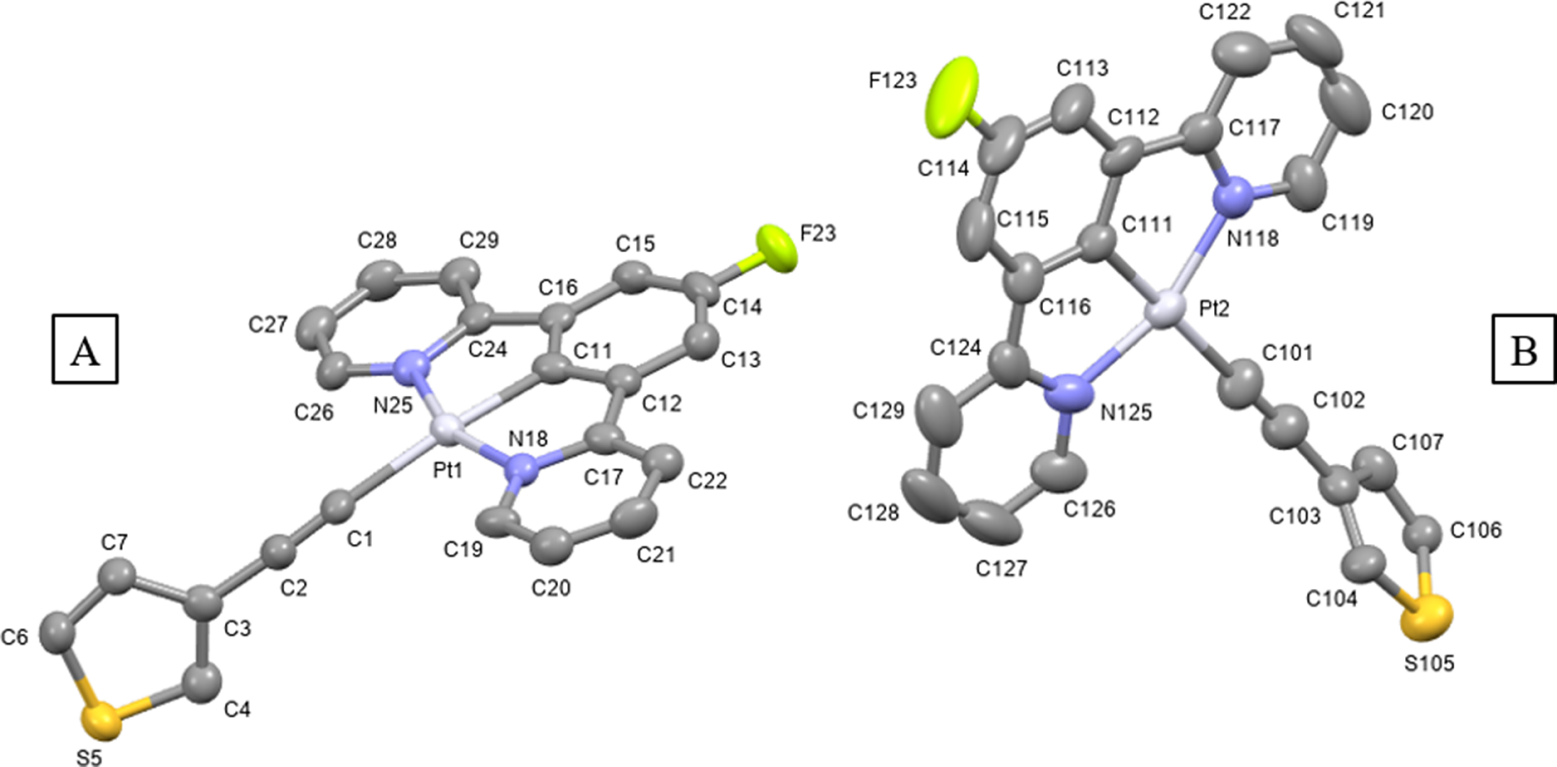
Molecular structure of compound **2c** (two crystallographically independent molecules present in the asymmetric unit cell, A and B). Selected bond lengths (Å) and angles (°) with estimated standard deviations: (A) Pt(1)–N(18): 2.033(5); Pt(1)–C(11): 1.942(7); Pt(1)–N(25): 2.035(5); Pt(1)–C(1): 2.099(7); C(1)–C(2): 1.179(9); N(18)–Pt(1)–C(11): 80.0(3); C(11)–Pt(1)–N(25): 80.1(3); N(25)–Pt(1)–C(1): 98.0(2); C(1)–Pt(1)–N(18): 101.7(2); Pt(1)–C(1)–C(2): 165.2(6). (B) Pt(2)–N(118): 2.040(5); Pt(2)–C(111): 1.930(8); Pt(2)–N(125): 2.038(7); Pt(2)–C(101): 2.054(8); C(101)–C(102): 1.185(10); N(118)–Pt(2)–C(111): 80.1(3); C(11)–Pt(2)–N(125): 79.3(3); N(125)–Pt(2)–C(101): 99.9(3); C(101)–Pt(2)–N(118): 100.8(3); Pt(2)–C(101)–C(102): 178.7(6). The thermal ellipsoids are drawn at the 50% probability level, and hydrogen atoms are omitted for clarity.

Single crystals suitable for X-ray diffraction analysis were grown for compounds **1e** and **2c** (Table S1) by slow diffusion of methanol or hexane, respectively, into a concentrated dichloromethane solution of the compound. Compound **1e** presents one single molecule in the asymmetric unit, while for compound **2c**, two crystallographically independent molecules are observed (Figures 1 and 2). As shown in Figures S25 and S26, the unit cell of both compounds contains four molecules. The platinum atom adopts the expected square-planar environment completed by the tridentate [N^C^N] ligand and the alkynyl group. Bond distances and angles are in agreement with those reported in the literature for analogous [N^C^N] platinum(II)-cyclometallated compounds.^32,33^ The aromatic ring attached to the alkynyl moiety is almost perpendicular to the cyclometallated unit for compound **1e** (85.3°), while for the two molecules in the asymmetric unit of compound **2c**, they are observed in two different conformations with angles of 52.1° and 83.3°.

**Figure 3:**
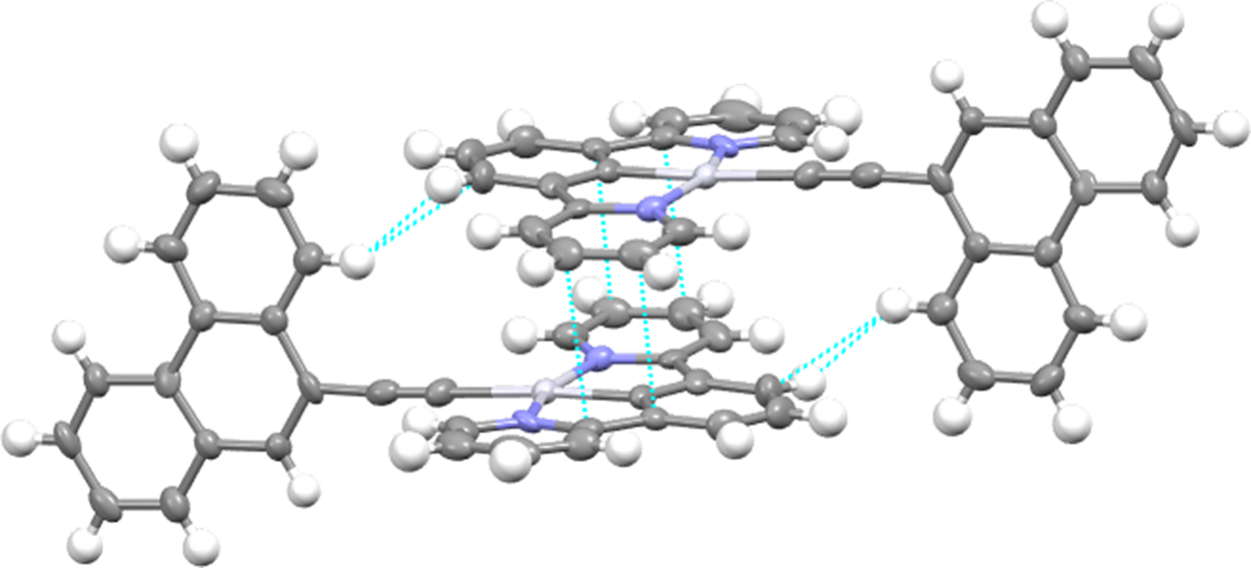
View of the relevant intermolecular short contacts for compound **1e** highlighted in blue: π···π: 3.361 and 3.345 Å; C···H: 2.849 Å. Gray, platinum; blue, nitrogen.

**Figure 4:**
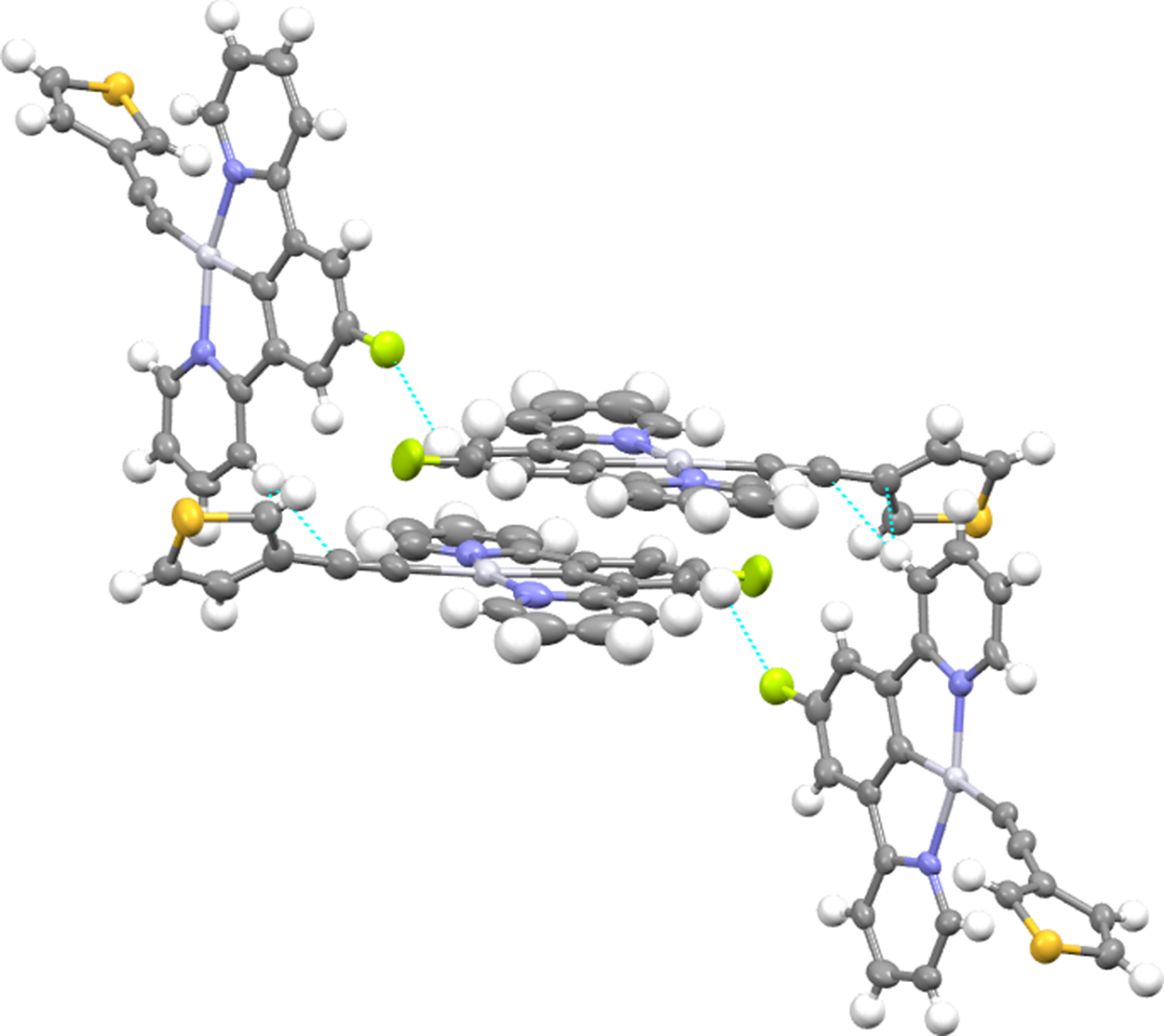
View of the relevant intermolecular short contacts for compound **2c** highlighted in blue: F···H: 2.449 Å; C···H: 2.870 Å. Gray, platinum; blue, nitrogen; yellow-green, fluorine; orange, sulfur.

The packing for both compounds presents a zig-zag conformation held together by the stacking of two cyclometallated moieties (Figures S25 and S26). These dimeric structures are arranged in a head-to-tail fashion, which does not permit the presence of metallophilic interactions in **1e** (with a Pt···Pt distance of 5.0521(5) Å). A similar distance was observed for Pt2···Pt2 of 4.9888(4) Å in **2c**. However, in **2c**, a much shorter Pt···Pt distance of 3.4654(4) Å was observed for Pt1···Pt1, which is just below the combined van der Waals radii (3.5 Å) for this interaction.^31^ As depicted in Figure 3, for compound **1e**, these dimer-like structures are due to the presence of π–π stacking (3.36 and 3.35 Å) and C···H (2.85 Å) intermolecular short contacts, which results in an interplanar distance of 3.0 Å. Additional C–H···π intermolecular contacts of 3.03 Å between the phenanthrene groups of two adjacent molecules can be identified, which could be responsible for different emissive properties (see below). However, for compound **2c**, we can only observe F···H: 2.45 Å and C···H: 2.87 Å interactions, leading to a higher interplanar distance of 3.2 Å (Figure 4).

### Photophysical Characterization

The absorption spectra of all compounds (final products **1a–e** and **2a–e** and precursors) were recorded in 5 × 10^–5^ M dichloromethane solutions at 298 K, and the results are shown in Figures S27–S29 and Table 1. In the higher energy region (270–350 nm), all compounds present intense bands that can be attributed to ^1^π–π* IL transitions of the cyclometallated 1,3-di(2-pyridyl)benzene, which are also recorded in the absorption of the free ligands **L1** and **L2** (Figure S30), overlapped, in some cases, with ^1^π–π* IL transitions of the alkynyl aromatic moiety. In the lower energy region (350–450 nm), less intense bands are observed, which can correspond to mixed charge-transfer and ligand-centered character transitions, according to the literature.^7−9,12,16,26,28,34−36^

**Table 1:** Electronic Absorption and Emission Data Including Absorption and Phosphorescence Emission Maxima and Phosphorescence Quantum Yields Recorded in Air-Equilibrated (with O_2_) or Degassed (N_2_ sat.) Dichloromethane Solutions at Room Temperature

compound	λ_abs_, nm (ε × 10^–3^, M^–1^ cm^–1^)	λ_em_ (nm)	ϕ_Ph_ (with O_2_)	ϕ_Ph_^0^ (N_2_ sat.)	ϕ_Ph_^0^ monomer (N_2_ sat.)	ϕ_Ph_^0^ excimer (N_2_ sat.)
**L1**	280 (8.6)	341	0.004	0.004	0.004	
**L2**	279 (8.9)	337	0.007	0.007	0.007	
**1**	290 (18.4), 333 (5.8), 380 (7.2), 402 (5.9)	490	0.034	0.605	0.605	
**2**	290 (12.5), 378 (4.2), 422 (4.7)	505	0.030	0.660	0.660	
**1a**	291 (17.0), 389 (4.6)	497, 702	0.039	0.605	0.456	0.149
**1b**	290 (18.2), 388 (5.5)	495, 692	0.045	0.558	0.390	0.168
**1c**	292 (25.5), 391 (7.0)	501, 698	0.032	0.518	0.357	0.161
**1d**	288 (8.7), 309 (5.8), 389 (2.3)	490, 693	0.041	0.547	0.447	0.100
**1e**	289 (28.4), 319 (22.3), 334 (24.0), 391 (7.7)	547, 698	0.012	0.342	0.130	0.212
**2a**	289 (10.1), 380 (3.5), 421 (3.5)	507, 692	0.040	0.596	0.485	0.111
**2b**	292 (20.6), 390 (5.5), 422 (5.7)	511, 694	0.028	0.491	0.340	0.151
**2c**	291 (10.8), 391 (2.7), 424 (2.8)	513, 696	0.033	0.587	0.455	0.132
**2d**	294 (31.5), 310 (2.5), 392 (6.8), 427 (7.5)	520, 699	0.027	0.506	0.274	0.232
**2e**	290 (32.1), 319 (25.6), 333 (26.6), 425 (8.2)	547, 700	0.011	0.373	0.069	0.304

**Figure 5:**
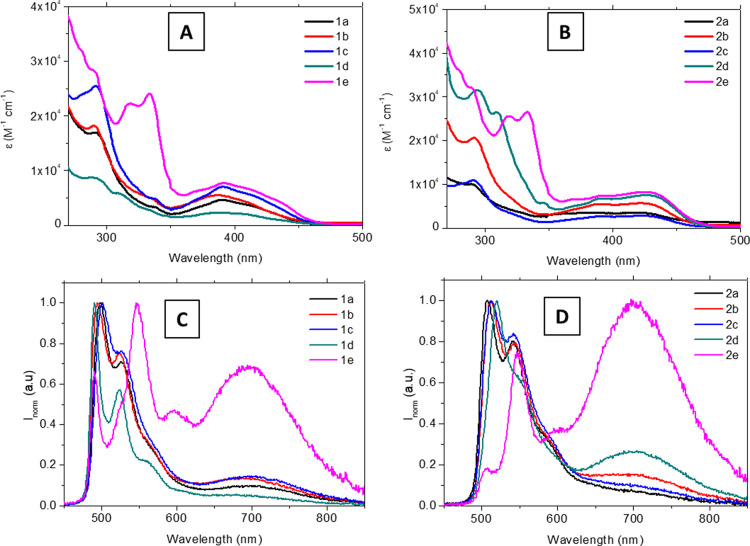
Absorption spectra (A for series **1** and B for series **2**) and normalized emission spectra (C for series **1** and D for series **2**) for N_2_-saturated 5 × 10^–5^ M dichloromethane solutions of the compounds at 298 K (λ_exc_ = 390 nm).

The samples were excited at their lower energy absorption band, and the recorded emission spectra display a vibronically structured band centered around 500 nm (Figure 5) that can be attributed to a primarily ligand centered ^3^π–π* transition from the N^C^N ligands that can be mixed with ^3^MLCT/^3^ILCT transitions, involving the cyclometallated ligand. The same pattern has been recorded in the emission of the chlorido precursors (Figure S31).^3,4,8,12,16,36^ The large Stokes shift and the quenching of the emission in the presence of oxygen support their phosphorescent emission assignment (Table 1). The free **L1** and **L2** ligands present a broad emission band around 350 nm, which can be assigned to a fluorescent ^1^π–π* IL transition that is not quenched by the presence of oxygen (Figure S32), proving that the phosphorescent emission of these compounds is due to the perturbation from the platinum atom. Another band centered at 700 nm is observed for all final platinum complexes **1a–e** and **2a–e**, which can be assigned to excimer formation.^7,37^ The excimer assignment can be done, thanks to the excitation spectra that resemble the same pattern (that fits the absorption spectra) when the emission is collected at the two maxima (500 and 700 nm, see Figure S33). The lack of excimer formation recorded for **1** and **2** together with the different excimer intensities recorded in the different cases indicates the main role of the alkynyl-R_2_ chromophore in the excimer formation. This excimer is observed to be more favored for series **2** complexes and mainly for the more extended chromophore, phenanthrene, in compound **2e**. In fact, excimer emission has been previously reported in the literature for other LPt-Cl analogues to **1** and **2** only at higher concentrations.^38^ The total phosphorescence quantum yields (QY) increase in deaerated solutions, reaching values up to 60%, similar to the values reported for analogous compounds.^28,39^ Global QY values have been split into their monomer and excimer contribution (Table 1), and it can be observed that, in general, the monomer emission is the major radiative deactivation pathway. Instead, the excimer has a larger contribution only for the phenanthrene derivatives **1e** and **2e**, with almost a total dominance in compound **2e** (Table 1 and Figure 5). This could be ascribed to the establishment of π–π intermolecular contacts in solution between the phenanthrene groups that have been identified to be very close to each other in the three-dimensional X-ray crystal packing in the solid state of **1e** (Figure S25). Thus, although no significant differences have been observed in the global QY values of the platinum complexes when the two series of compounds are compared, some significant differences can be observed when the emission efficiency is split between the two contributions (monomer and excimer). In this case, the principal parameters that seem to have the main effect are the extended aromaticity (a larger contribution of the excimer for **1e** and **2e**) and the H or F atom at the central N^C^N tridentate ligand, with a larger effect in the presence of a fluorine in the excimer formation. This may be due to the resulting less electronic density in the phenanthrene aromatic ring, making them more suitable for establishing intermolecular contacts.

Phosphorescence emission lifetimes were recorded for both the monomer and the excimer emission transitions and are of hundreds of nanoseconds. These values increase to a few microseconds in deaerated solutions, and they are in the range of those reported previously in the literature for similar compounds (Table 2) and support triplet emission origin.^4,28,40,41^ The recorded values for the final platinum complexes are, in general, in the same order as those recorded for the platinum precursors **1** and **2**. Phenanthrene derivatives **1e** and **2e** are again a particular case that displays longer decay time values than the rest of the compounds as previously observed for other platinum cyclometallated compounds containing this chromophore.^4^ Time-resolved phosphorescence spectra were studied for these two compounds (**1e** and **2e**), and the resulting kinetics show the clear predominance of the monomer emission with respect to the excimer at increasing time-gating in agreement with the longer decay times recorded (see Figure S34).

**Table 2:** Phosphorescence Lifetimes of the Compounds Recorded in Air-Equilibrated (with O_2_) or Degassed (N_2_ sat.) Dichloromethane Solutions

compound	τ (μs) monomer (with O_2_)	τ (μs) excimer (with O_2_)	τ° (μs) monomer (N_2_ sat.)	τ° (μs) excimer (N_2_ sat.)
**L1**	3.91 ×10^–3^			
**L2**	2.26 ×10^–3^			
**1**	0.42		8.33	
**2**	0.39		6.90	
**1a**	0.32	0.16	5.25	2.94
**1b**	0.44	0.20	7.35	3.28
**1c**	0.22	0.11	4.55	2.99
**1d**	0.22	0.14	4.91	2.94
**1e**	0.38	0.29	41.39	5.27
**2a**	0.54	0.22	5.87	3.12
**2b**	0.42	0.19	6.96	3.00
**2c**	0.37	0.16	5.37	2.66
**2d**	0.45	0.16	7.24	3.68
**2e**	0.560	0.34	34.44	6.88

Radiative and non-radiative rate constants have been calculated in all cases from the experimental quantum yields and lifetime values (Table S2). According to these values, the increase in the recorded quantum yields from the monomer emission band in deaerated samples can be ascribed mainly to a significant decrease of the non-radiative deactivation pathway rate. We can observe that *k*_nr_ values are one order of magnitude smaller than those in air-equilibrated samples, while *k*_r_ values stay similar. Additionally, looking at the excimer emission band, we can observe that the *k*_nr_ values for phenanthrene derivatives **1e** and **2e** are smaller than those calculated for the other platinum complexes, in agreement with the significant increase in the emission intensity of this band.

In the solid state (powder), all compounds present a broad emission band centered between 579 and 626 nm (Figure S35 and S36). This emission band can be assigned to the emission of the π–π stacked aggregated forms, which is in accordance with the observation of the dimer formation in the obtained crystalline structures.^31^ The phosphorescence quantum yields have moderate values up to 12%, and the corresponding lifetimes are of hundreds of nanoseconds (Table 3), in agreement with triplet state origin emission. It can be observed that the quantum yields of **series 1** in the solid state are larger than those in solution, while in the case of **series 2**, these values are in the same order as previously recorded in dichloromethane. This can be rationalized to the more efficient intermolecular packing in the absence of the fluoride atom at the cyclometallated ligand that seems to favor the π–π stacking and the formation of aggregates, as previously observed in the packing in the X-ray diffraction determined structures. Interestingly, the calculated *k*_r_ and *k*_nr_ values show an enhancement of the radiative deactivation channels, with *k*_r_ values being larger in both series **1** and **2** complexes and *k*_nr_ values being smaller than (**1**, **1a–e**) or similar to (**2**, **2a–e**) those previously obtained in air-equilibrated solution samples.

**Table 3:** Electronic Absorption and Emission Data Including Absorption and Emission Maxima and Phosphorescence Quantum Yields Recorded in the Solid State (Powder)

compound	λ_em_^max^ (nm)	ϕ_Ph_	τ (μs)	*k*_r_ (μs^–1^)	*k*_nr_ (μs^–1^)
**L1**	357	0.005			
**L2**	390	0.008			
**1**	559	0.034	0.694	0.049	1.392
**2**	506	0.017	0.489	0.035	2.010
**1a**	612	0.137	0.392	0.349	2.202
**1b**	611	0.092	0.519	0.177	1.750
**1c**	601	0.049	0.593	0.083	1.604
**1d**	610	0.104	0.594	0.175	1.508
**1e**	609	0.119	0.714	0.167	1.234
**2a**	626	0.029	0.403	0.072	2.409
**2b**	617	0.058	0.339	0.171	2.779
**2c**	616	0.039	0.675	0.058	1.424
**2d**	625	0.125	0.419	0.298	2.088
**2e**	623	0.019	0.311	0.061	3.154

### DFT Calculations

#### Geometry Optimizations

Density functional theory (DFT) calculations in solution, using the B3LYP functional, 6-31G*/LANL2DZ basis set, and CPCM solvation model (see the Experimental Section for details), were performed on the systems **1**, **2**, **1a**-**1e**, and **2a**-**2e** in order to rationalize the experimental results. Initially, the molecular geometries of the complexes in dichloromethane were optimized. In all cases, the experimental geometries are well reproduced, with the aromatic group nearly perpendicular to the cyclometallated moiety: torsion angles are 80.5° for **1e** and 75.2° for **2c,** matching well with the experimentally determined values of 85.3 and 83.3, respectively (Figure S39 and Table S3).

We have also studied the rotation of the phenanthrenyl moiety of complex **1e**, using the same level of theory, in order to explore the possibility of the existence of rotational conformers. The calculated barrier of 3.3 kJ/mol is not high enough to preclude the free rotation around the C_alkyne_–C_aromatic_ bond.

We have analyzed the modifications in the platinum environment upon modifying the nature of the ligands. Thus, the substitution of the chlorido ligand by an aryl alkynyl moiety results in an increment of the distance between the platinum and the cyclometallated carbon and a very slight increment of the platinum–nitrogen bond length. The change of a hydrogen atom for a fluorine in the cyclometallated ligand (i.e., going from series **1** to series **2** complexes) results in a shortening of the distance between the platinum and the ligand situated in a *trans* position with respect to the metallated carbon, while the bond length between the metallated carbon and the platinum remains constant, as the one between the platinum and the nitrogen. Finally, changes in the nature of the aromatic ring bonded to the alkyne moiety only result in small variations in the distance between the alkyne carbon and the aromatic carbon bonded to it.

#### UV–Vis Absorption Spectra

TD-DFT calculations were performed on the systems **1**, **2**,**1a**-**1e**, and **2a**-**2e** in solution, using the geometries previously optimized, in order to calculate the ultraviolet (UV) absorption spectra using dichloromethane as a solvent. The most intense transitions are shown in Table S4. Figure S40a,b shows the energy and the nature of the orbitals involved in these transitions for complexes **1** and **1a**-**1e**, as well as the energy of the corresponding transitions. All of these transitions are of π–π* type.

**Figure 6:**
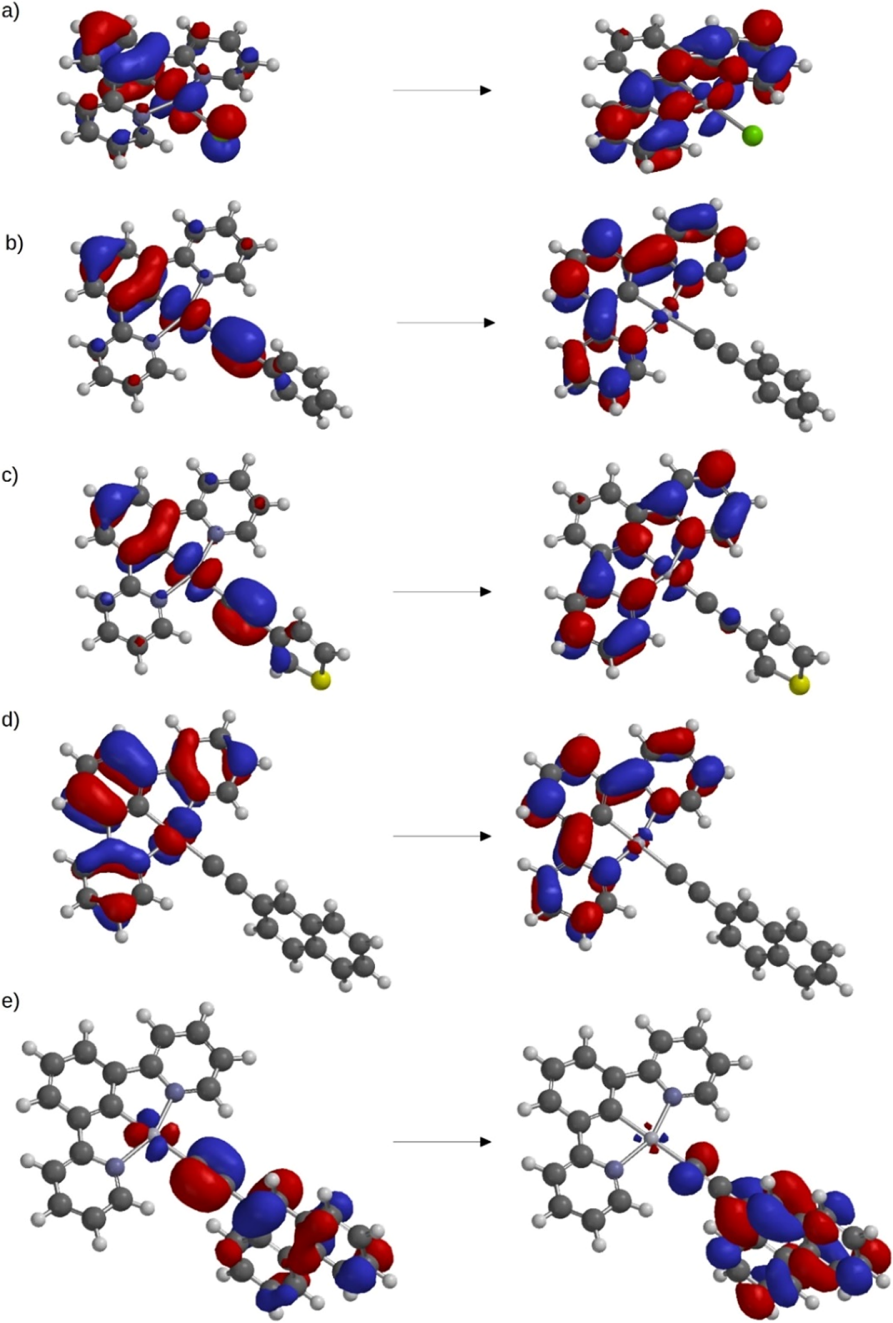
Orbitals that are involved in the transitions described in the text. (a) HOMO and LUMO of complex **1**; (b) HOMO–1 and LUMO+1 of complex **1a**; (c) HOMO–1 and LUMO of complex **1c**; (d) HOMO–3 and LUMO+1 of complex **1d**; and (e) HOMO and LUMO+2 of complex **1e**.

Highest occupied molecular orbital–lowest unoccupied molecular orbital (HOMO–LUMO) transitions are only observable in complexes **1** and **2**. These orbitals, for complex **1**, are shown in Figure 6a; the HOMO orbital is centered mainly in the central ring of the cyclometallated moiety, with smaller contributions from the platinum atom and the chlorido ligand, while the LUMO has the greatest contribution from the N-substituted rings of the same pincer ligand, also with a small contribution of the platinum atom. This transition is ILCT/LLCT in character.

In the remaining systems, the lowest energy absorption corresponds mainly to a HOMO–1 to LUMO+1 transition. The former is based mainly on the alkyne moiety and the central ring of the cyclometallated ligand, with a very small contribution of Pt, while the latter is based mainly on the three rings of the same ligand, as shown in Figure 6b for complex **1a**. Thus, this transition is ILCT/LLCT in character.

Complexes **1a**-**1e** and **2a**-**2e** show an absorption band in the 380–390 nm interval. We have assigned it to a HOMO–1 - > LUMO transition. As previously stated, HOMO–1 has a contribution of the alkyne moiety and the central ring of the cyclometallated ligand, while LUMO is centered on the N-substituted rings of the pincer ligand; thus, this transition is also ILCT/LLCT in character. Figure 6c shows these orbitals for the **1c** complex.

Systems with naphthyl (**1d** and **2d**) and phenanthrenyl (**1e** and **2e**) substituents also feature a band in the 309–319 nm interval. There are several transitions that can contribute to this band, but the one that is always present is HOMO–3→ LUMO+1 (HOMO–3→ LUMO in **2e**). Both orbitals are centered in the pincer ligand, as shown in Figure 6d for the **1d** complex, so this transition can be regarded as ILCT.

Finally, complexes **1e** and **2e** feature a supplementary absorption band at 333–334 nm, which can be ascribed to the HOMO → LUMO+2 transition. Both orbitals are centered mainly in the phenanthrenyl moiety, as shown in Figure 6e for complex **1e**, so this transition can also be regarded as ILCT.

#### Emission Spectra

The triplet excited states for molecules **1**, **2**, **1a**-**1e**, and **2a**-**2e** have been optimized in solution in order to study their emission spectra, and the singlet–triplet transitions have been calculated using dichloromethane as a solvent. The transitions are listed in Table S5. The optimized geometries differ from the ground state in the orientation of the ring attached to the alkyne moiety: while in the ground state, the ring is nearly perpendicular to the cyclometallated moiety, in the triplet state, they are nearly coplanar.

The transition corresponds in nearly all the cases to a LUMO+1→ HOMO transition. The former is centered in the alkynyl-R_2_ moiety, while the latter is centered in the cyclometallated ligand with some contribution of the metal for metal complexes; thus, this transition can be assigned to present a mixed ILCT/LLCT/MLCT character in nature. On the other hand, **1e** has a contribution from HOMO–LUMO and HOMO to LUMO–2 transitions. In this case, the HOMO is again based mainly in the alkynyl-R_2_ moiety, and the LUMO is centered in the cyclometallated ligand, so it corresponds to the LUMO+1 orbital of the remaining systems. The LUMO+2 orbital, in contrast, is centered mainly in the phenanthrenyl moiety. As an example, Figure S41 shows the orbitals LUMO+1 and HOMO that take part in this transition for complex **1a**.

### Aggregation-Induced Emission (AIE) Experiments

To try to induce the red-shifted emission in air-equilibrated samples, water was tested as a bad solvent to induce aggregation by preparing several acetonitrile/water mixtures. The absorption spectra of the compounds in acetonitrile and 90%:10% water/acetonitrile (Figure S42) show a change in the absorption band that presents a shoulder shifted at higher wavelengths. This suggests that upon the addition of water, new aggregated species is formed, which is present in the ground state.

The resulting behavior is observed to be strongly dependent on the alkynyl-R_2_ group. In general, the formation of a new band around 650 nm due to the formation of emissive aggregates can be observed. The assignment of this emission band to the excimer in acetonitrile is discarded since the latter appears at ca. 700 nm, as evidenced upon deoxygenation of the acetonitrile samples (Figure S43). Excitation spectra also support this assignment since they display a different profile from the recorded one for the monomer (Figure S44).

**Figure 7:**
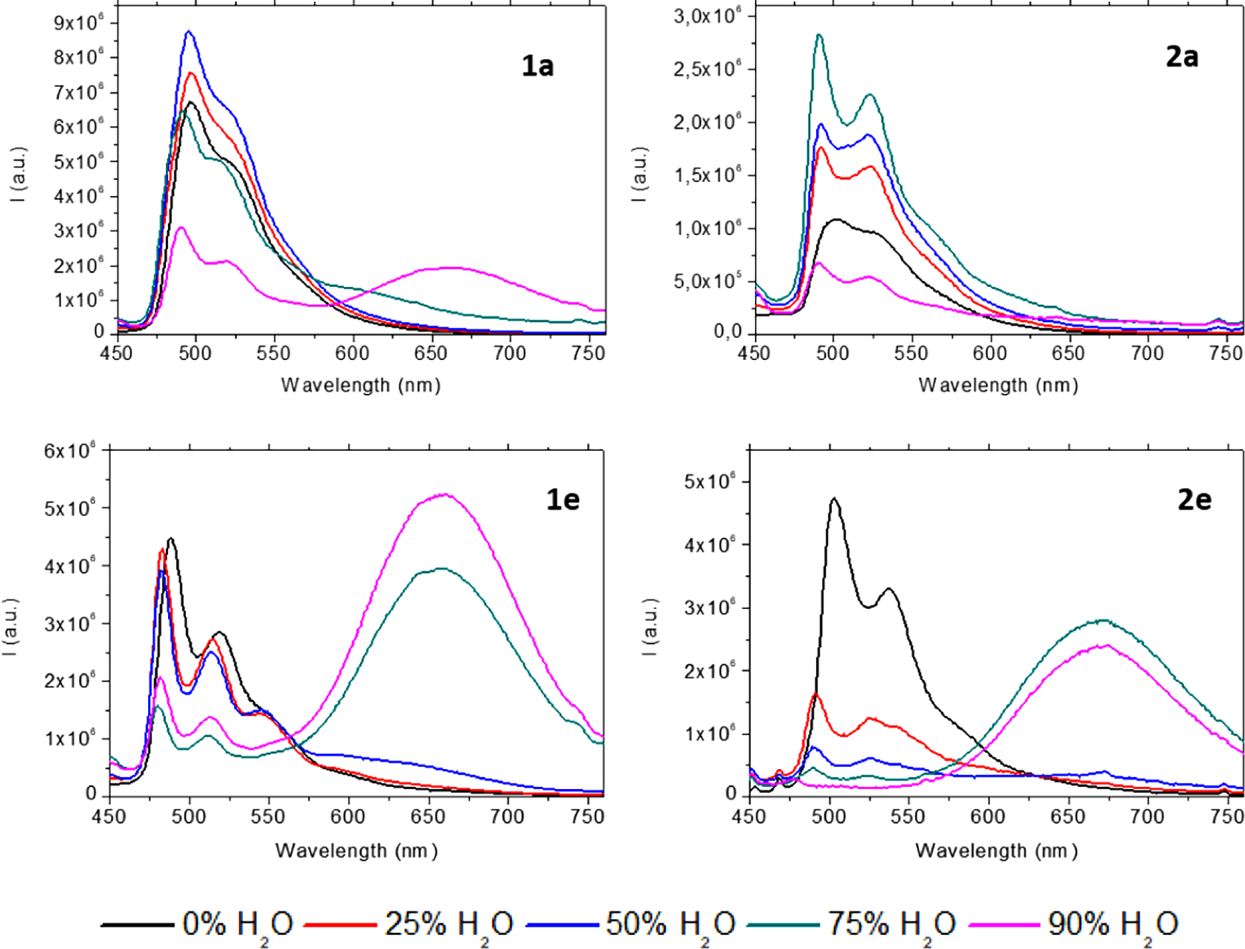
Emission spectra for acetonitrile/water mixtures of compounds **1a**, **1e**, **2a**, and **2e** at 298 K (λ_exc_ = 390 nm).

**Figure 8:**
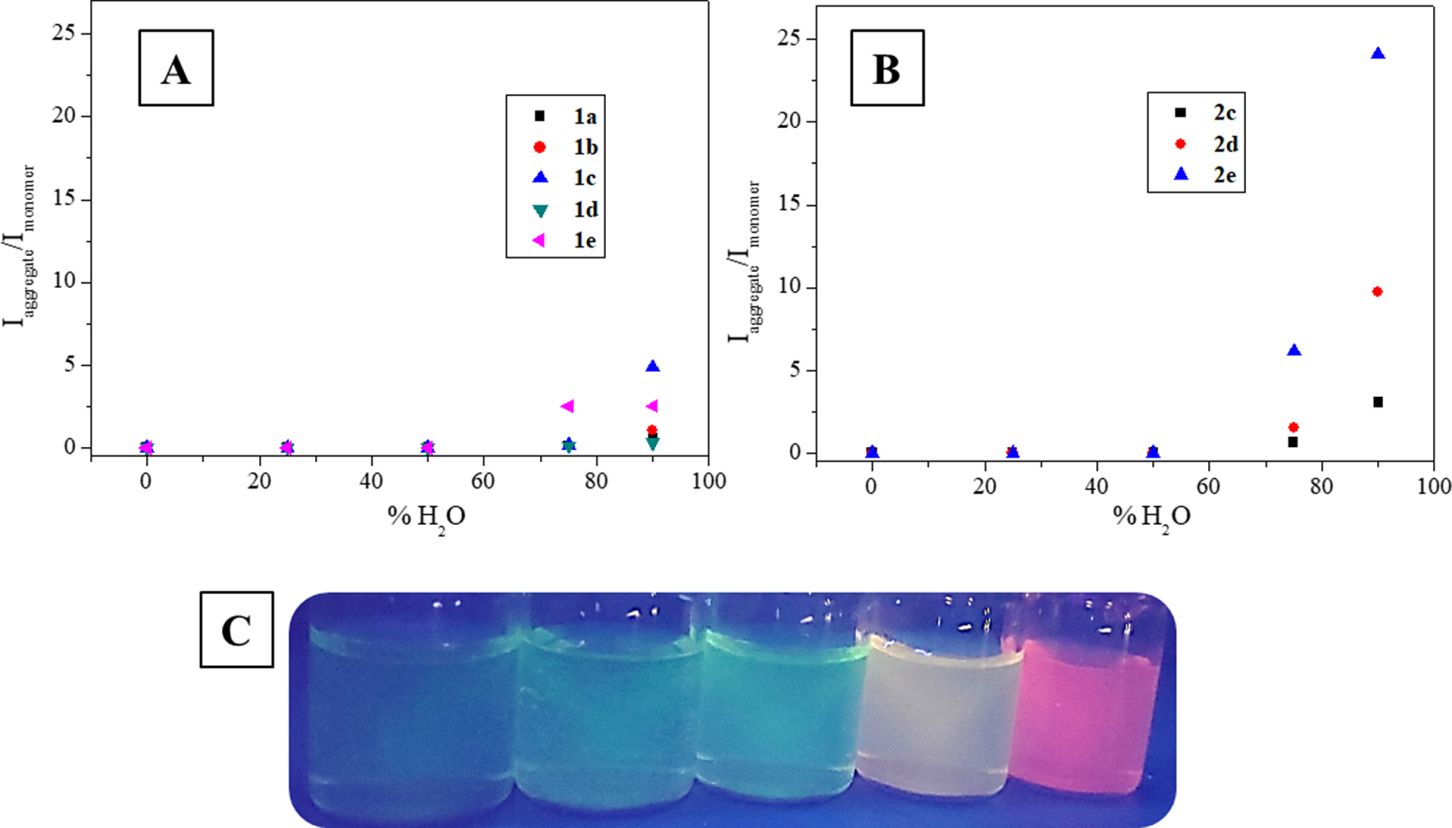
(A) Plot of the *I*_aggregates_/*I*_monomer_ of **series 1** complexes at different water contents (only compounds that aggregate are included in the plot). (B) Plot of the *I*_aggregates_/*I*_monomer_ of **series 2** complexes at different water contents (only compounds that aggregate are included in the plot). (C) Acetonitrile/water mixtures under the UV lamp for compound **2e** (increasing water content from left to right: 0, 25, 50, 75, and 90%).

The AIE band is enhanced when the percentage of water is higher than 75% (Figures 7, 8, and S45). At 90% water content, its emission reaches the maximum intensity, and, in some cases, it is predominant to the monomer, even becoming the sole emission in compound **2e** affording a completely red-shifted emission. In fact, AIE seems to be more favored in **series 2** complexes (Figure 8A,B) probably due to the presence of the fluoro substituent that can establish additional intermolecular contacts, favoring the aggregation process.

The total phosphorescence quantum yield increases when increasing the water percentage, as expected for an AIE behavior. This is also supported by the increasing contribution of the aggregates’ quantum yield when it is present by splitting the global QY value into the corresponding contributions of the monomer and the aggregated forms. The phosphorescence lifetime values of both monomer and aggregates become larger with increasing water content in all compounds, and, in all cases, τ_monomer_ > τ_aggregates_ (Table S6).

It can be observed that the non-radiative deactivation processes are more favored in both the monomer and the aggregates in agreement with the calculated *k*_r_ and *k*_nr_ values (Tables S7 and S8). Additionally, the more efficient AIE effect is directly related to the *k*_r_ rate constant. In the case of the aggregates, AIE behavior is directly related to an increase of *k*_r_ rate constants. A significant increase of *k*_r_ is detected (up to 5-fold in **series 1** and more than one order of magnitude in **series 2**) in the emission of the aggregates, while the contribution of *k*_nr_ is much less important. That is, the aggregation processes induced by the addition of water in the acetonitrile solutions favor the intermolecular contacts and the close distance between the molecules, making the environment more rigid and favoring the efficiency of the radiative emission process as it is evidenced in the increase of the *k*_r_ constants.

## Conclusions

N^C^N Pt(II)-cyclometallated compounds are emissive in both the solution and solid state with different emission origins. Room temperature phosphorescence emission is extremely favored in agreement with the recorded large Stokes shifts and long lifetimes. The presence of IR emission bands can be modulated by various methodologies including the formation of excimers and AIE to produce red-shifted emissions, where AIE can be favored by using a mixture of good and bad solvents, and excimer formation can be directly obtained in a homogeneous solution and the solid state (powder).

## Experimental Section

### General

Electrospray mass spectra were obtained at the Unitat d’Espectrometria de Masses (Universitat de Barcelona) in an LC/MSD-TOF spectrometer using H_2_O–CH_3_CN 1:1 to introduce the sample. IR spectra were recorded in KBr dispersion on an FT-IR 520 Nicolet spectrophotometer. NMR spectra were recorded in CDCl_3_ at the Unitat d’RMN of the Universitat de Barcelona with a Mercury 400 spectrometer (^1^H, 400 MHz; ^19^F, 376.5 MHz). Chemical shifts are given in δ values (ppm) relative to tetramethylsilane (^1^H) or CFCl_3_ (^19^F), and coupling constants *J* are given in Hz. Multiplicity is expressed as s (singlet), d (doublet), t (triplet), q (quadruplet), qi (quintuplet), and m (multiplet). Numbering schemes for the characterized compounds are displayed in Scheme 1.

UV–vis spectra were recorded in CH_2_Cl_2_ with a Cary 100 scan 388 Varian UV spectrometer. Emission and excitation spectra were recorded in a Horiba Jobin-Yvon SPEX Nanolog-TM spectrofluorometer at 298 K using 5 ×10^–5^ M solutions and in the solid state.

Emission quantum yields were determined with a Hamamatsu Quantaurus QY absolute photoluminescence quantum yield spectrometer C11347.

Luminescence lifetimes were measured on a JYF-DELTAPRO-NL equipment upon excitation of the samples with a 390 nm NanoLED and collecting the decays through a cut-off filter of 450 nm.

### Preparation of the Complexes

All reagents were obtained from commercial sources and used as received. Ligands 1,3-di(2-pyridyl)benzene (**L1**) and 2,2′-(5-fluoro-1,3-phenylene)dipyridine (**L2**) and platinum compounds **1**, **1a**, and **2** were prepared as reported elsewhere.^31,42^

General procedure for the synthesis of complexes **1x** and **2x**:^31^ a mixture of arylacetylene and sodium hydroxide was stirred at room temperature under an atmosphere of nitrogen for 30 min. The corresponding precursor **1** or **2** was added, and the mixture was further stirred for 24 h. The obtained solid was filtered under reduced pressure and washed with water, methanol, and hexane.

Compound (**2a**) was obtained as an orange solid from 0.025 g (0.052 mmol) of compound **2**, 0.011 g (0.104 mmol) of phenylacetylene, and 0.004 g (0.104 mmol) of sodium hydroxide. Yield: 0.017 g (61%).

^1^H NMR (CDCl_3_, 400 MHz): δ 9.52 [dd, 2H, ^3^*J*(Pt-H) = 47.6, ^3^*J*(H-H) = 5.6, ^4^*J*(H-H) = 1.6, H^f^]; 7.96 [td, 2H, ^3^*J*(H-H) = 8.0, ^4^*J*(H-H) = 1.6, H^d^]; 7.64 [d, 2H, ^3^*J*(H-H) = 7.6, H^Ph^]; 7.58 [dd, 2H, ^3^*J*(H-H) = 8.0, ^4^*J*(H-H) = 1.1, H^c^]; 7.27 [d, 2H, ^3^*J*(F-H) = 10.8, H^b^]; 7.23–7.26 [m, 4H, H^e,Ph^]; 7.19 [t, 1H, ^3^*J*(H-H) = 7.6, H^Ph^]. ^19^F NMR (CDCl_3_, 376.5 MHz): δ −118.62 [t, 1H, ^3^*J*(H-F) = 9.9]. MS-ESI^+^: *m*/*z* 989.13 [2M-C_8_H_5_]^+^, 546.09 [M + H]^+^, 444.05 [M-C_8_H_5_]^+^. IR: ν 2085.34 (C≡C).

Compound (**1b**) was obtained as an orange solid from 0.020 g (0.043 mmol) of compound **1**, 0.010 g (0.086 mmol) of 1-ethynyl-4-fluorobenzene, and 0.004 g (0.086 mmol) of sodium hydroxide. Yield: 0.017 g (71%).

^1^H NMR (CDCl_3_, 400 MHz): δ 9.49 [d, 2H, ^3^*J*(Pt-H) = 47.6, ^3^*J*(H-H) = 5.5, H^f^]; 7.93 [td, 2H, ^3^*J*(H-H) = 7.8, ^4^*J*(H-H) = 1.4, H^d^]; 7.69 [d, 2H, ^3^*J*(H-H) = 7.8, H^c^]; 7.51–7.56 [m, 4H, H^b,Ph-F^]; 7.24–7.26 [m, 3H, H^a,e^]; 6.97 [t, 1H, ^3^*J*(F-H) = ^3^*J*(H-H) = 8.8, H^Ph-F^]. ^19^F NMR (CDCl_3_, 376.5 MHz): δ −116.03 [m, 1F]. MS-ESI^**+**^: *m*/*z* 971.14 [2M-C_8_H_4_F]^+^, 546.09 [M + H]^+^, 426.06 [M-C_8_H_4_F]^+^. IR: ν 2081.07 (C≡C).

Compound (**2b**) was obtained as an orange solid from 0.020 g (0.042 mmol) of compound **2**, 0.010 g (0.084 mmol) of 1-ethynyl-4-fluorobenzene, and 0.003 g (0.084 mmol) of sodium hydroxide. Yield: 0.013 g (54%).

^1^H NMR (CDCl_3_, 400 MHz): δ 9.50 [d, 2H, ^3^*J*(Pt-H) = 48.0, ^3^*J*(H-H) = 5.5, H^f^]; 7.97 [td, 2H, ^3^*J*(H-H) = 7.9, ^4^*J*(H-H) = 1.2, H^d^]; 7.65 [d, 2H, ^3^*J*(H-H) = 7.7, H^c^]; 7.53 [dd, 2H, ^3^*J*(H-H) = 8.5, ^4^*J*(F-H) = 5.6, H^Ph-F^]; 7.28 [d, 2H, ^3^*J*(F-H) = 10.1, H^b^]; 7.24–7.26 [m, 2H, H^e^]; 6.97 [t, 1H, ^3^*J*(F-H) = ^3^*J*(H-H) = 8.9, H^Ph-F^]. ^19^F NMR (CDCl_3_, 376.5 MHz): δ −115.88 [m, 1F], −118.14 [t, 1F, ^3^*J*(H-F) = 10.0]. MS-ESI^**+**^: *m*/*z* 1007.12 [2M-C_8_H_4_F]^+^, 564.08 [M + H]^+^, 444.05 [M-C_8_H_4_F]^+^. IR: ν 2085.34 (C≡C).

Compound (**1c**) was obtained as an orange solid from 0.020 g (0.043 mmol) of compound **1**, 0.009 g (0.086 mmol) of 3-ethynylthiophene, and 0.004 g (0.086 mmol) of sodium hydroxide. Yield: 0.014 g (61%).

^1^H NMR (CDCl_3_, 400 MHz): δ 9.50 [dd, 2H, ^3^*J*(Pt-H) = 47.6, ^3^*J*(H-H) = 5.7, ^4^*J*(H-H) = 1.6, H^f^]; 7.93 [td, 2H, ^3^*J*(H-H) = 7.6, ^4^*J*(H-H) = 1.6, H^d^]; 7.68 [d, 2H, ^3^*J*(H-H) = 8.0, H^c^]; 7.52 [d, 2H, ^3^*J*(H-H) = 7.7, H^b^]; 7.36 [dd, 1H, ^3^*J*(H-H) = 2.8, ^4^*J*(H-H) = 1.3, H^Thio^]; 7.18–7.26 [m, 5H, H^a,e,Thio^]. MS-ESI^**+**^: *m*/*z* 959.11 [2M-C_6_H_3_S]^+^, 534.06 [M + H]^+^, 426.06 [M-C_6_H_3_S]^+^. IR: ν 2076.81 (C≡C).

Compound (**2c**) was obtained as an orange solid from 0.020 g (0.042 mmol) of compound **2**, 0.009 g (0.084 mmol) of 3-ethynylthiophene, and 0.003 g (0.084 mmol) of sodium hydroxide. Yield: 0.015 g (65%).

^1^H NMR (CDCl_3_, 400 MHz): δ 9.51 [dd, 2H, ^3^*J*(Pt-H) = 48.4, ^3^*J*(H-H) = 5.6, ^4^*J*(H-H) = 1.6, H^f^]; 7.96 [td, 2H, ^3^*J*(H-H) = 7.8, ^4^*J*(H-H) = 1.7, H^d^]; 7.64 [d, 2H, ^3^*J*(H-H) = 7.9, H^c^]; 7.36 [dd, 1H, ^3^*J*(H-H) = 2.6, ^4^*J*(H-H) = 1.5, H^Thio^]; 7.28 [d, 2H, ^3^*J*(F-H) = 10.1, H^b^]; 7.21–7.25 [m, 4H, H^e,Thio^]. ^19^F NMR (CDCl_3_, 376.5 MHz): δ −118.47 [t, 1H, ^3^*J*(H-F) = 10.0]. MS-ESI^**+**^: *m*/*z* 995.09 [2M-C_6_H_3_S]^+^, 552.05 [M + H]^+^, 444.05 [M-C_6_H_3_S]^+^ IR: ν 2081.07 (C≡C).

Compound (**1d**) was obtained as an orange solid from 0.020 g (0.043 mmol) of compound **1**, 0.013 g (0.086 mmol) of 2-ethynylnaphthalene, and 0.004 g (0.086 mmol) of sodium hydroxide. Yield: 0.020 g (80%).

^1^H NMR (CDCl_3_, 400 MHz): δ 9.56 [dd, 2H, ^3^*J*(Pt-H) = 47.2, ^3^*J*(H-H) = 5.7, ^4^*J*(H-H) = 1.5, H^f^]; 8.05 [s, 1H, H^Naph^]; 7.94 [td, 2H, ^3^*J*(H-H) = 7.8, ^4^*J*(H-H) = 1.6, H^d^], 7.73–7.81 [m, 3H, H^Naph^]; 7.67–7.72 [m, 3H, H^c,Naph^], 7.54 [d, 2H, ^3^*J*(H-H) = 7.7, H^b^]; 7.34–7.46 [m, 2H, H^Naph^]; 7.21–7.26 [m, 3H, H^a,e^]. **MS-ESI^+^**: *m*/*z* 1003.16 [2M-C_12_H_7_]^+^, 578.12 [M + H]^+^, 426.06 [M-C_12_H_7_]^+^. **IR**: ν 2085.34 (C≡C).

Compound (**2d**) was obtained as an orange solid from 0.015 g (0.033 mmol) of compound **2**, 0.010 g (0.066 mmol) of 2-ethynylnaphthalene, and 0.003 g (0.066 mmol) of sodium hydroxide. Yield: 0.012 g (61%).

^1^H NMR (CDCl_3_, 400 MHz): δ 9.57 [dd, 2H, ^3^*J*(Pt-H) = 48.0, ^3^*J*(H-H) = 5.7, ^4^*J*(H-H) = 1.5, H^f^]; 8.05 [s, 1H, H^Naph^]; 7.98 [td, 2H, ^3^*J*(H-H) = 7.8, ^4^*J*(H-H) = 1.6, H^d^], 7.77–7.79 [m, 3H, H^Naph^]; 7.66–7.68 [m, 3H, H^c,Naph^]; 7.42 [m, 2H, H^Naph^]; 7.29 [d, 2H, ^3^*J*(F-H) = 10.0, H^b^]; 7.26–7.29 [m, 2H, H^e^]. ^19^F NMR (CDCl_3_, 376.5 MHz): δ −118.47 [t, 1H, ^3^*J*(H-F) = 10.0]. MS-ESI^**+**^: *m*/*z* 10039.15 [2M-C_12_H_7_]^+^, 596.11 [M + H]^+^, 444.05 [M-C_12_H_7_]^+^. IR: ν 2085.34 (C≡C).

Compound (**1e**) was obtained as an orange solid from 0.020 g (0.043 mmol) of compound **1**, 0.017 g (0.086 mmol) of 9-ethynylphenanthrene, and 0.004 g (0.086 mmol) of sodium hydroxide. Yield: 0.017 g (63%).

^1^H NMR (CDCl_3_, 400 MHz): δ 9.63 [dd, 2H, ^3^*J*(Pt-H) = 48.0, ^3^*J*(H-H) = 5.7, ^4^*J*(H-H) = 1.6, H^f^]; 8.98 [m, 1H, H^Phen^]; 8.71 [m, 1H, H^Phen^]; 8.65 [d, 1H, ^3^*J*(H-H) = 7.9, H^Phen^]; 8.08 [s, 1H, H^Phen^]; 7.95 [td, 2H, ^3^*J*(H-H) = 7.8, ^4^*J*(H-H) = 1.6, H^d^], 7.84 [dd, 1H, ^3^*J*(H-H) = 7.6, ^4^*J*(H-H) = 1.6, H^Phen^], 7.72 [d, 2H, ^3^*J*(H-H) = 7.8, H^c^]; 7.66 [m, 2H, H^Phen^]; 7.53–7.61 [m, 3H, H^b,Phen^]; 7.19–7.25 [m, 3H, H^a,e^]. MS-ESI^**+**^: *m*/*z* 1053.18 [2M-C_16_H_9_]^+^, 628.14 [M + H]^+^, 426.06 [M-C_16_H_9_]^+^. IR: ν 2064.01 (C≡C).

Compound (**2e**) was obtained as an orange solid from 0.020 g (0.042 mmol) of compound **2**, 0.017 g (0.084 mmol) of 9-ethynylphenanthrene, and 0.004 g (0.084 mmol) of sodium hydroxide. Yield: 0.020 g (74%).

^1^H NMR (CDCl_3_, 400 MHz): δ 9.64 [dd, 2H, ^3^*J*(Pt-H) = 48.4, ^3^*J*(H-H) = 5.7, ^4^*J*(H-H) = 1.5, H^f^]; 8.97 [m, 1H, H^Phen^]; 8.71 [m, 1H, H^Phen^]; 8.65 [d, 1H, ^3^*J*(H-H) = 7.9, H^Phen^]; 8.08 [s, 1H, H^Phen^]; 7.98 [td, 2H, ^3^*J*(H-H) = 7.8, ^4^*J*(H-H) = 1.6, H^d^], 7.84 [dd, 1H, ^3^*J*(H-H) = 7.3, ^4^*J*(H-H) = 2.0, H^Phen^], 7.67 [m, 4H, H^c,Phen^]; 7.57 [m, 2H, H^Phen^]; 7.31 [d, 2H, ^3^*J*(F-H) = 10.0, H^b^]; 7.24–7.28 [m, 2H, H^e^]. ^19^F NMR (CDCl_3_, 376.5 MHz): δ −118.35 [t, 1H, ^3^*J*(H-F) = 10.0]. MS-ESI^**+**^: *m*/*z* 1089.16 [2M-C_16_H_9_]^+^, 646.12 [M + H]^+^, 444.05 [M-C_16_H_9_]^+^. IR: ν 2072.54 (C≡C).

### X-ray Diffraction

Single crystals suitable for X-ray diffraction analysis were grown for **1e** and **2c** by slow diffusion of methanol or hexane, respectively, in a dichloromethane solution of the compounds.

Single-crystal X-ray data for **1e** and **2c** were obtained using a Bruker-Nonius Kappa CCD diffractometer with an APEX-II detector with graphite-monochromatized Mo Kα (λ = 0.71073 Å) radiation. Data collection and reduction were performed using the program *COLLECT*(43) and *HKL DENZO AND SCALEPACK*,^44^ respectively, and the intensities were corrected for absorption using *SADABS*.^45^ The structures were solved with intrinsic phasing (*SHELXT*)^46^ and refined by full-matrix least squares on *F*^2^ using the *OLEX2* software,^47^ which utilizes the *SHELXL* module.^48^

## Computational Details

Theoretical calculations were performed at the DFT level using Q-chem 5.1,^49^ included in Spartan 20.^50^ The functional chosen was B3LYP,^51^ and the basis set was chosen as follows: 6-31G* for C, H, N, and Cl, including polarization functions for non-hydrogen atoms,^52^ and LANL2DZ^53^ for Pt. Solvent effects were considered using the CPCM model.^54^ No symmetry restrictions were imposed. Optimized geometries are given in Table S9.

## Supporting Information Available

Characterization data of the compounds; X-ray packing images of **1e** and **2c**; absorption, emission, and excitation spectra of the compounds in dichloromethane and in the solid state; absorption, emission, and excitation spectra of the compounds in acetonitrile/water mixtures; DFT optimized geometries in solution of the compounds; orbitals that participate in the UV–vis transitions; crystal data and structure refinement tables; radiative and non-radiative rate constants obtained in air-equilibrated (with O_2_) or degassed (N_2_ sat.) dichloromethane and acetonitrile/water solutions; phosphorescence quantum yields and lifetimes recorded in aerated acetonitrile/water mixtures; key geometrical parameters optimized at the DFT level in solution; and experimental and calculated UV–vis and emission transitions (PDF)
