# Supplementary material for: Toward Near-Infrared Emission in Pt(II)-Cyclometallated Compounds: From Excimers’ Formation to Aggregation-Induced Emission
Source: Inorg Chem. 2023 Jan 25;62(5):2000–12. doi: 10.1021/acs.inorgchem.2c03490 (PMC9906741; doi:10.1021/acs.inorgchem.2c03490)
Supplement: Supplementary file 1 — ic2c03490_si_001.pdf [file ic2c03490_si_001.pdf]

Supporting Information

Towards NIR emission in Pt(II)-cyclometallated compounds. From excimers' formation to AIE.

Ariadna Lázaro,[a,b] Ramon Bosque,[a] Jas S. Ward,[c] Kari Rissanen,[c] Margarita Crespo,[a,d] Laura Rodríguez[a,b*]

[a] *Departament de Química Inorgànica i Orgànica, Secció de Química Inorgànica, Universitat de Barcelona, Martí i Franquès 1-11, E-08028 Barcelona, Spain. e-mail: laura.rodriguez@qi.ub.es*

[b] *Institut de Nanociència i Nanotecnologia (IN2UB). Universitat de Barcelona, 08028 Barcelona, Spain*

[c] *Department of Chemistry, P.O. Box 35, 40014, Jyväskylä, Finland*

[d] *Institut de Biomedicina de la Universitat de Barcelona (IBUB), 08028-Barcelona, Spain*

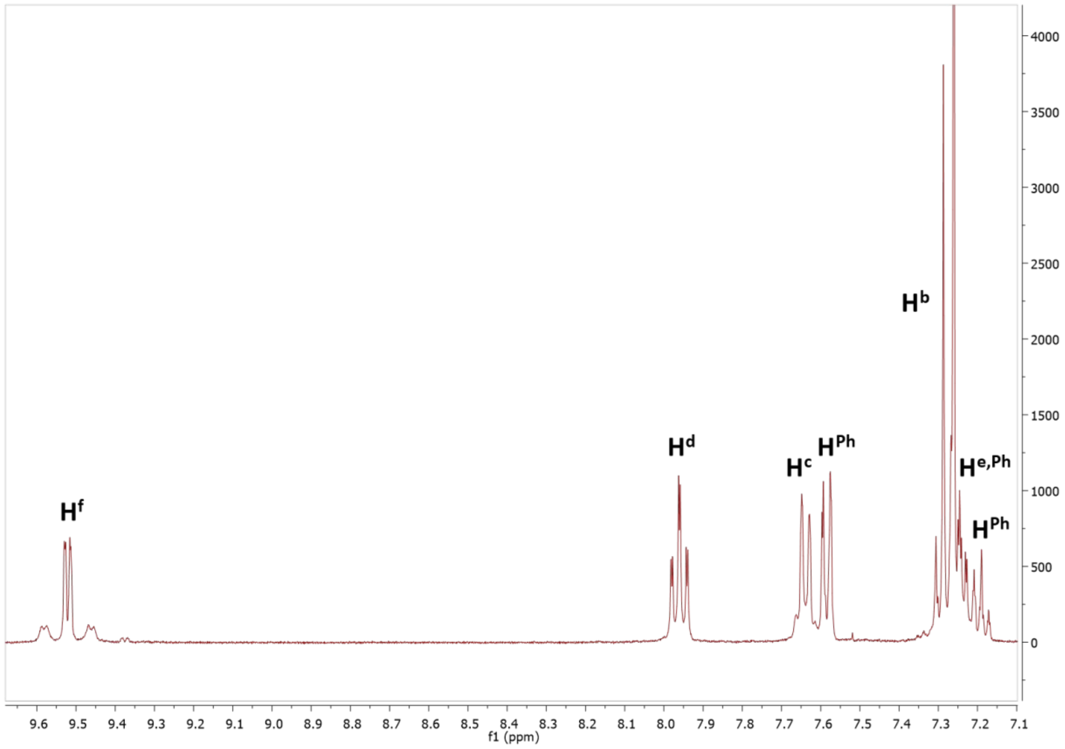

Figure S1. ^{1}H NMR spectrum of compound **2a** in $CDCl_3$.

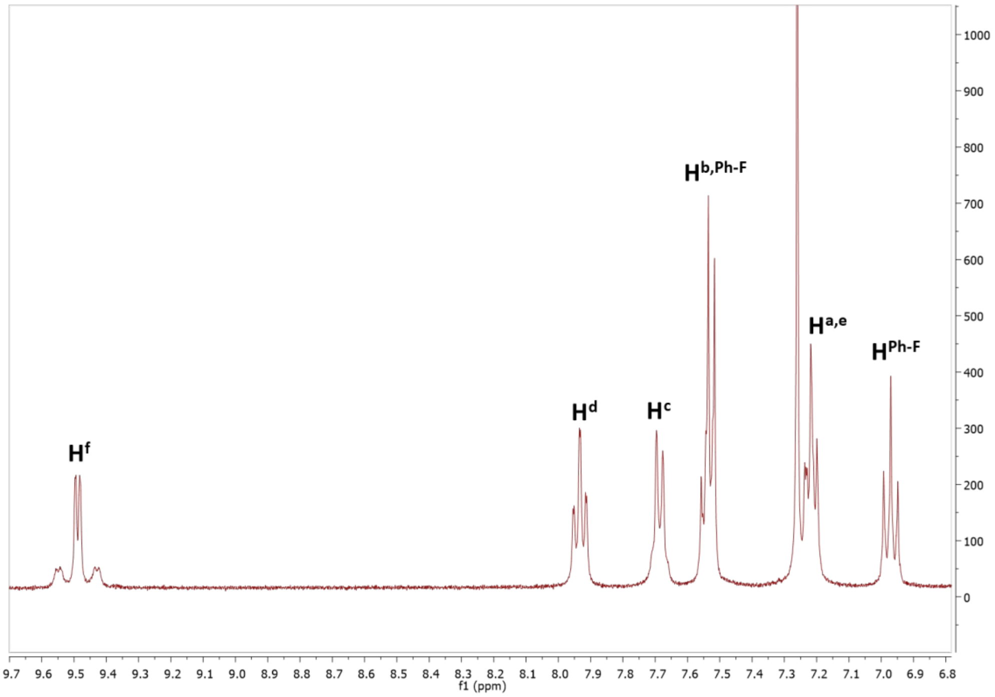

Figure S2. ^{1}H NMR spectrum of compound **1b** in $CDCl_3$.

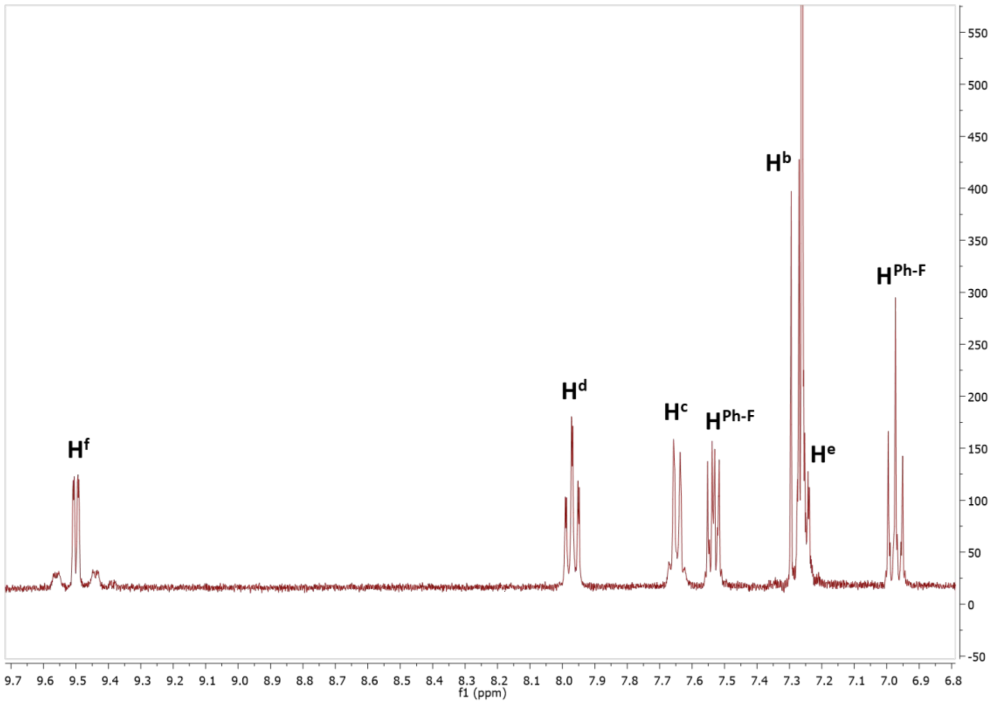

Figure S3. ^{1}H NMR spectrum of compound **2b** in $CDCl_3$.

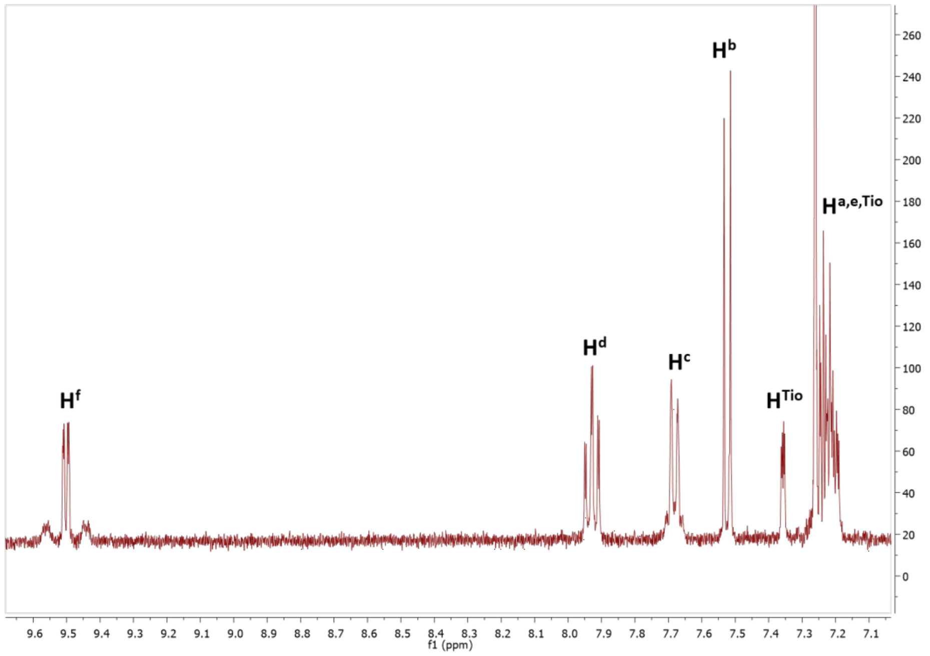

Figure S4. ^{1}H NMR spectrum of compound **1c** in $CDCl_3$.

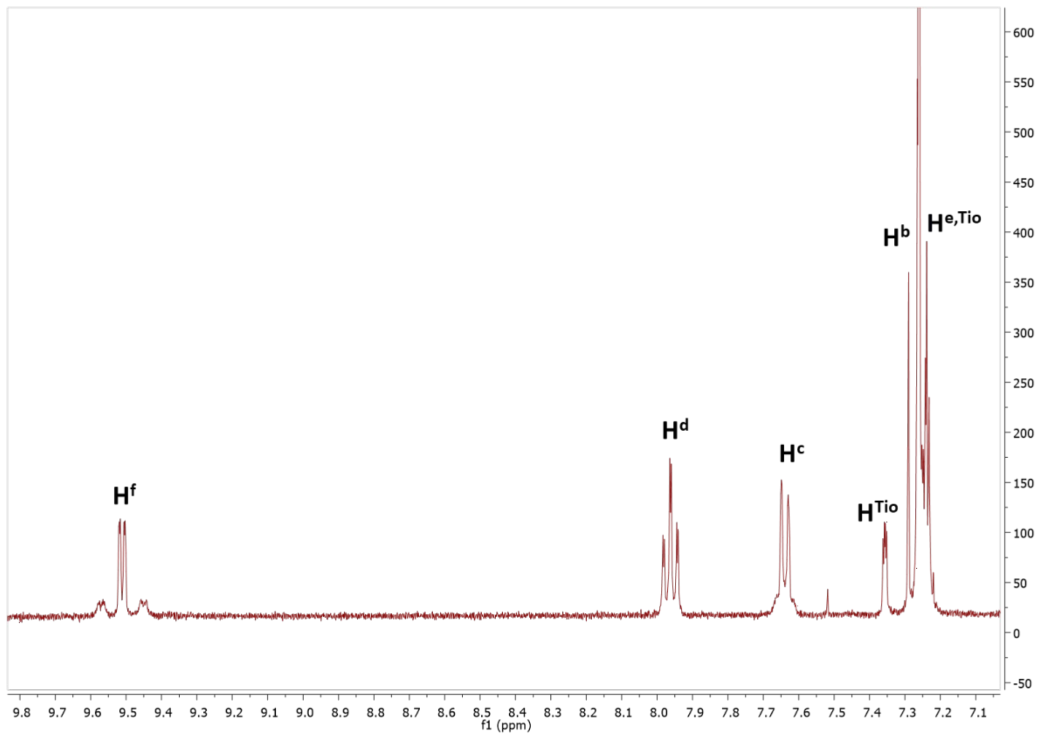

Figure S5. ^{1}H NMR spectrum of compound **2c** in $CDCl_3$.

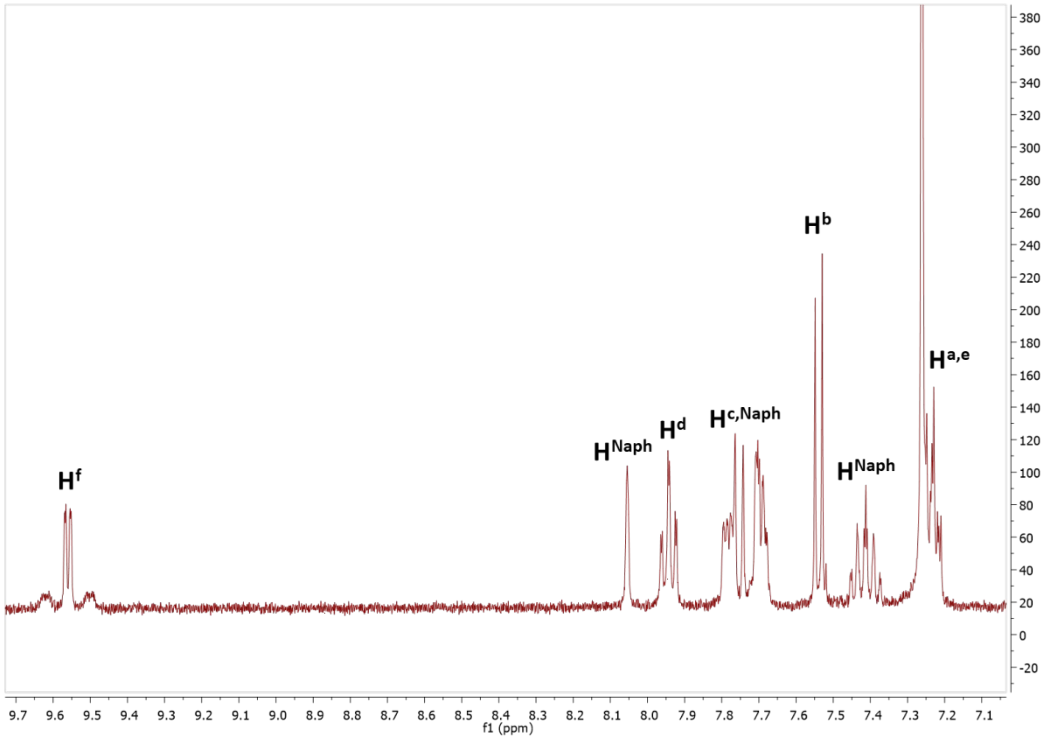

Figure S6. ^{1}H NMR spectrum of compound **1d** in $CDCl_3$.

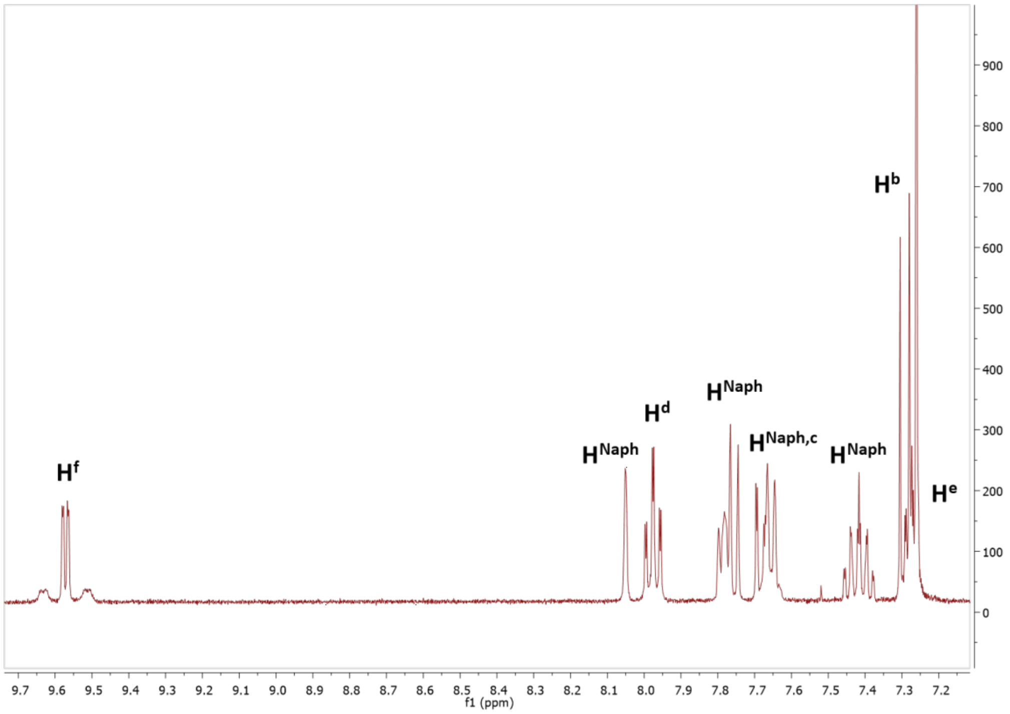

Figure S7. ^{1}H NMR spectrum of compound **2d** in $CDCl_3$.

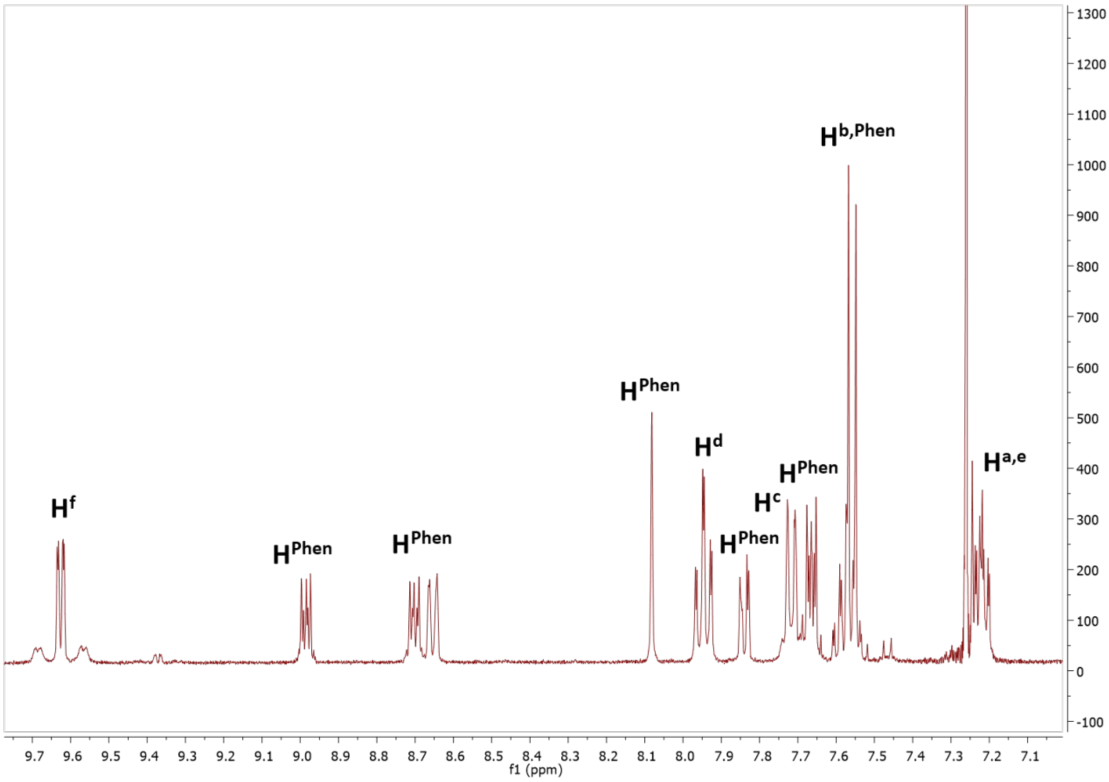

Figure S8. ^{1}H NMR spectrum of compound **1e** in $CDCl_3$.

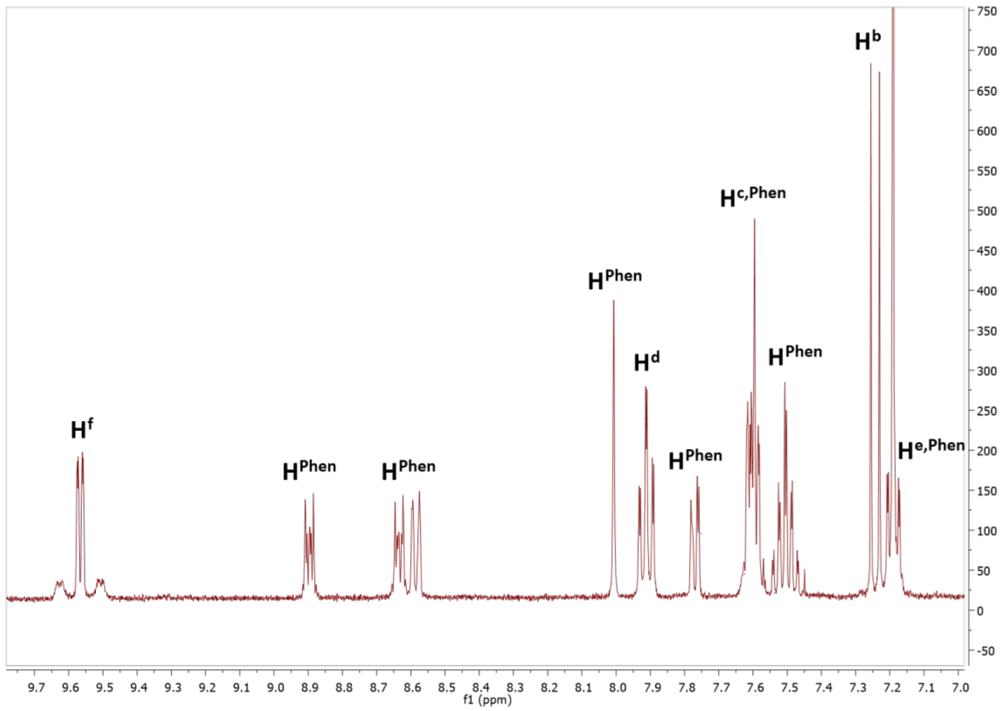

Figure S9. ^{1}H NMR spectrum of compound **2e** in $CDCl_3$.

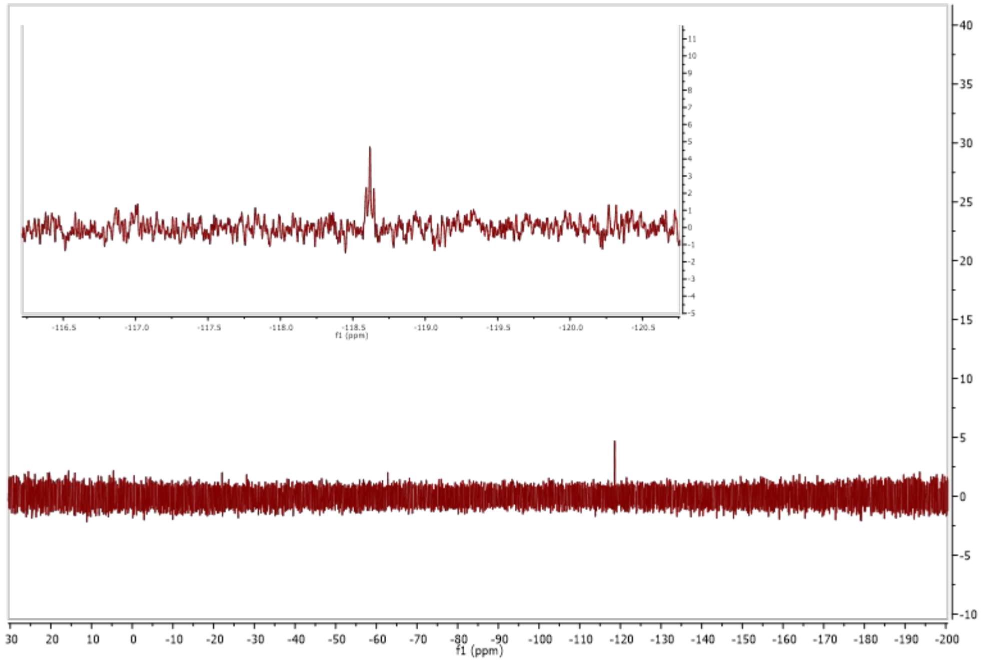

Figure S10. ^{19}F NMR spectrum of compound **2a** in $CDCl_3$.

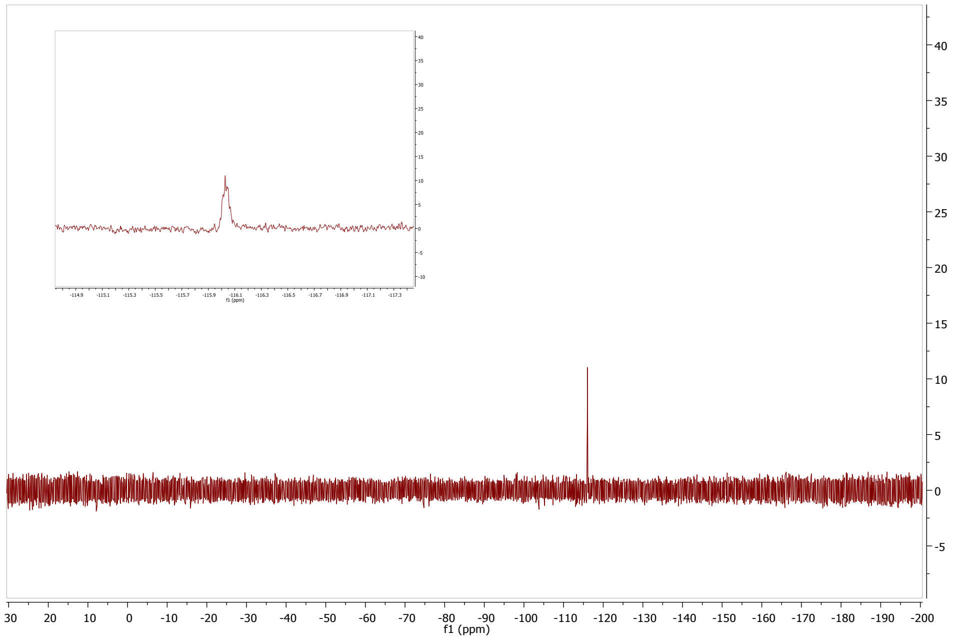

Figure S11. ^{19}F NMR spectrum of compound **1b** in $CDCl_3$.

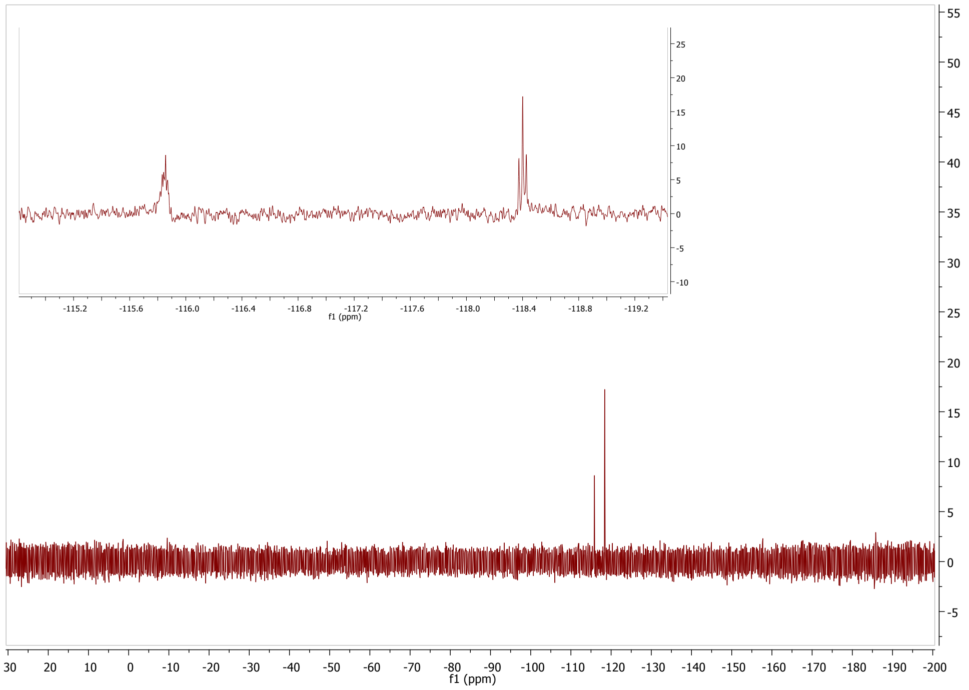

Figure S12. ^{19}F NMR spectrum of compound **2b** in $CDCl_3$.

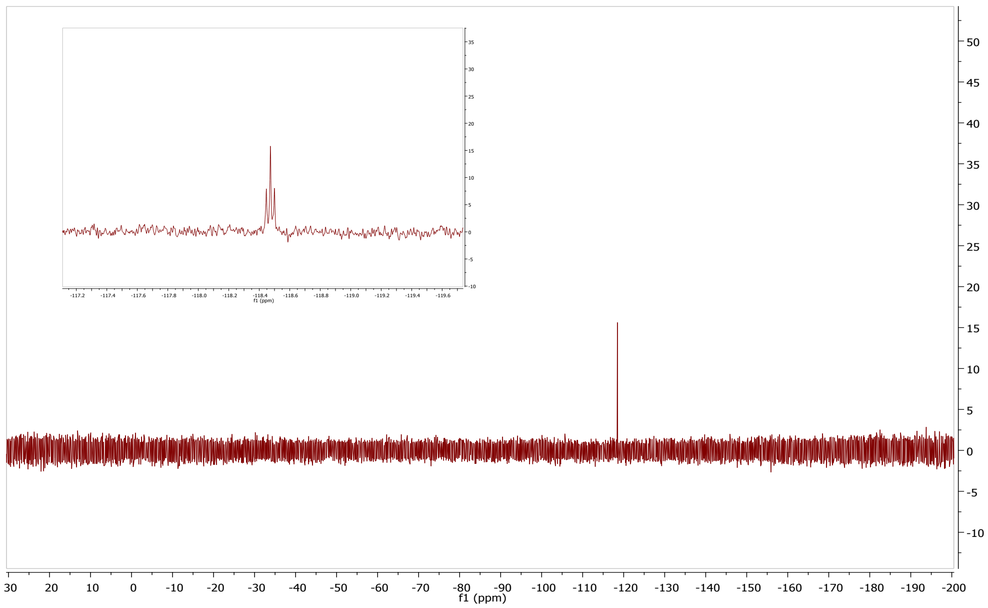

Figure S13. ^{19}F NMR spectrum of compound **2c** in $CDCl_3$.

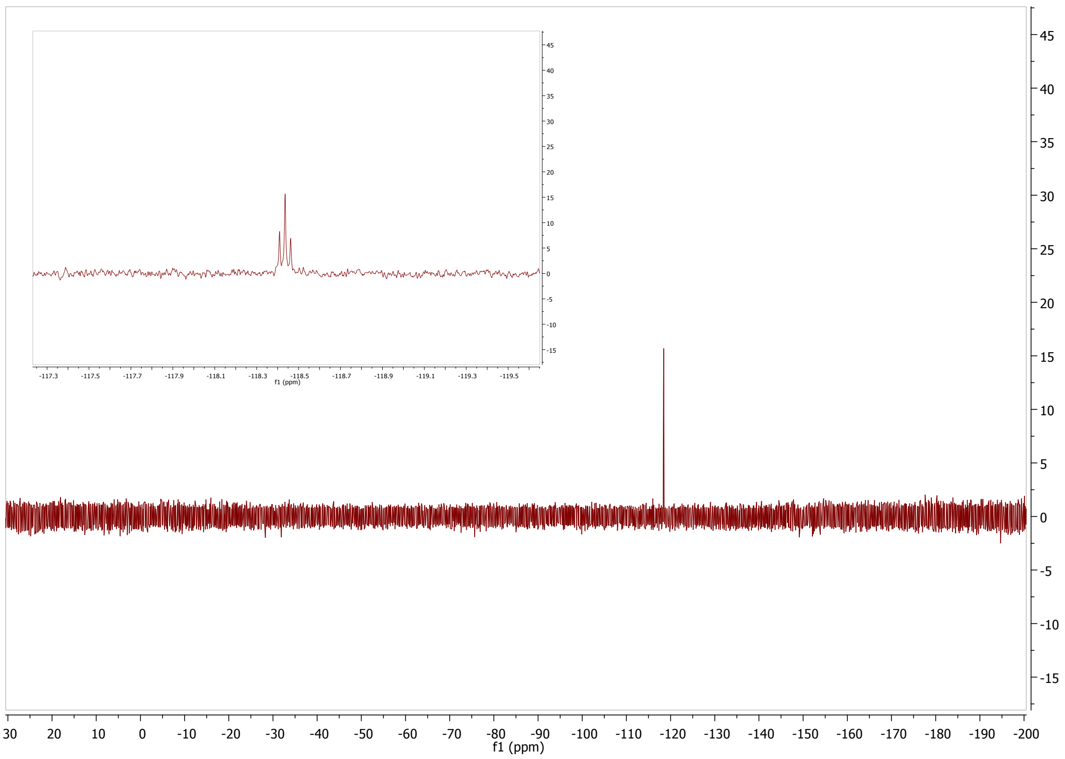

Figure S14. ^{19}F NMR spectrum of compound **2d** in $CDCl_3$.

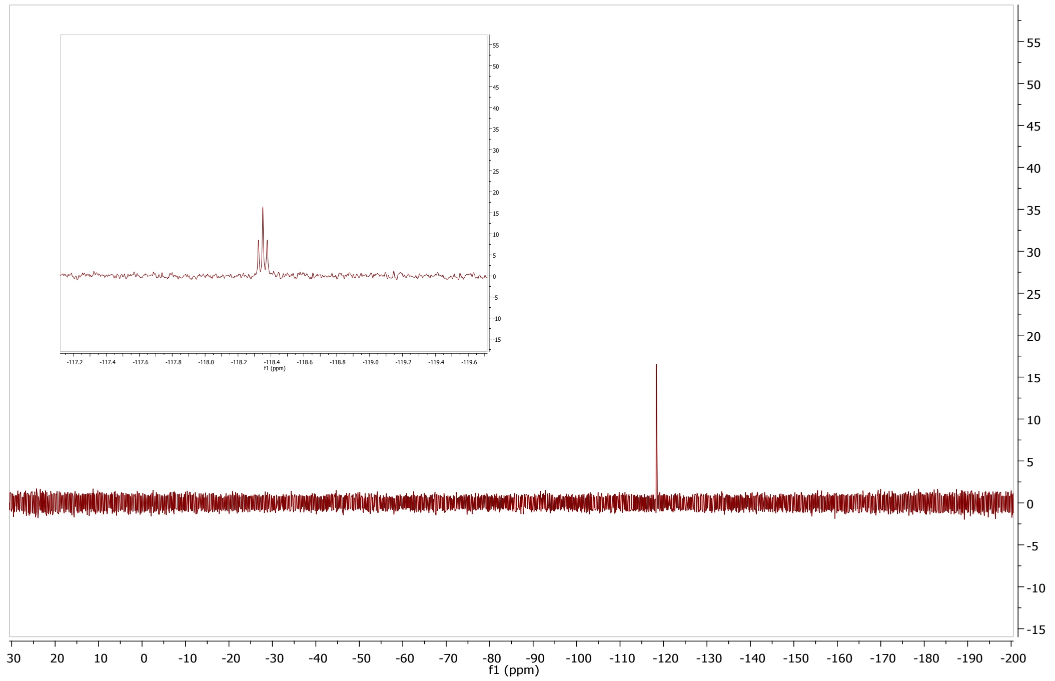

Figure S15. ^{19}F NMR spectrum of compound **2e** in $CDCl_3$.

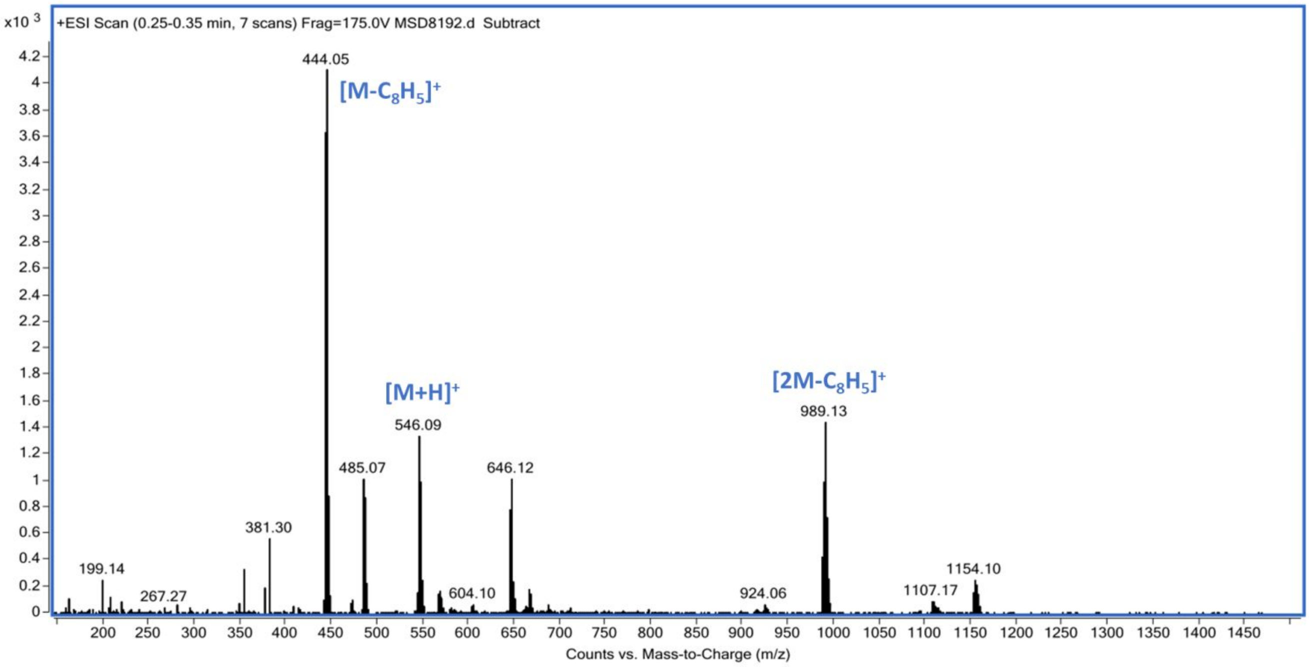

Figure S16. ESI-MS(+) spectrum of **2a**.

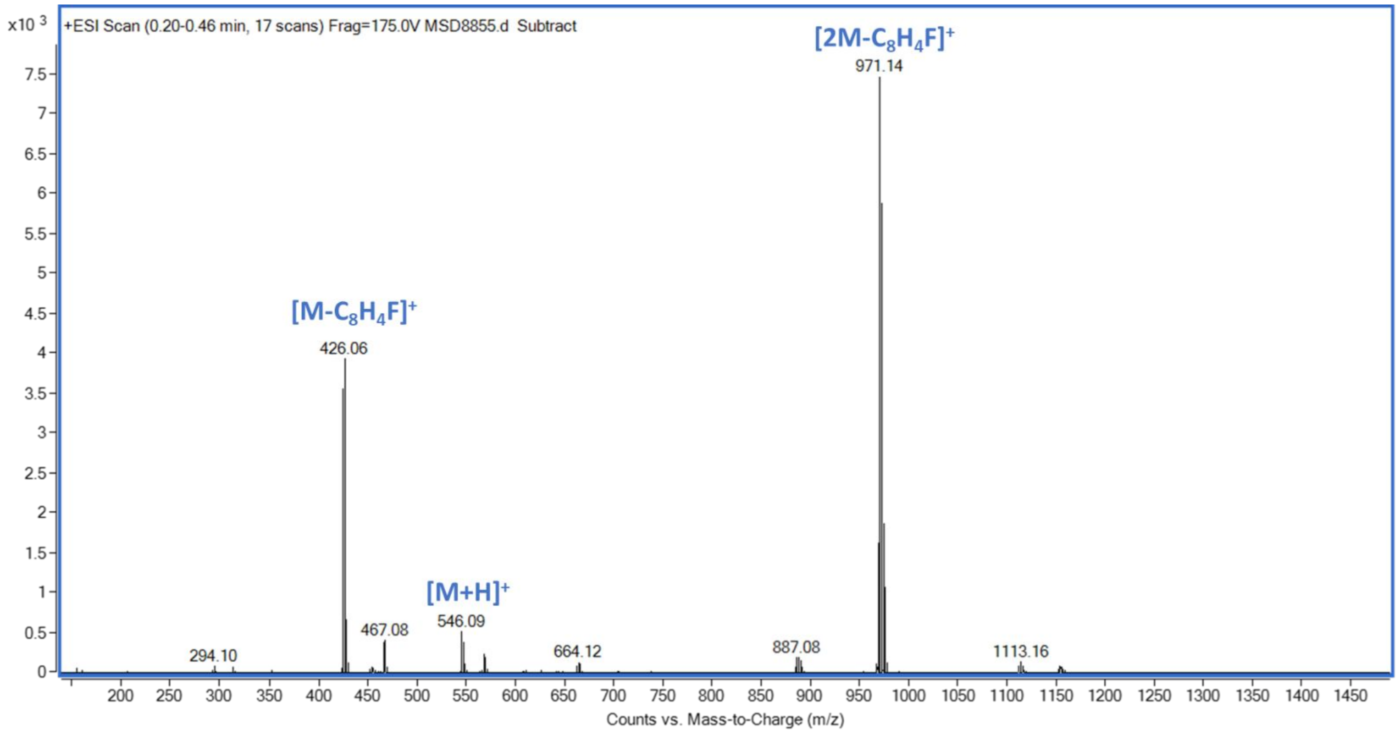

Figure S17. ESI-MS(+) spectrum of **1b**.

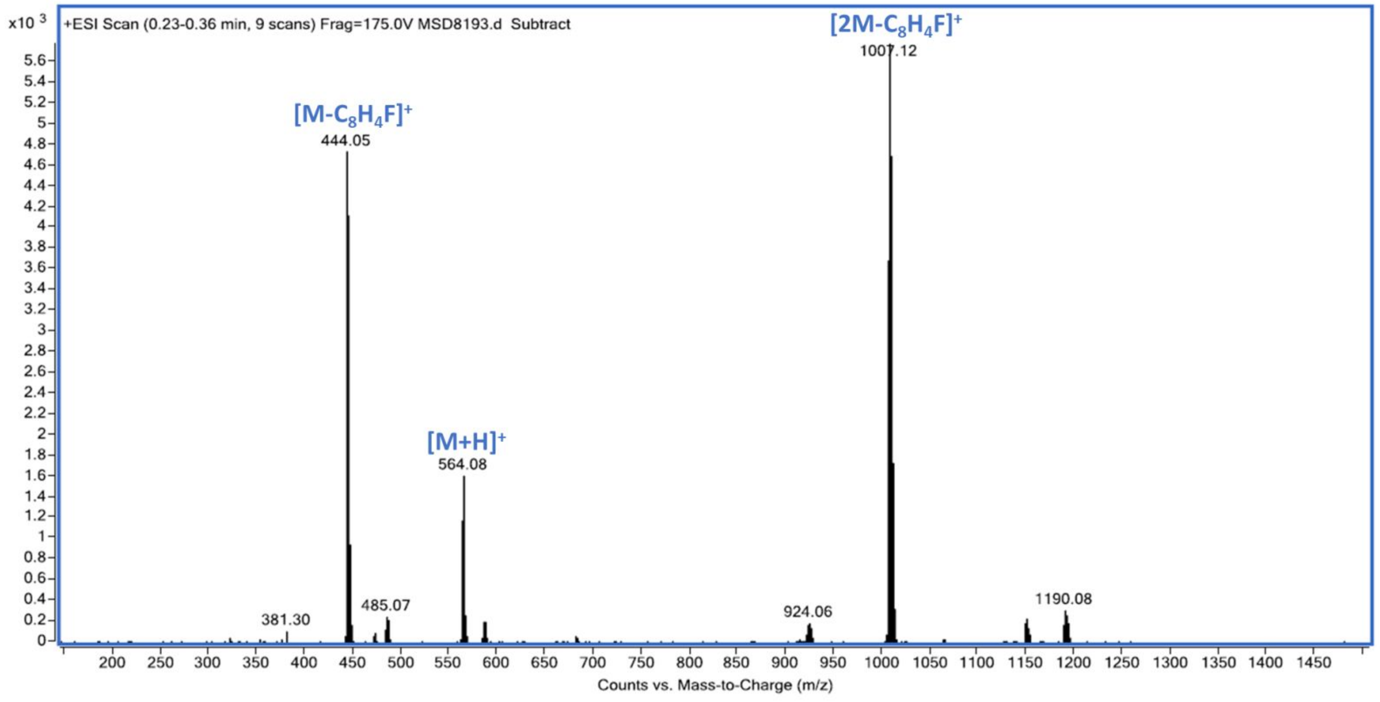

Figure S18. ESI-MS(+) spectrum of **2b**.

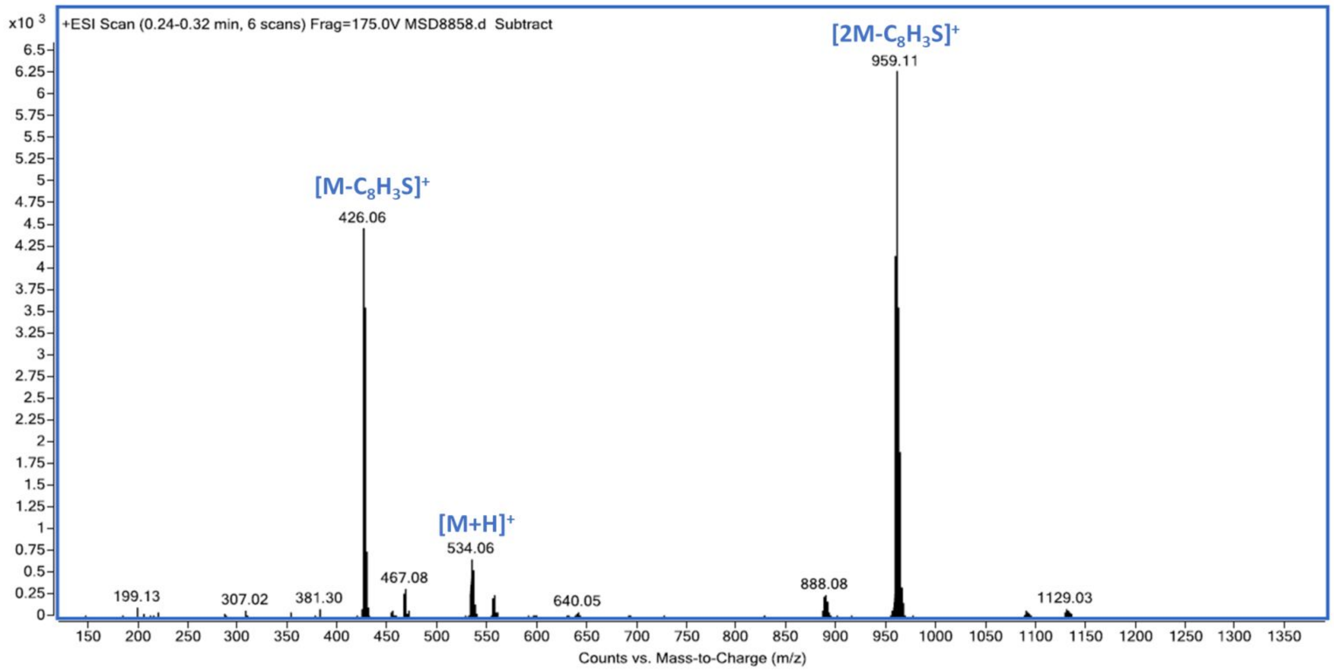

Figure S19. ESI-MS(+) spectrum of **1c**.

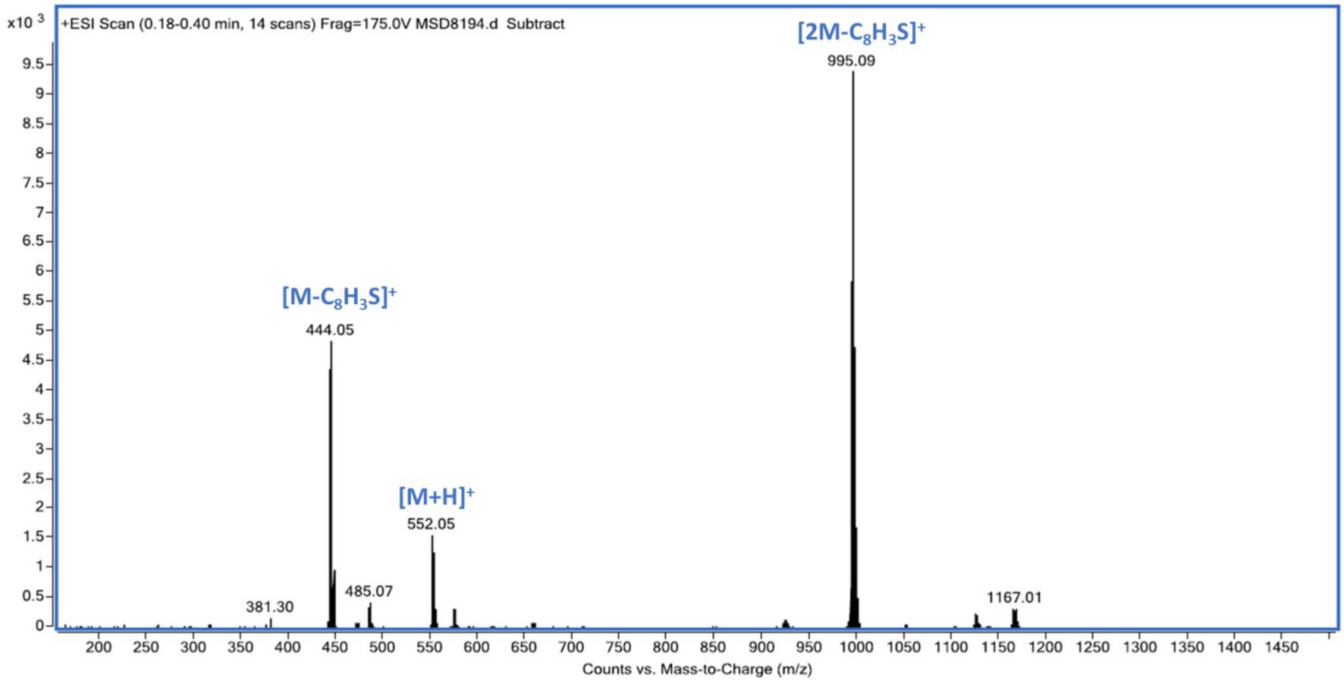

Figure S20. ESI-MS(+) spectrum of **2c**.

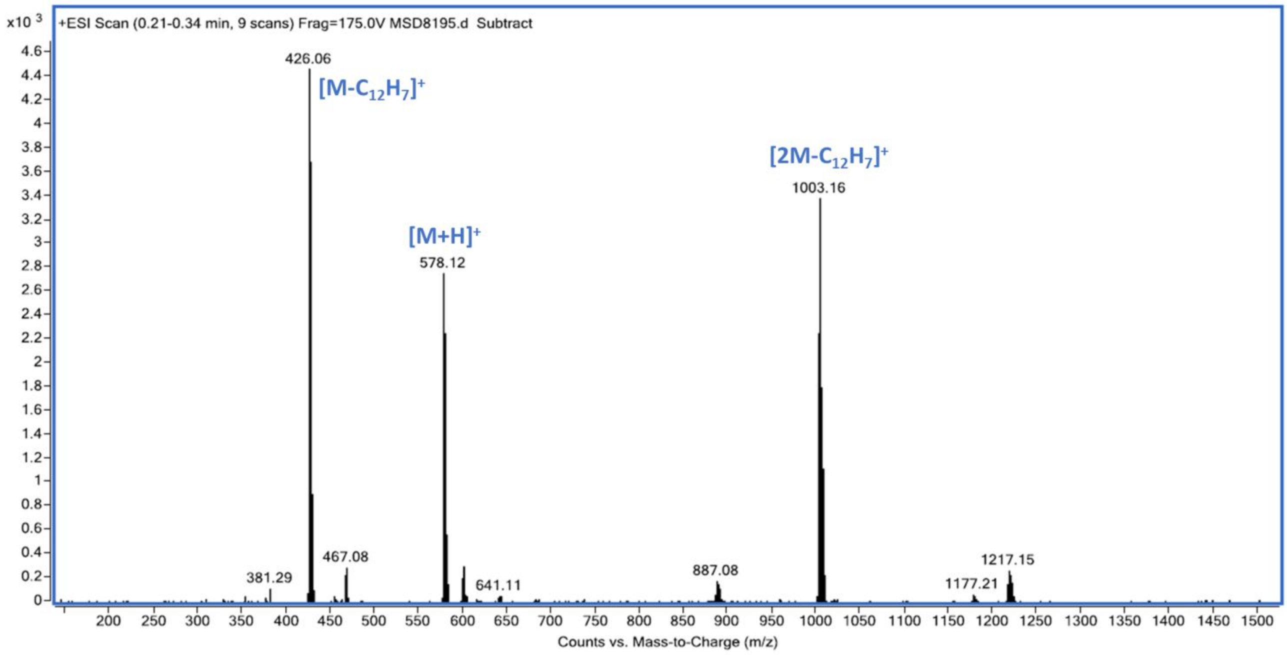

Figure S21. ESI-MS(+) spectrum of **1d**.

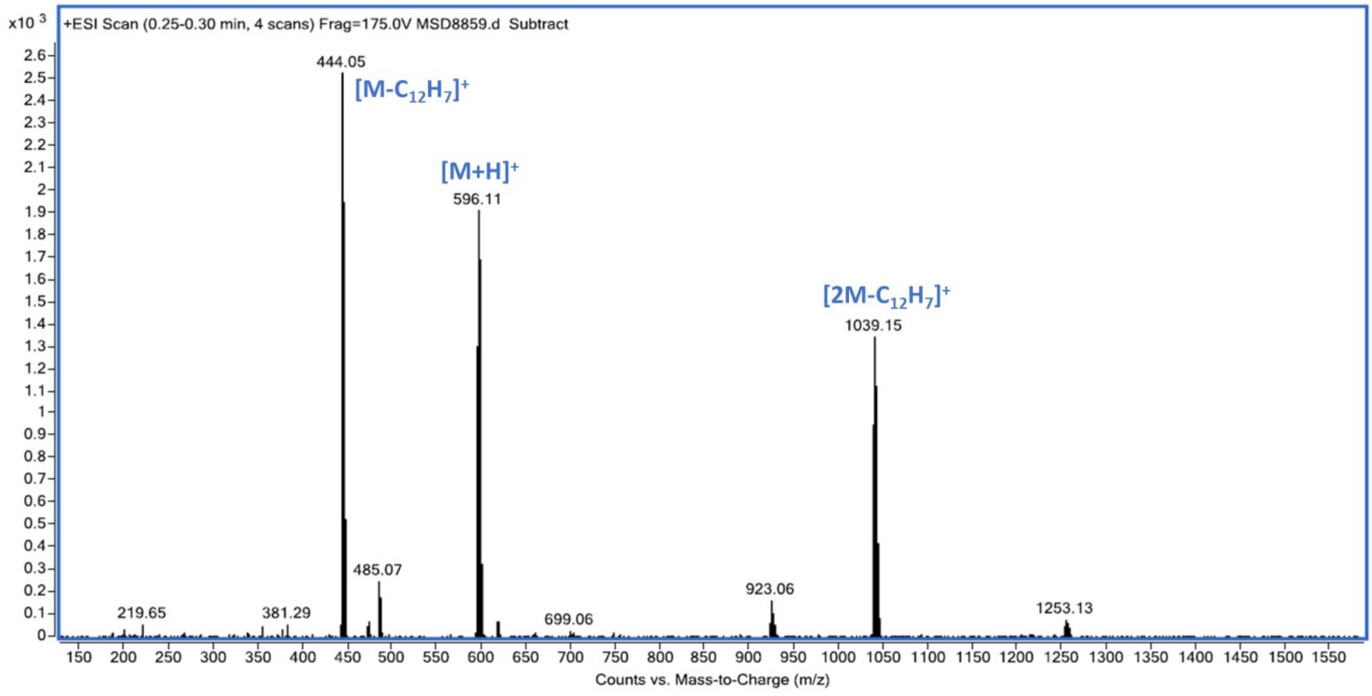

Figure S22. ESI-MS(+) spectrum of **2d**.

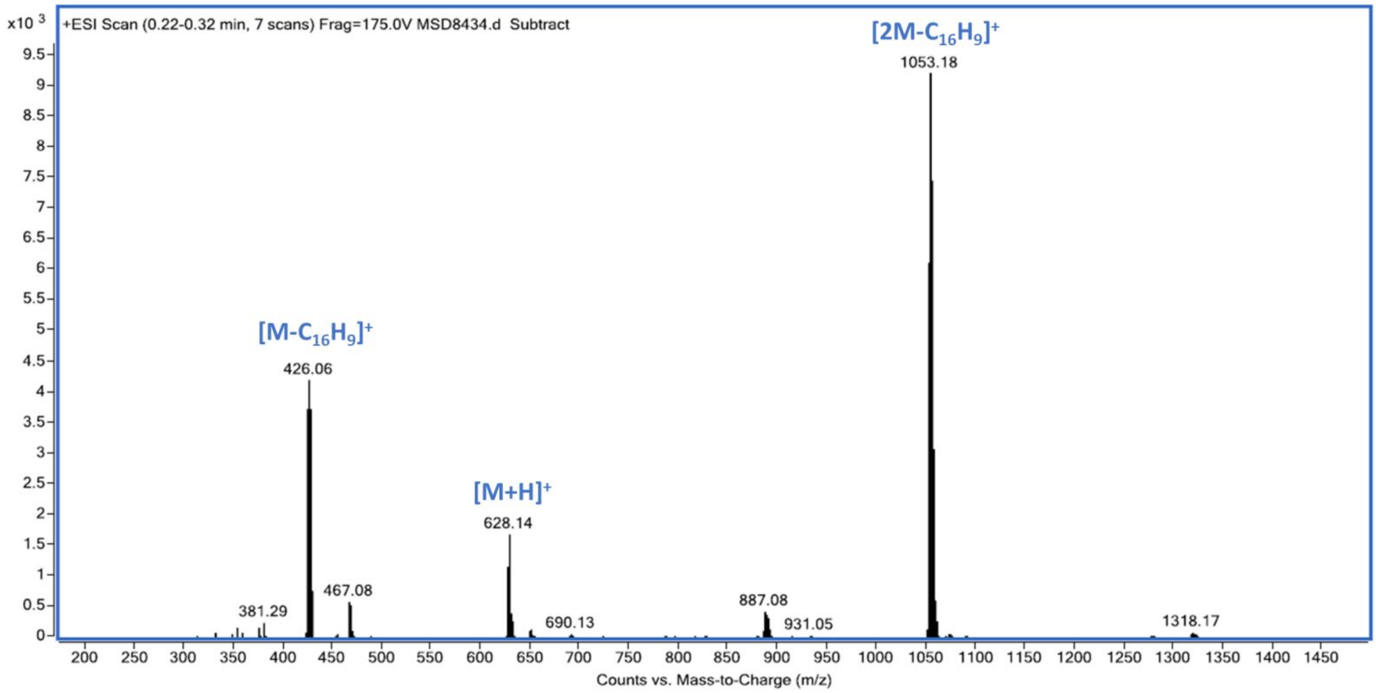

Figure S23. ESI-MS(+) spectrum of **1e**.

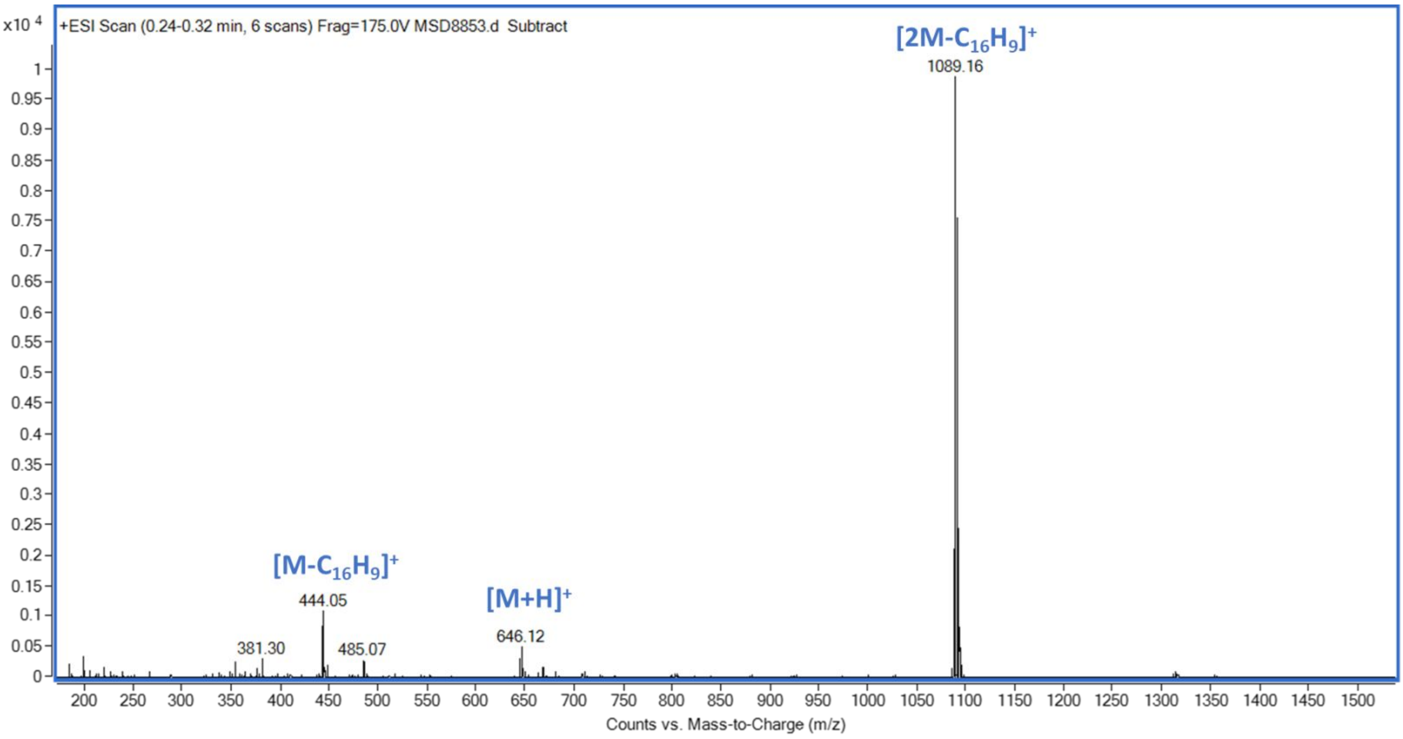

Figure S24. ESI-MS(+) spectrum of **2e**.

A)

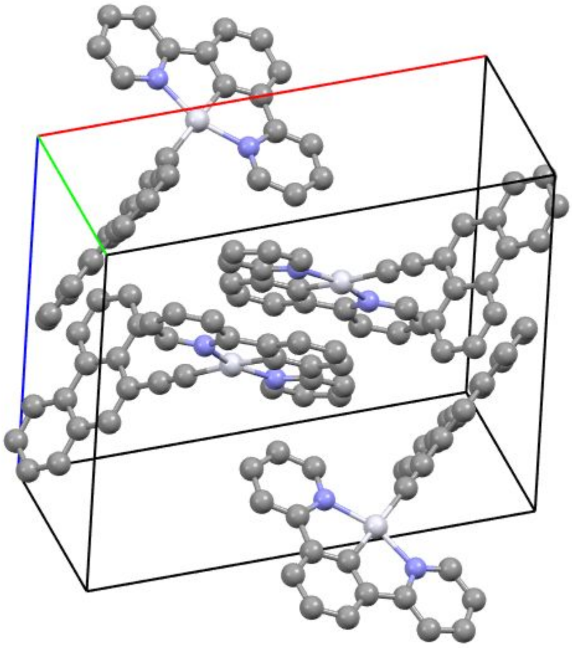

B)

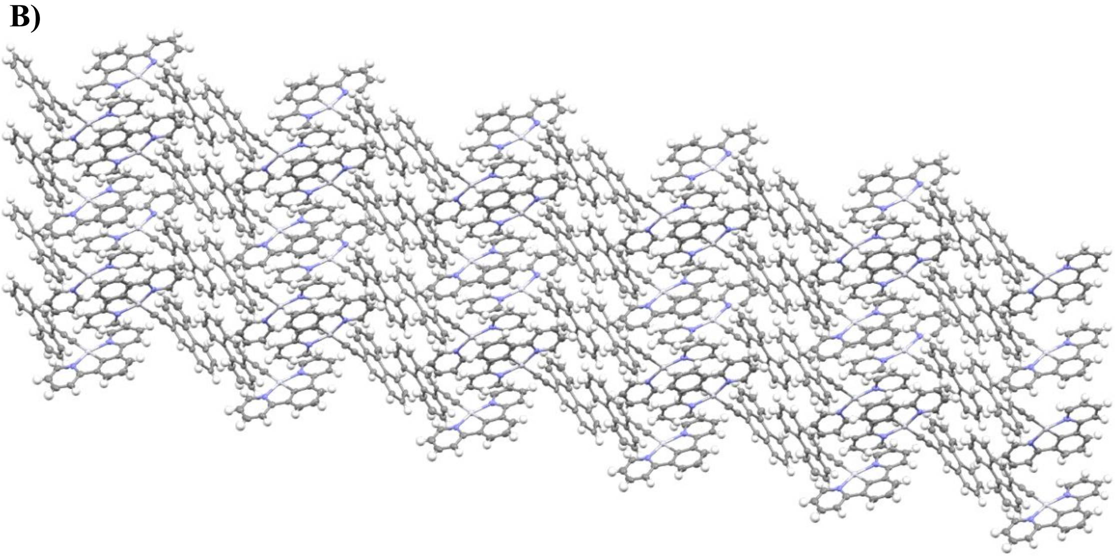

Figure S25 A) Unit cell of compound **1e**. **B)** A view of the packing of compound **1e**. Grey, platinum; blue, nitrogen.

A)

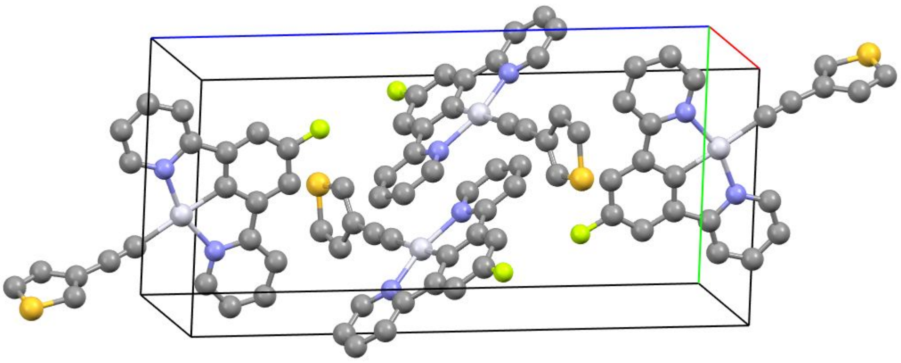

B)

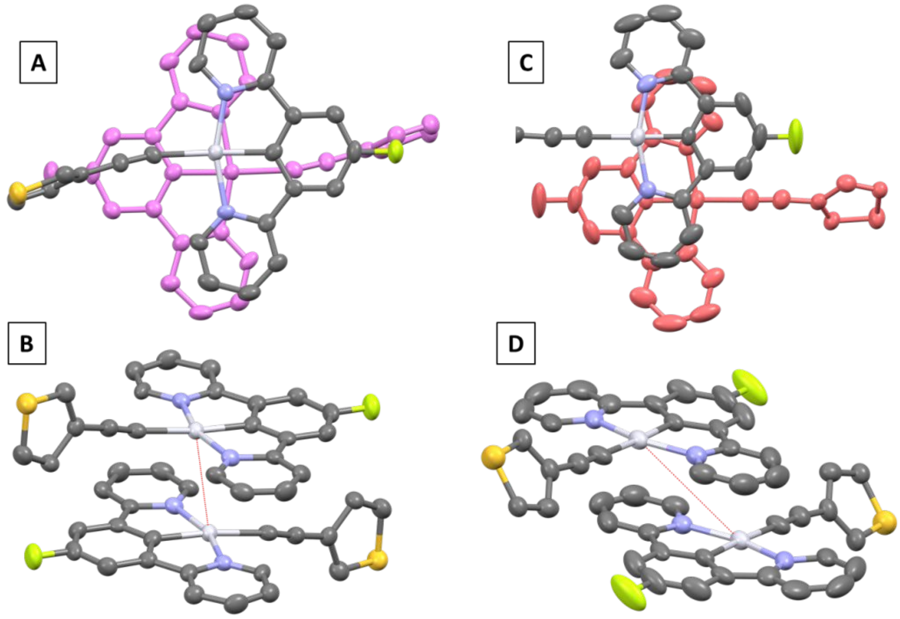

C)

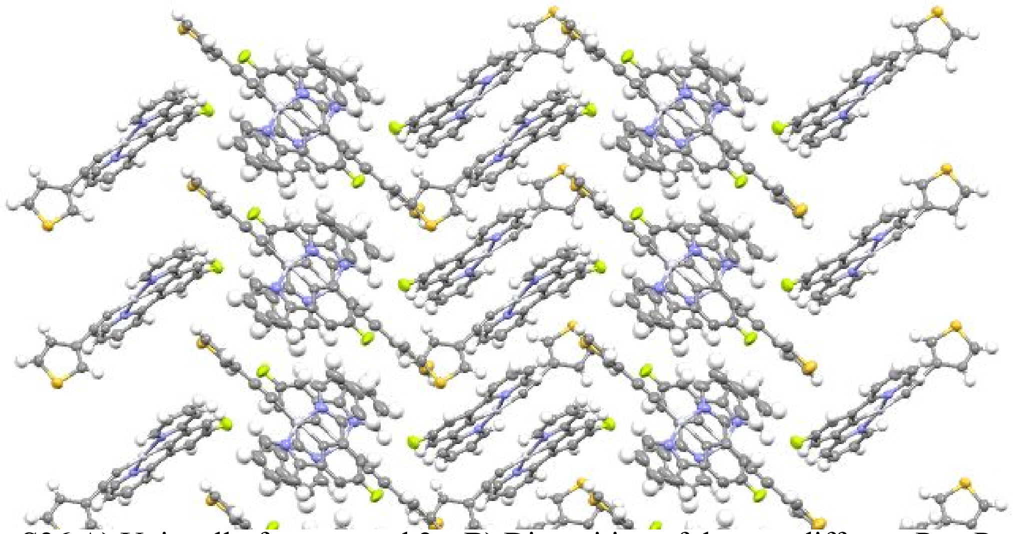

Figure S26 A) Unit cell of compound **2c**. **B)** Disposition of the two different Pt···Pt contacts for compound **2c**. **C)** A view of the packing of compound **2c** Grey, platinum; blue, nitrogen; yellow-green, fluorine; orange, sulfur.

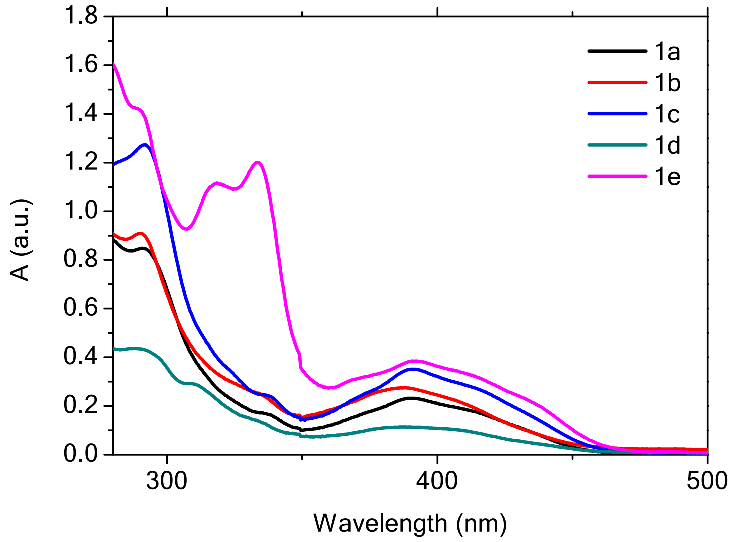

Figure S27. Absorption spectra for 5 x 10^{-5} M dichloromethane solutions of compounds **1**x.

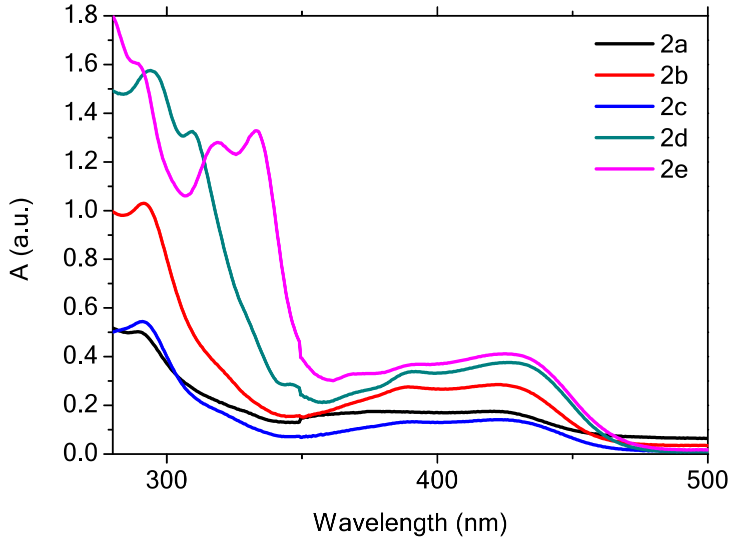

Figure S28. Absorption spectra for 5 x 10^{-5} M dichloromethane solutions of compounds **2x**.

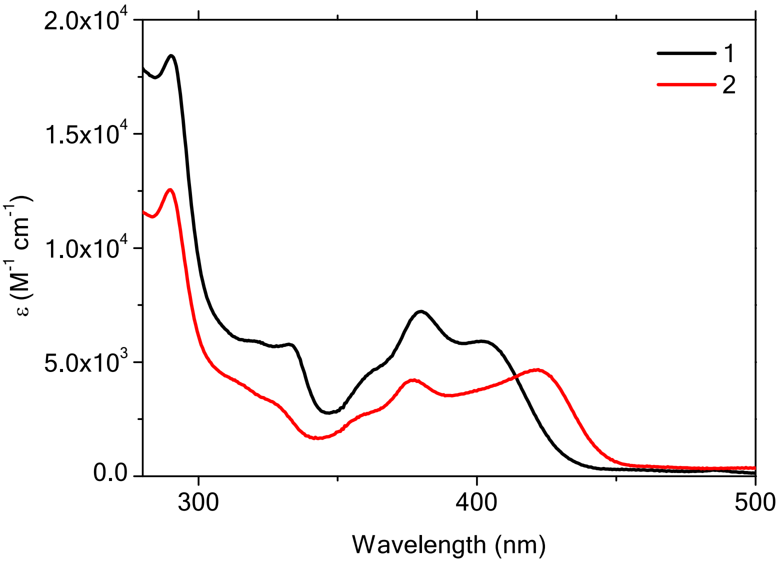

Figure S29. Absorption spectra for 5 x 10^{-5} M dichloromethane solutions of compounds **1** and **2** at 298 K.

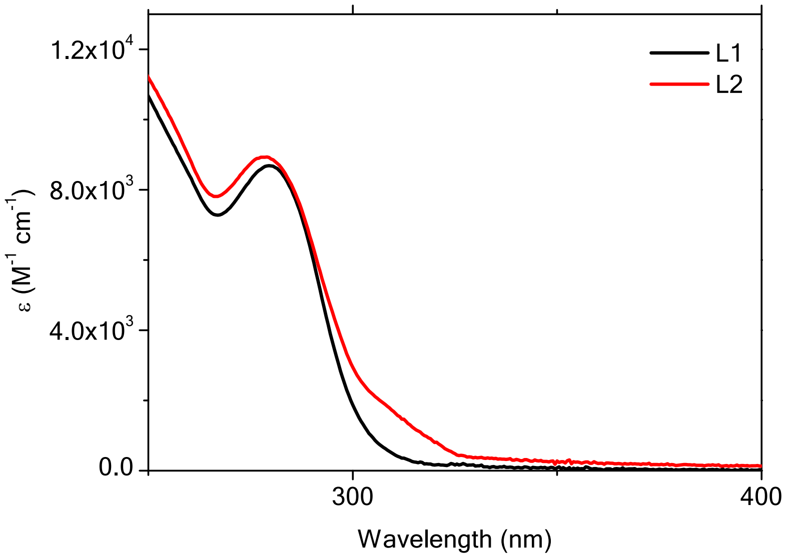

Figure S30. Absorption spectra for 5 x 10^{-5} M dichloromethane solutions of ligands **L1** and **L2** at 298 K.

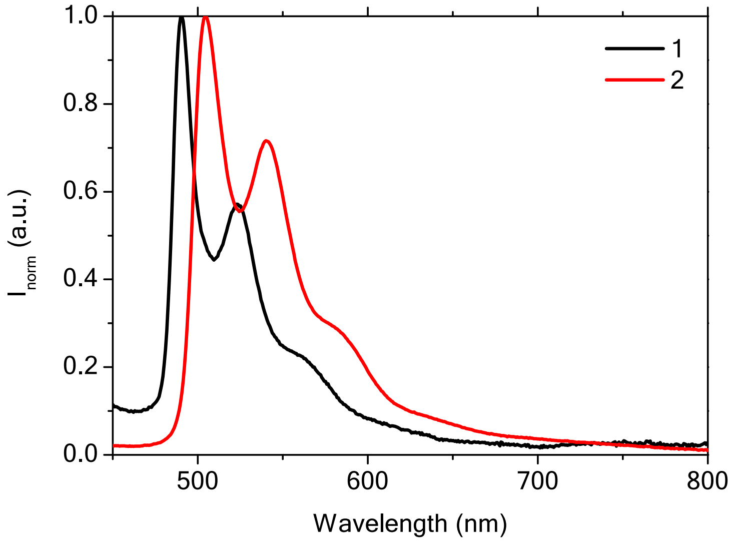

Figure S31. Normalized emission spectra for 5 x 10^{-5} M dichloromethane solutions of compounds **1** and **2** at 298 K (λ_{exc} = 380 nm).

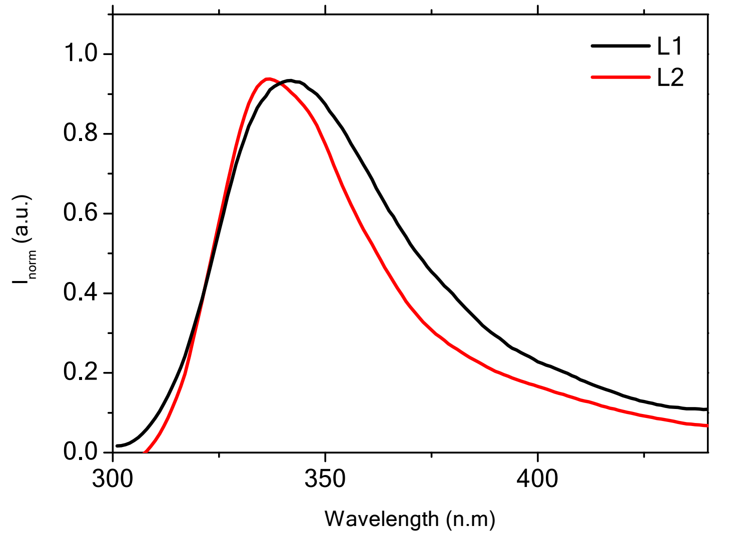

Figure S32. Normalized emission spectra for 5 x 10^{-5} M dichloromethane solutions of ligands **L1** and **L2** at 298 K (λ_{exc} = 280 nm).

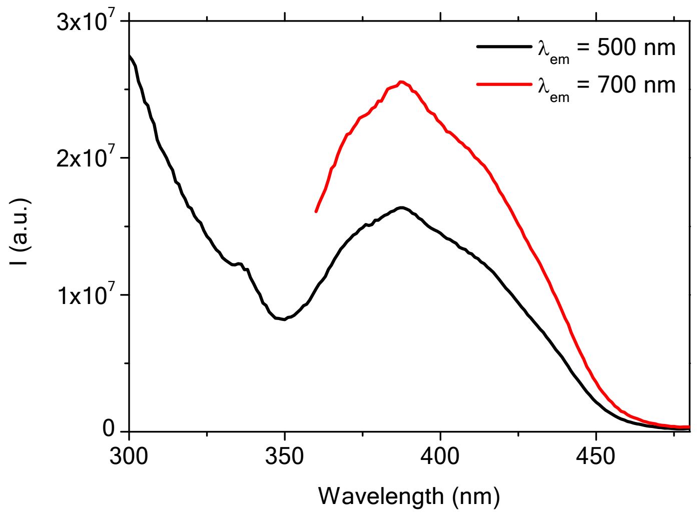

Figure S33. Excitation spectra of compound **2e** in a dichloromethane at the two different emission maxima at 298 K.

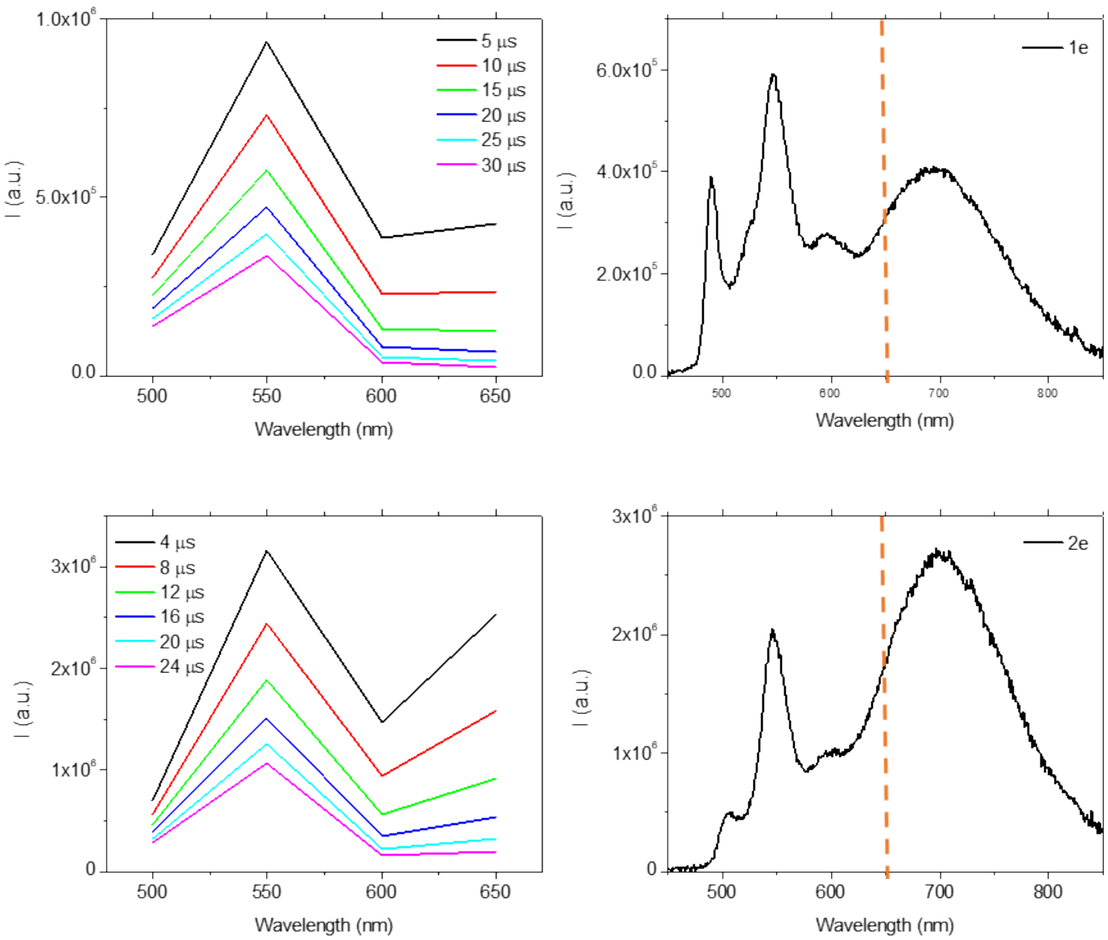

Figure S34. Time-resolved phosphorescence spectra (left) and emission spectra (right) in deareated dichloromethane solutions at 298 K of compounds **1e** and **2e**. Dotted lines indicates the end of region where the kinetics (left spectra) has been studied.

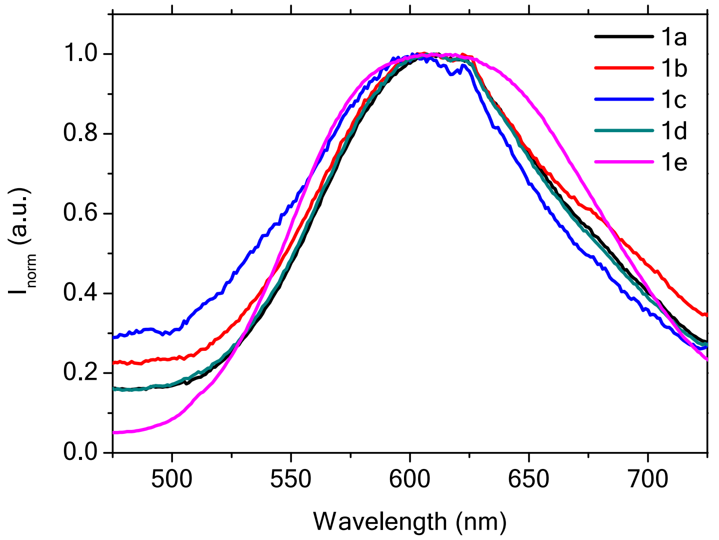

Figure S35. Normalized emission spectra in solid state of compounds **1a-e** at 298 K (λ_{exc} = 390 nm).

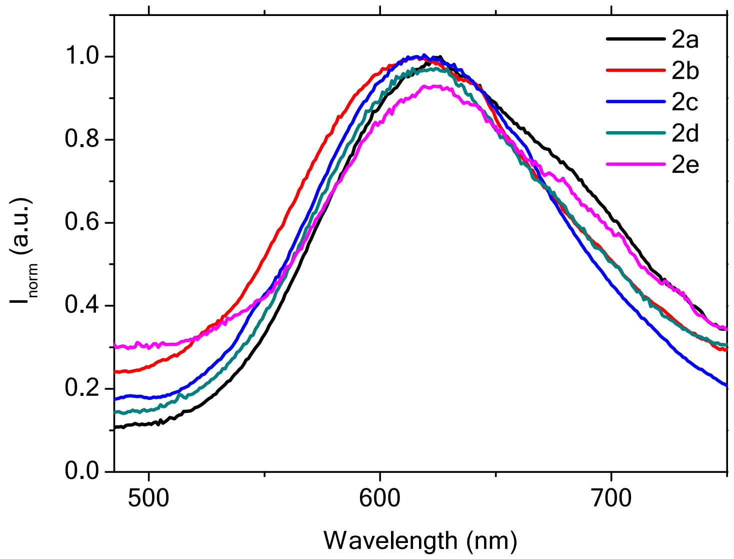

Figure S36. Normalized emission spectra in solid state of compounds **2a-e** at 298 K (λ_{exc} = 390 nm).

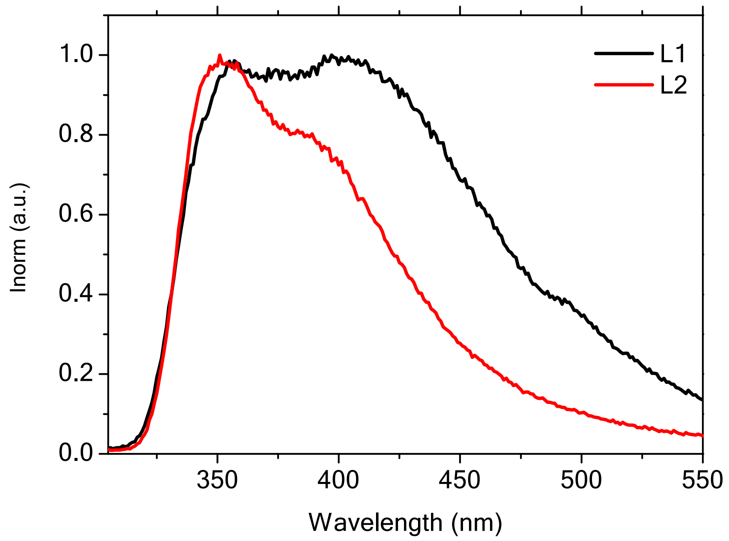

Figure S37. Normalized emission spectra in solid state of ligands **L1** and **L2** at 298 K (λ_{exc} = 280 nm).

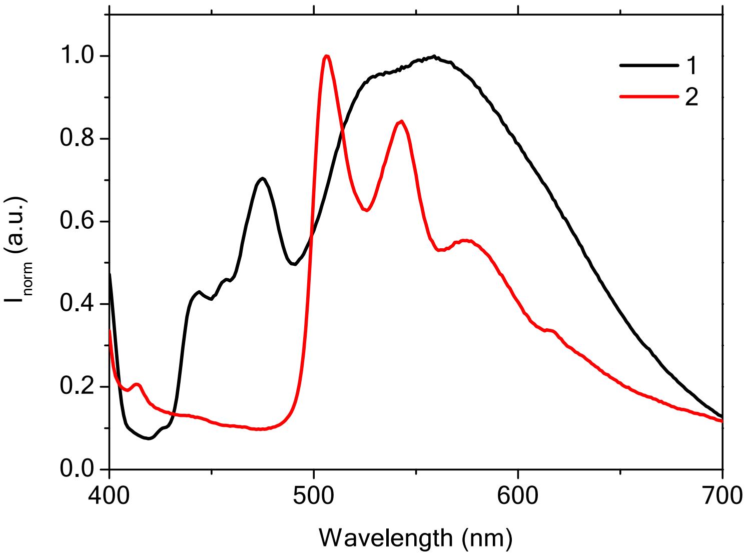

Figure S38. Normalized emission spectra in solid state of compounds **1** and **2** at 298 K (λ_{exc} = 380 nm).

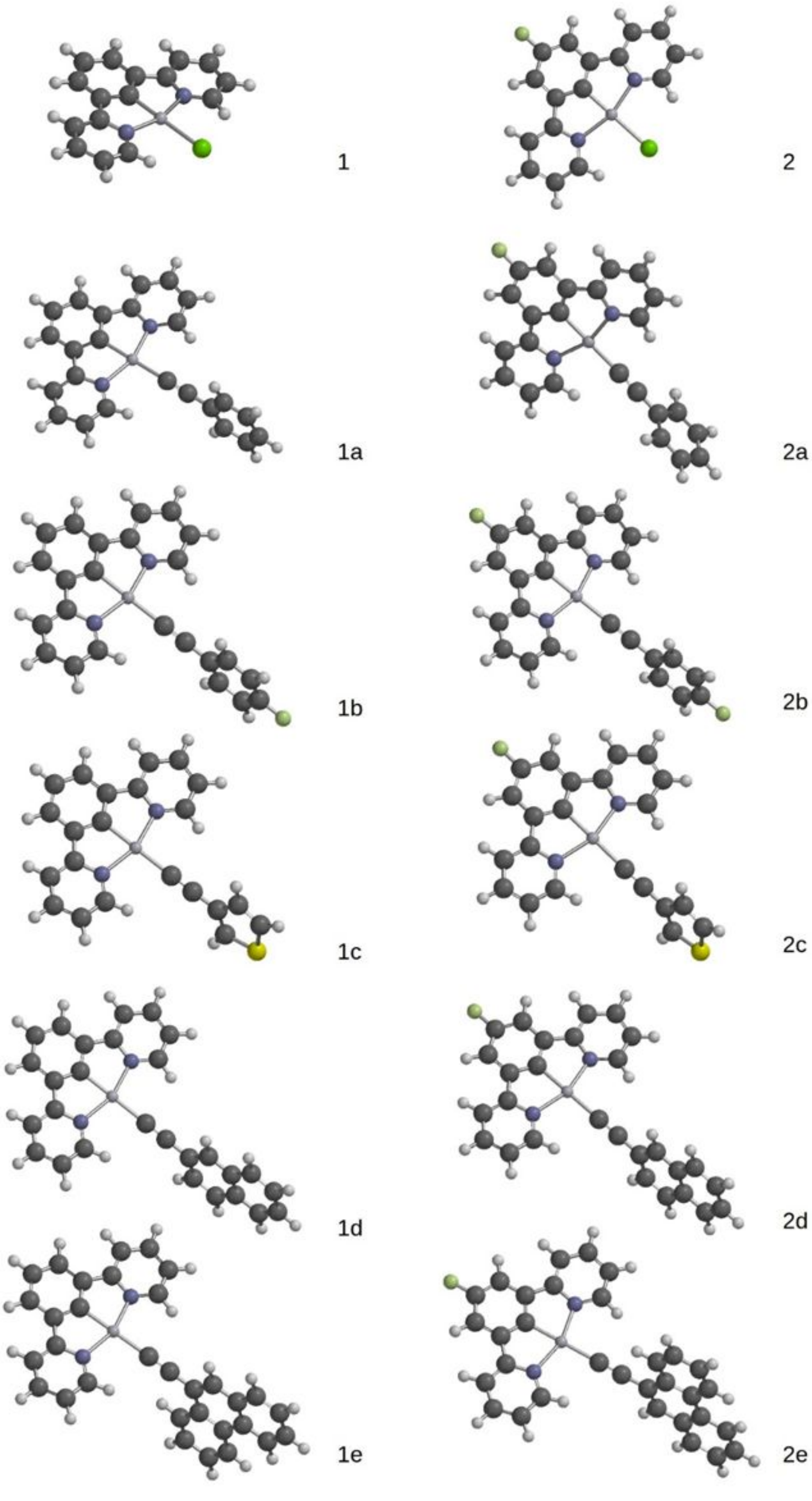

Figure S39. DFT optimized geometries in solution corresponding to the complexes **1**, **2**, **1a**-**1e** and **2a**-**2e**. Key geometrical parameters are listed in table S3.

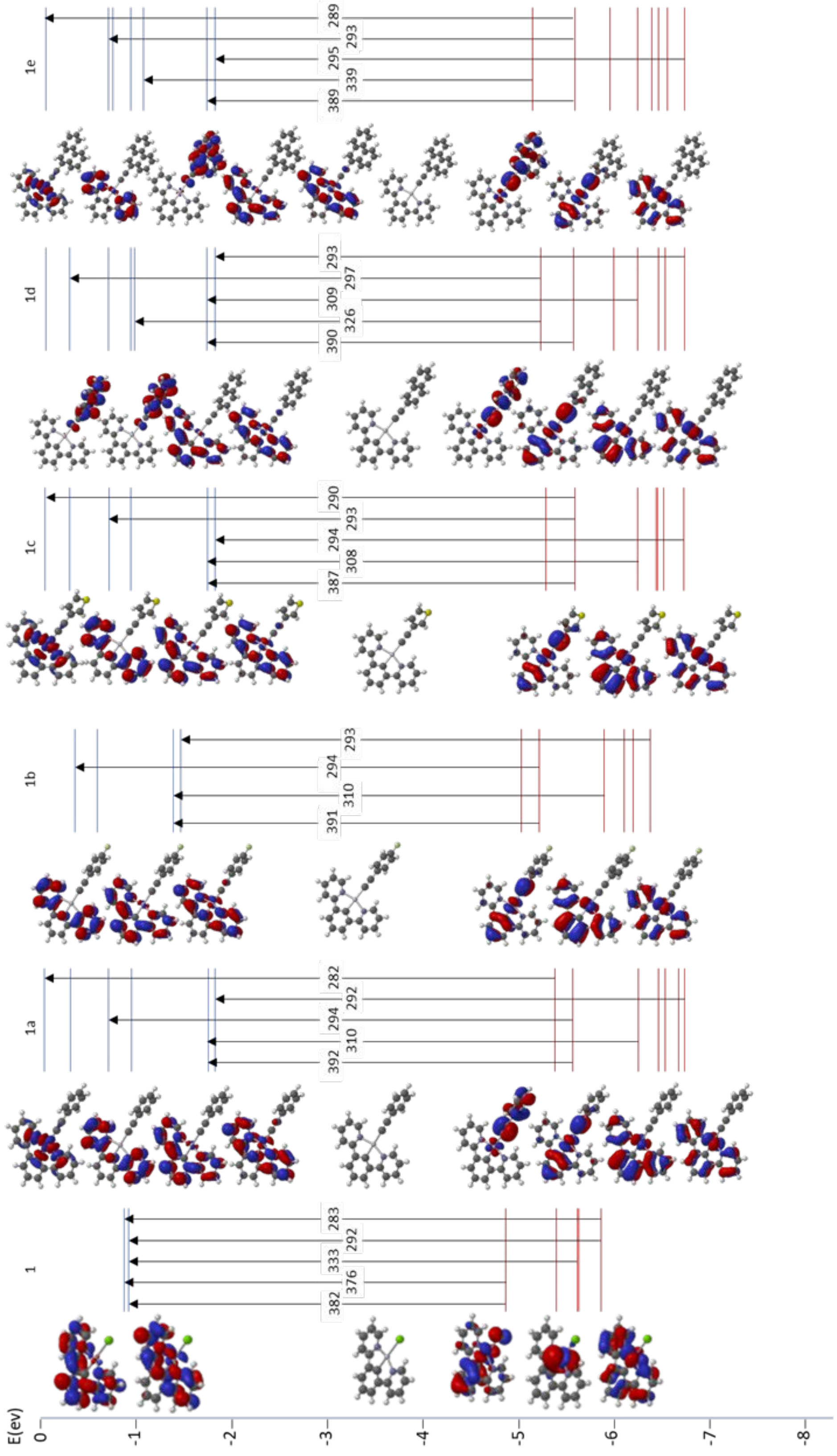

Figure S40a. Orbitals that participate in the UV-vis transitions discussed in the text for the complexes **1**, **1a**-**1e**. Red lines, occupied orbitals; blue lines, empty orbitals. The numbers in the arrows show the energy of thr transition in nm. The y-axis indicates the energy of the orbitals.

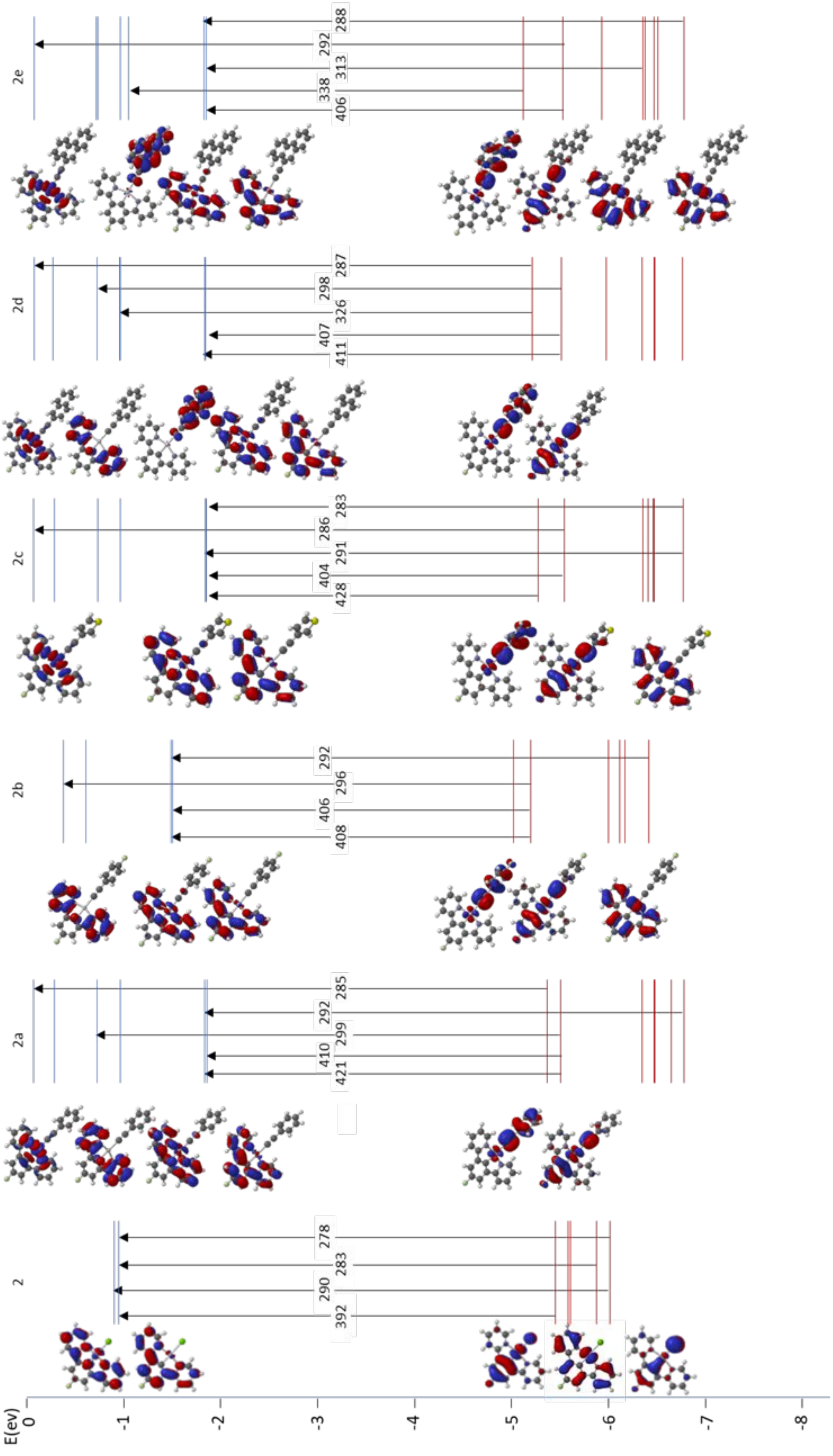

Figure S40b. Orbitals that participate in the UV-vis transitions discussed in the text for the complexes **2**,**2a**-**2e**. Red lines, occupied orbitals; blue lines, empty orbitals. The numbers in the arrows show the energy of thr transition in nm. The y-axis indicates the energy of the orbitals.

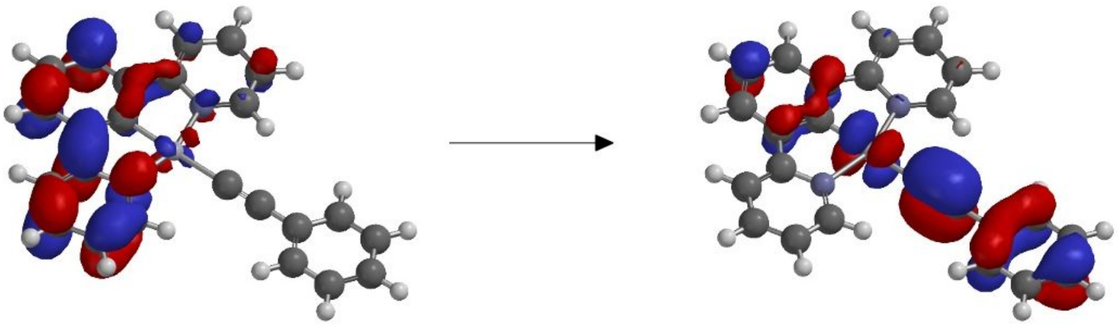

Figure S41. LUMO+1 and HOMO orbitals for complex **1a**.

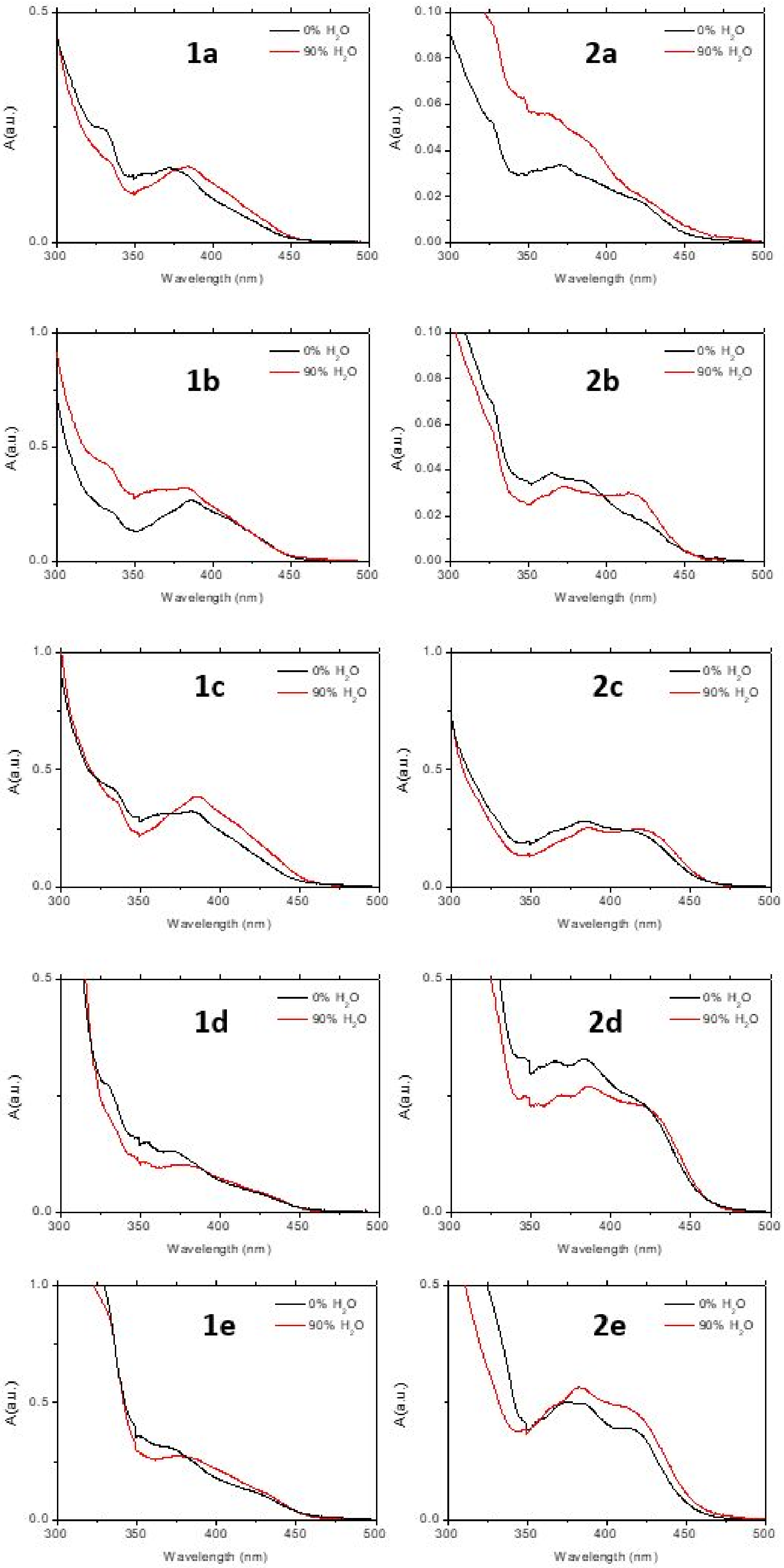

Figure S42. Absorption spectra for 5 x 10^{-5} M solutions for all compounds in acetonitrile and in water/acetonitrile (90:10) at 298 K.

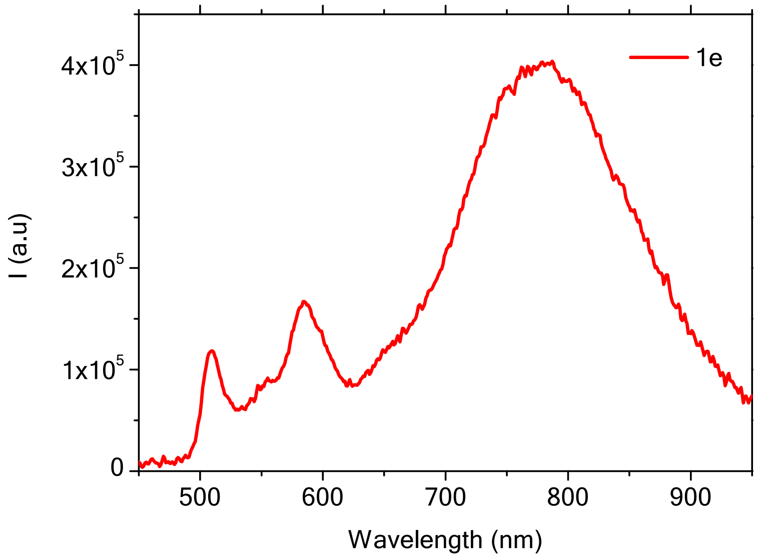

Figure S43. Emission spectra for a N_2-saturated acetonitrile solution of compound **1e** at 298 K (λ_{exc} = 390 nm).

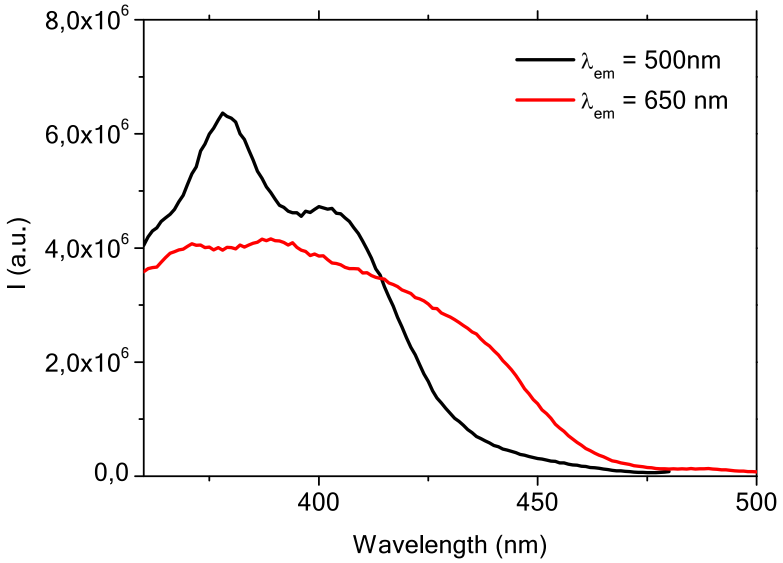

Figure S44. Excitation spectra of compound **2e** in a 90% water/10% acetonitrile at the two different emission maxima at 298 K.

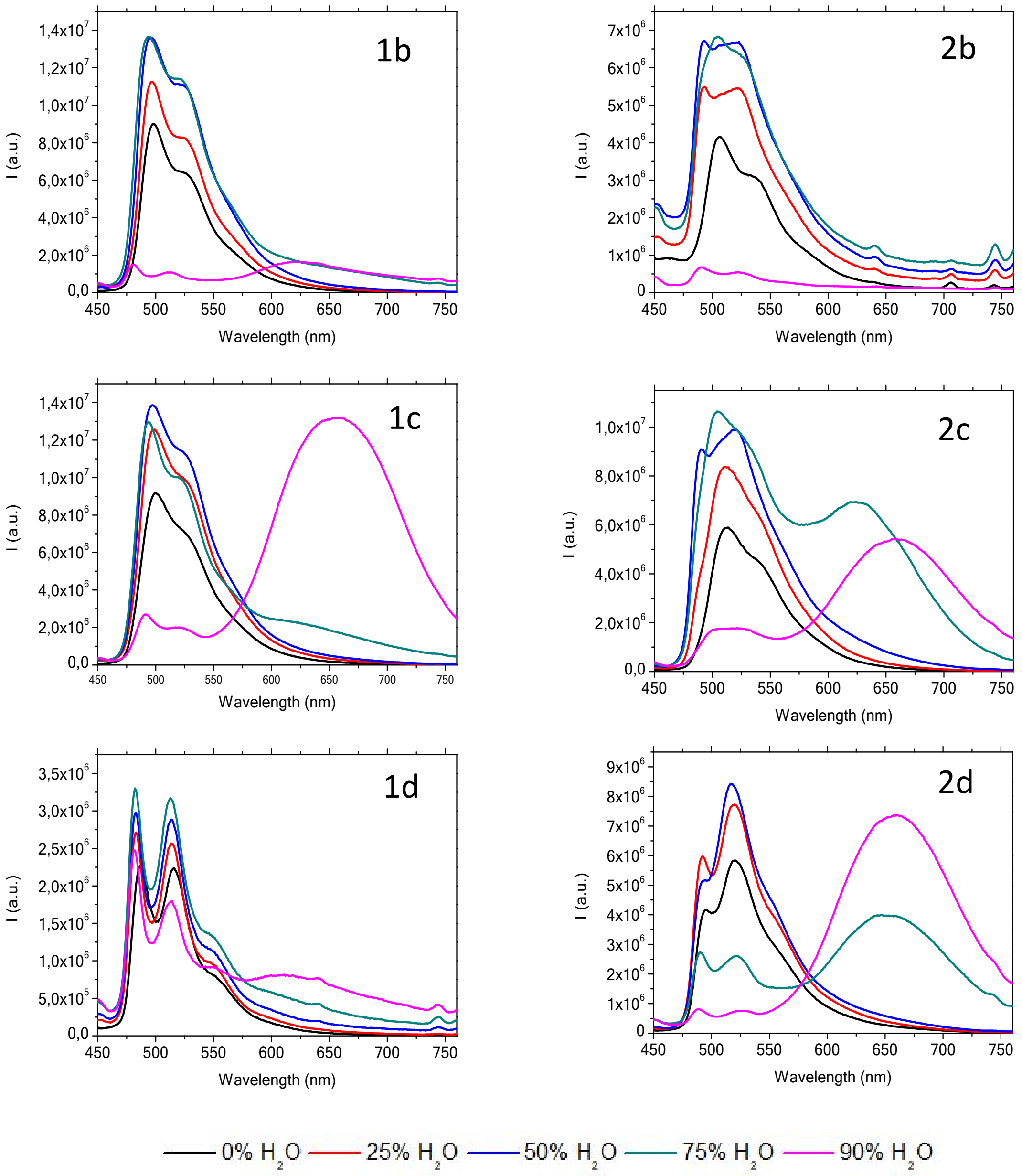

Figure S45. Emission spectra for acetonitrile/water mixtures of compounds **1b-d** and **2b-d** at 298 K (λ_{exc} = 390 nm).

Table S1. Crystal data and structure refinement for compounds **1e** and **2c**.

**Compound**	**1e**	**2c**
Formula	$C_{32}H_{20}N_2Pt$	$C_{22}H_{13}FN_2PtS$
Crystal size, mm	0.24 x 0.18 x 0.04	0.36 x 0.18 x 0.06
Crystal colour	yellow	orange
Fw	627.59	551.49
Temp, K	170	170
Wavelength, Å	0.71073	0.71073
Crystal system	Monoclinic	Triclinic
Space group	$P2_1/c$	$P\overline{1}$
a, Å	16.1728(7)	9.1838(3)
b, Å	11.3593(3)	9.5505(3)
c, Å	12.6711(6)	21.4829(8)
α, º	90	84.057(2)
β, º	99.073(2)	85.211(2)
γ, º	90	73.358(2)
Volume, $Å^3$	2298.70(16)	1793.94(11)
Z	4	4
D_{calc}, mg/m^3	1.813	2.042
abs. coef., mm^{-1}	6.129	7.957
F(000)	1216	1048
θ range for data coll, º	0.407 – 28.700	0.407 – 28.700
Reflns coll. / independent	17638 / 5083	17023 / 7859
Data /restraint /parameters	5683 / 0 / 316	7859 / 0 /487
GOF on F^2	1.047	1.066
Final R index (I>2σ(I))	R_1 = 0.0439,	R_1 = 0.0554,
	wR_2 = 0.1220	wR_2 = 0.0999
R index (all data)	R_1 = 0.0597,	R_1 = 0.0927,
	wR_2 = 0.1301	wR_2 = 0.1104
Peak and hole, e $Å^{-3}$	1.322 and -2.596	0.812 and -0.871
CCDC number	2192597	2192598

Table S2. Spectral data including radiative and non-radiative rate constants obtained in air-equilibrated (with O_2) or degassed (N_2 sat.) dichloromethane solutions.

**Compound**	k_r (μs^{-1}) **monomer (with O_2)**	k_{nr} (μs^{-1}) **monomer (with O_2)**	k_r (μs^{-1}) **monomer (N_2 sat.)**	k_{nr} (μs^{-1}) **monomer (N_2 sat.)**	k_r (μs^{-1}) **excimer (N_2 sat.)**	k_{nr} (μs^{-1}) **excimer (N_2 sat.)**
**L1**	1.023	254.731	-	-	-	-
**L2**	3.097	439.381	-	-	-	-
**1**	0.082	2.317	0.073	0.047	-	-
**2**	0.078	2.513	0.096	0.049	-	-
**1a**	0.123	3.041	0.087	0.104	0.051	0.290
**1b**	0.102	2.166	0.053	0.083	0.051	0.254
**1c**	0.143	4.341	0.078	0.141	0.054	0.281
**1d**	0.183	4.281	0.091	0.113	0.034	0.306
**1e**	0.032	2.621	0.031	0.210	0.040	0.150
**2a**	0.074	1.765	0.083	0.088	0.036	0.285
**2b**	0.067	2.331	0.049	0.095	0.050	0.283
**2c**	0.090	2.649	0.085	0.102	0.050	0.327
**2d**	0.060	2.153	0.038	0.100	0.063	0.209
**2e**	0.018	1.659	0.020	0.270	0.044	0.101

Table S3. Key geometrical parameters optimized at the DFT level in solution. C(1): metallated carbon of the pincer ligand. C(2): Alkyne carbon bonded to platinum. C(3): Alkyne carbon bonded to the aromatic moiety. C(4): aromatic carbon bonded to the alkyne moiety. Torsion: Angle between the aromatic moiety and the pincer ligand

**Parameter**	**1**	**1a**	**1b**	**1c**	**1d**	**1e**	**2**	**2a**	**2b**	**2c**	**2d**	**2e**
Pt-Cl	2.521	--	--	--	--	--	2.516	--	--	--	--	--
Pt-C(1)	1.930	1.962	1.961	1.962	1.963	1.963	1.930	1.961	1.962	1.962	1.963	1.963
Pt-N	2.076	2.080	2.080	2.080	2.081	2.082	2.075	2.079	2.080	2.081	2.081	2.082
Pt-C(2)	--	2.077	2.076	2.078	2.073	2.074	--	2.073	2.077	2.076	2.071	2.072
C(2)-C(3)	--	1.232	1.231	1.231	1.232	1.232	--	1.231	1.232	1.231	1.232	1.232
C(3)-C(4)	--	1.429	1.429	1.426	1.428	1.427	--	1.430	1.429	1.426	1.428	1.428
C(1)-Pt-N	80.4	79.5	79.5	79.5	79.5	79.5	80.4	79.5	79.5	79.4	79.5	79.4
C(1)-Pt-Cl	179.9	--	--	--	--	--	180.0	--	--	--	--	--
C(1)-Pt-C(2)	--	179.2	179.9	179.3	179.9	180.0	--	179.9	179.2	179.5	179.9	180.0
N-Pt-N	160.8	159.0	159.1	159.0	159.0	159.0	160.7	159.0	159.0	158.9	158.9	158.9
N-Pt-Cl	99.6	--	--	--	--	--	99.6	--	--	--	--	--
N-Pt-C(2)	--	100.5	100.5	100.5	100.5	100.5	--	100.5	100.5	100.6	100.6	100.6
Torsion	--	90.6	91.1	73.9	80.8	80.5	--	90.9	90.6	74.4	80.8	80.4

Table S4: Experimental and calculated UV-Vis transitions for the complexes studied in this work.

**Compound**	**λ_{exp}(nm)**	**log(ε)**	**λ_{calc}(nm)**	**f**	**Assignment**
**1**	402	4.9	381.7	0.0040	HOMO -> LUMO
	380	7.2	375.7	0.1514	HOMO -> LUMO+1
	333	5.8	331.1	0.0144	HOMO-2 -> LUMO
	290	18.4	307.9	0.0879	HOMO-1 -> LUMO+1
			292.3	0.3571	HOMO-4 -> LUMO
			283.1	0.1594	HOMO-4 -> LUMO+1
**1a**	389	4.6	392.6	0.1247	HOMO-1 -> LUMO+1
	291	17	310.2	0,1364	HOMO-2 -> LUMO+1
			294.4	0.1677	HOMO-1 -> LUMO+3
			292.8	0.3662	HOMO-6 -> LUMO
			283.2	0.0941	HOMO-6 -> LUMO+1
			282.5	0.1222	HOMO -> LUMO+5

**1b**	388	5.5	390.8	0.1271	HOMO-1 -> LUMO+1
	290	18.2	309.8<br>293.3<br>292.2<br>283.1<br>282.0	0.1376<br>0.1702<br>0.3481<br>0.0896<br>0.0870	HOMO-2 -> LUMO+1<br>HOMO-1 -> LUMO+3<br>HOMO-5 -> LUMO<br>HOMO-5 -> LUMO+1<br>HOMO -> LUMO+6
**1c**	391	7.0	387.3	0.1004	HOMO-1 -> LUMO+1
	292	25.5	308.6<br>294.3<br>292.7<br>290.3<br>283.3<br>282.8	0.1333<br>0.2223<br>0.1770<br>0.1877<br>0.0861<br>0.0850	HOMO-2 -> LUMO+1<br>HOMO-6 -> LUMO<br>HOMO-1 -> LUMO+3<br>HOMO-1 -> LUMO+5<br>HOMO-6 -> LUMO+1<br>HOMO-1 -> LUMO+5
**1d**	389	2.3	390.2	0.1186	HOMO-1 -> LUMO+1
	309	5.8	326.1<br>309.1	0.3105<br>0.1357	HOMO -> LUMO+2<br>HOMO-3 -> LUMO+1
	288	8.7	297.4<br>293.3<br>293.2<br>289.6<br>284.6	0.2723<br>0.1051<br>0.2574<br>0.0999<br>0.1266	HOMO -> LUMO+5<br>HOMO-1 -> LUMO+4<br>HOMO-6 -> LUMO<br>HOMO-1 -> LUMO+6<br>HOMO -> LUMO+6
**1e**	391	7.7	388.9	0.1234	HOMO-1 -> LUMO+1
	334	24	338.6	0.5357	HOMO -> LUMO+2
	319	22.6	309.8<br>308.6	0.0546<br>0.0462	HOMO-4 -> LUMO<br>HOMO-3 -> LUMO+1
	289	28.4	295.8<br>292.6<br>289.8	0.1243<br>0.1417<br>0.214	HOMO-7 -> LUMO<br>HOMO-1 -> LUMO+5<br>HOMO-1 -> LUMO+6

			285.8	0.1236	HOMO-1 -> LUMO+6
			285.4	0.1135	HOMO-2 -> LUMO+2
**2**	422	4.7	392.1	0.1627	HOMO -> LUMO
	290	12.5	290.4	0.3026	HOMO-4 -> LUMO+1
			283.2	0.1820	HOMO-4 -> LUMO
			278.1	0.1346	HOMO-5 -> LUMO
**2a**	421	3.5	412.3	0.0476	HOMO-1 -> LUMO+1
	380	3.5	409.8	0.1329	HOMO-1 -> LUMO
	289	10.5	298.8	0.1417	HOMO-1 -> LUMO+3
			291.9	0.3011	HOMO-6 -> LUMO+1
			284.6	0.1729	HOMO -> LUMO+5
			283.2	0.1359	HOMO-6 -> LUMO
**2b**	422	5.7	408.0	0.0477	HOMO-1 -> LUMO+1
	390	5.5	405.6	0.1321	HOMO-1 -> LUMO
	289	10.1	314.4	0.1055	HOMO-2 -> LUMO
			296.2	0.1357	HOMO-1 -> LUMO+3
			292.0	0.3288	HOMO-5 -> LUMO+1
			284.0	0.1573	HOMO -> LUMO+5
			283.4	0.1315	HOMO-5 -> LUMO
**2c**	424	2.8	428.4	0.0284	HOMO -> LUMO
	391	2.7	408.6	0.0337	HOMO-1 -> LUMO+1
			403.9	0.1059	HOMO-1 -> LUMO
	291	10.8	291.5	0.3000	HOMO-6 -> LUMO+1
			285.8	0.1577	HOMO-1 -> LUMO+5
			283.4	0.1267	HOMO-6 -> LUMO
**2d**	427	7.5	410.5	0.0592	HOMO-1 -> LUMO+1
	392	6.8	407.1	0.1251	HOMO-1 -> LUMO
	310	2.5	325.7	0.3406	HOMO -> LUMO+3

	294	31.5	298.2<br>291.7<br>286.8<br>283.2	0.3064<br>0.2216<br>0.2347<br>0.1238	HOMO-1 -> LUMO+4<br>HOMO-6 -> LUMO+1<br>HOMO -> LUMO+6<br>HOMO-6 -> LUMO
**2e**			409.6	0.0678	HOMO-1 -> LUMO+1
	425	8.2	405.8	0.1314	HOMO-1 -> LUMO
	333	26.6	338.1	0.5482	HOMO -> LUMO+2
	319	25.6	313.4	0.0633	HOMO-3 -> LUMO
			296.7	0.1105	HOMO-1 -> LUMO+5
			291.9	0.1426	HOMO-1 -> LUMO+6
	290	32.1	288.2<br>285.7<br>283.5<br>283.1	0.3220<br>0.0851<br>0.0639<br>0.1184	HOMO-7 -> LUMO+1<br>HOMO-2 -> LUMO+2<br>HOMO-1 -> LUMO+4<br>HOMO-7 -> LUMO

Table S5: Experimental and calculated emission energies.

**Compound**	**λ_{em}(exp, nm)**	**λ_{em}(calc, nm)**	**Assignment**
**1**	490	440.1	HOMO-1 -> LUMO
**1a**	497	485.6	HOMO -> LUMO+1
**1b**	495	486.8	HOMO -> LUMO+1
**1c**	501	494.9	HOMO -> LUMO+1
**1d**	490	485.3	HOMO -> LUMO+1
**1e**	547	514.4	HOMO -> LUMO (43%) // HOMO -> LUMO+2 (38%)
**2**	505	521.6	HOMO -> LUMO+1
**2a**	507	503.6	HOMO -> LUMO+1
**2b**	511	505.6	HOMO -> LUMO+1

**2c**	513	512.9	HOMO -> LUMO+1
**2d**	520	518.0	HOMO -> LUMO+1
**2e**	547	527.9	HOMO -> LUMO+1

Table S6. Phosphorescence quantum yields and lifetimes recorded in aerated acetonitrile/water mixtures for compounds **1a-e** and **2a-e**.

**Compound**	**Water percentage (%)**	ϕ_{Ph}	ϕ_{Ph} **monomer**	ϕ_{Ph} **aggregates**	**τ (μs) monomer**	**τ (μs) aggregates**
**1a**	0	0.01	0.01	-	0.19	0.06
	25	0.02	0.02	-	0.37	0.09
	50	0.02	0.02	-	0.48	0.01
	75	0.03	0.01	0.02	0.85	0.27
	90	0.04	0.01	0.03	1.34	0.13
**1b**	0	0.01	0.01	-	0.25	0.08
	25	0.01	0.01	-	0.37	0.10
	50	0.02	0.02	-	0.47	0.15
	75	0.03	0.02	0.02	0.87	0.23
	90	0.04	0.01	0.03	1.25	0.10
**1c**	0	0.01	0.01	-	0.23	0.05
	25	0.01	0.01	-	0.35	0.07
	50	0.02	0.02	-	0.52	0.01
	75	0.04	0.01	0.02	0.86	0.25
	90	0.12	0.01	0.12	1.25	0.23
**1d**	0	0.01	0.01	-	0.26	0.11

	25	0.01	0.01	-	0.37	0.02
	50	0.02	0.02	-	0.50	0.10
	75	0.02	0.01	0.01	0.95	0.19
	90	0.03	0.01	0.02	1.17	0.09
**1e**	0	0.01	0.01	-	0.22	0.08
	25	0.01	0.01	-	0.36	0.16
	50	0.02	0.02	-	0.44	0.21
	75	0.07	0.01	0.06	0.74	0.20
	90	0.08	0.01	0.07	0.79	0.18
**2a**	0	0.01	0.01	-	0.26	0.12
	25	0.01	0.01	-	0.36	0.18
	50	0.02	0.02	-	0.48	0.24
	75	0.03	0.03	-	0.76	0.11
	90	0.01	0.01	-	0.91	0.177
**2b**	0	0.01	0.01	-	0.24	0.10
	25	0.02	0.02	-	0.36	0.09
	50	0.02	0.02	-	0.47	0.13
	75	0.03	0.03	-	0.78	0.23
	90	0.01	0.01	-	0.77	0.09
**2c**	0	0.01	0.01	-	0.21	0.08
	25	0.01	0.01	-	0.22	0.08
	50	0.02	0.02	-	0.43	0.11
	75	0.04	0.02	0.02	0.81	0.14
	90	0.06	0.01	0.05	1.18	0.24
**2d**	0	0.01	0.01	-	0.22	0.08
	25	0.01	0.01	-	0.34	0.07

	50	0.01	0.01	-	0.44	0.03
	75	0.02	0.01	0.02	0.78	0.26
	90	0.07	0.01	0.07	0.78	0.11
**2e**	0	0.01	0.01	-	0.27	0.12
	25	0.01	0.01	-	0.35	0.20
	50	0.02	0.02	-	0.46	0.25
	75	0.06	0.01	0.05	0.77	0.18
	90	0.11	-	0.11	0.73	0.14

Table S7. Spectral data including radiative and non-radiative rate constants obtained in aerated Acetonitrile/Water mixtures for compounds **1a-e**.

**Compound**	Acetonitrile percentage (%)	k_r (**μs^{-1}**) **monomer (with O_2)**	k_{nr} (**μs^{-1}**) **monomer (with O_2)**	k_r (**μs^{-1}**) **Aggregates (with O_2)**	k_{nr} (**μs^{-1}**) **Aggregates (with O_2)**
**1a**	100	0.052	5.183	-	-
	75	0.041	2.669	-	-
	50	0.042	2.063	-	-
	25	0.015	1.163	0.064	3.709
	10	0.005	0.740	0.233	7.519
**1b**	100	0.048	3.968	-	-
	75	0.038	2.694	-	-
	50	0.049	2.083	-	-
	25	0.018	1.132	0.066	4.301
	10	0.005	0.794	0.316	9.888
**1c**	100	0.035	4.370	-	-

	75	0.037	2.804	-	-
	50	0.035	1.885	-	-
	25	0.015	1.142	0.090	3.992
	10	0.002	0.798	0.513	3.835
**1d**	100	0.023	3.794	-	-
	75	0.030	2.702	-	-
	50	0.030	1.982	-	-
	25	0.014	1.038	0.043	5.333
	10	0.007	0.849	0.193	11.170
**1e**	100	0.032	4.555	-	-
	75	0.037	2.780	-	-
	50	0.034	2.259	-	-
	25	0.003	1.341	0.312	4.639
	10	0.005	1.256	0.415	5.267

Table S8. Spectral data including radiative and non-radiative rate constants obtained in aerated Acetonitrile/Water mixtures for compounds **2a-e**.

**Compound**	Acetonitrile percentage (%)	k_r **(μs^{-1})** **monomer (with O_2)**	k_{nr} **(μs^{-1})** **monomer (with O_2)**	k_r **(μs^{-1})** **Aggregates (with O_2)**	k_{nr} **(μs^{-1})** **Aggregates (with O_2)**
**2a**	100	0.039	3.852	-	-
	75	0.036	2.719	-	-
	50	0.036	2.056	-	-
	25	0.033	1.288	-	-
	10	0.013	1.089	-	-

**2b**	100	0.053	4.062	-	-
	75	0.050	2.743	-	-
	50	0.044	2.070	-	-
	25	0.037	1.242	-	-
	10	0.018	1.289	-	-
**2c**	100	0.038	4.657	-	-
	75	0.064	4.523	-	-
	50	0.049	2.266	-	-
	25	0.022	1.209	0.168	6.825
	10	0.005	0.842	0.220	4.017
**2d**	100	0.040	4.444	-	-
	75	0.032	2.900	-	-
	50	0.032	2.241	-	-
	25	0.006	1.277	0.067	3.855
	10	0.003	1.288	0.655	8.436
**2e**	100	0.015	3.675	-	-
	75	0.031	2.794	-	-
	50	0.050	2.119	-	-
	25	0.009	1.298	0.291	5.203
	10	-	-	0.739	6.303

Table S9. Optimized geometries

Optimized geometries (ground state)

1

```
31
Molecule 1
Pt       0.001170      0.002450     -1.434105
C       -0.000664     -0.002268      0.495756
C       -0.000074     -0.007305      3.247911
C        1.230610     -0.003185      1.163125
C       -1.231315     -0.001651      1.162476
C       -1.224652     -0.005963      2.565836
C        1.223190     -0.005153      2.566493
H       -2.151303     -0.006948      3.133772
H        2.149522     -0.006726      3.134946
H       -0.000078     -0.009377      4.333771
N       -2.046225      0.000118     -1.088945
C       -4.721833      0.000163     -0.351386
C       -2.383112      0.000061      0.248715
C       -3.008828      0.004687     -2.026675
C       -4.360103      0.004553     -1.698298
C       -3.726961      0.000073      0.623749
H       -2.661704      0.007189     -3.053525
H       -5.103161      0.006843     -2.488053
H       -3.986922     -0.003547      1.676834
H       -5.767847      0.000166     -0.060064
N        2.046790      0.005019     -1.087823
C        4.721984      0.001565     -0.348813
C        2.382935     -0.000896      0.250000
C        3.009896      0.004526     -2.025041
C        4.360991      0.003149     -1.695930
C        3.726580     -0.002629      0.625769
H        2.663335      0.006586     -3.052084
H        5.104481      0.004321     -2.485282
H        3.985963     -0.003689      1.679002
H        5.767839      0.000232     -0.056923
Cl      -0.000505      0.007636     -3.955212
```

1a

```
43
Molecule 1a
Pt      -0.052739     -0.003300     -0.639802
C       -0.072897      0.000550     -2.601695
C       -0.101324      0.005839     -5.361463
C       -0.077198     -1.225323     -3.278731
C       -0.076579      1.228989     -3.274026
C       -0.092448      1.229345     -4.677238
C       -0.093058     -1.220294     -4.681930
H       -0.097161      2.155899     -5.246067
H       -0.098223     -2.144637     -5.254344
H       -0.113618      0.007929     -6.447465
N       -0.040884      2.042225     -1.014051
C       -0.043928      4.719182     -1.752561
C       -0.059393      2.378172     -2.354240
C       -0.019710      3.007810     -0.077612
C       -0.022194      4.358637     -0.405179
C       -0.061404      3.723915     -2.726234
H       -0.003875      2.658207      0.948180
H       -0.008502      5.102265      0.384066
H       -0.077774      3.984407     -3.779197
H       -0.047265      5.765028     -2.044712
N       -0.041607     -2.047113     -1.021925
C       -0.046582     -4.721222     -1.770238
C       -0.060588     -2.378034     -2.363341
C       -0.020963     -3.016057     -0.088964
C       -0.024403     -4.365657     -0.421509
C       -0.063553     -3.722395     -2.740281
H       -0.004725     -2.669982      0.938043
H       -0.011092     -5.112217      0.364970
H       -0.080315     -3.978939     -3.794201
H       -0.050667     -5.765980     -2.066246
C       -0.000475     -0.006748      1.436318
C        0.044172     -0.006433      2.667046
C        0.092731     -0.003918      4.094949
C        0.186388      0.005673      6.918261
C       -1.093359     -0.023247      4.861061
C        1.327145      0.019540      4.780527
C        1.369957      0.024094      6.173589
C       -1.043715     -0.018269      6.253718
H       -2.051480     -0.042089      4.349084
H        2.249220      0.034346      4.205964
H        2.331775      0.041868      6.680147
H       -1.969839     -0.032037      6.822886
H        0.222149      0.009973      8.004444
```

1b

```
43
Molecule 1b
Pt      -0.021152     -0.002019     -0.646376
C       -0.059596      0.000917     -2.607240
C       -0.117192      0.005046     -5.366318
C       -0.074115     -1.225286     -3.283521
C       -0.073207      1.229134     -3.279860
C       -0.102610      1.229095     -4.682653
C       -0.103500     -1.221051     -4.686308
H       -0.114800      2.155441     -5.251658
H       -0.116354     -2.145672     -5.258102
H       -0.140181      0.006676     -6.452184
N       -0.029085      2.043493     -1.020930
C       -0.043909      4.720369     -1.761895
C       -0.055168      2.378660     -2.360860
C       -0.011625      3.009881     -0.085822
C       -0.018203      4.360587     -0.414447
C       -0.062496      3.723906     -2.734415
H        0.008116      2.663283      0.940745
H       -0.003504      5.104508      0.374476
H       -0.082941      3.982932     -3.787653
H       -0.049587      5.765963     -2.054928
N       -0.030457     -2.046356     -1.027035
C       -0.047249     -4.721019     -1.775821
C       -0.056879     -2.377562     -2.367944
C       -0.013573     -3.015474     -0.094749
C       -0.021141     -4.365199     -0.427316
C       -0.065220     -3.721706     -2.745427
H        0.006531     -2.671871      0.932814
H       -0.006875     -5.111429      0.359431
H       -0.085981     -3.977570     -3.799425
H       -0.053700     -5.765745     -2.071912
C        0.022302     -0.004287      1.429292
C        0.055115     -0.004185      2.660124
C        0.095355     -0.003012      4.088870
C        0.174226      0.001673      6.881445
C       -1.093955     -0.024594      4.849947
C        1.325786      0.020569      4.781515
C        1.370822      0.023008      6.174018
C       -1.060426     -0.022329      6.242819
H       -2.050836     -0.043123      4.336904
H        2.252019      0.037420      4.214955
H        2.314926      0.041467      6.709411
H       -1.972863     -0.038901      6.830622
F        0.213184      0.004363      8.237414
```

1c

```
40
Molecule 1c
Pt      -0.022111     -0.129306     -0.103121
C       -0.053985     -0.453795     -2.037754
C       -0.097818     -0.901269     -4.761334
C        1.161847     -0.570976     -2.722333
C       -1.291314     -0.552880     -2.685519
C       -1.311503     -0.781615     -4.069828
C        1.137665     -0.798779     -4.106672
H       -2.246293     -0.868001     -4.618318
H        2.054444     -0.897214     -4.682758
N       -2.073236     -0.171322     -0.447245
C       -4.760893     -0.255039     -1.149773
C       -2.427055     -0.388440     -1.764608
C       -3.027649      0.001970      0.483848
C       -4.382580     -0.033558      0.174410
C       -3.776892     -0.432017     -2.119068
H       -2.670271      0.171808      1.492540
H       -5.115638      0.110045      0.960495
H       -4.048014     -0.604552     -3.155043
H       -5.810290     -0.288712     -1.426877
N        2.016951     -0.202222     -0.509210
C        4.680462     -0.341922     -1.289704
C        2.327373     -0.426777     -1.835923
C        3.001289     -0.048970      0.393743
C        4.345523     -0.112487      0.044845
C        3.665117     -0.498715     -2.229754
H        2.676773      0.126821      1.412534
H        5.104019      0.015237      0.809273
H        3.901460     -0.679024     -3.272868
H        5.720455     -0.397918     -1.596959
C        0.014742      0.238268      1.942166
C        0.038428      0.493748      3.146220
C        0.074042      0.822632      4.533081
C       -0.208547     -0.053391      5.562300
H       -0.490158     -1.094781      5.483698
C        0.416803      2.133339      5.039426
H        0.670334      2.965787      4.391814
C        0.387344      2.214679      6.399805
H        0.598940      3.070474      7.027410
S       -0.064466      0.696799      7.119967
H       -0.115295     -1.077924     -5.832907
```

1d

```
49
Molecule 1d
Pt      -0.062311     -0.216986     -1.705448
C       -0.115481     -0.250326     -3.667331
C       -0.188751     -0.301330     -6.425534
C       -1.344680     -0.455729     -4.305547
C        1.077609     -0.068146     -4.377442
C        1.038350     -0.093972     -5.779866
C       -1.379652     -0.482310     -5.708186
H        1.936722      0.043061     -6.376730
H       -2.308105     -0.641002     -6.251236
N        1.946407      0.098455     -2.144896
C        4.567245      0.509308     -2.969653
C        2.238374      0.129189     -3.494386
C        2.927752      0.265253     -1.240519
C        4.251108      0.473531     -1.611419
C        3.555218      0.336500     -3.910369
H        2.617138      0.225828     -0.203141
H        5.008186      0.602674     -0.845891
H        3.778997      0.361648     -4.971431
H        5.590733      0.669891     -3.294768
N       -2.092013     -0.547462     -2.024712
C       -4.754077     -0.976630     -2.694577
C       -2.456276     -0.620366     -3.354976
C       -3.023111     -0.686976     -1.064238
C       -4.364752     -0.902758     -1.357085
C       -3.794075     -0.834908     -3.693148
H       -2.657166     -0.619457     -0.046494
H       -5.079512     -1.008246     -0.548444
H       -4.075273     -0.888959     -4.739357
H       -5.793947     -1.143468     -2.958893
C       -0.005684     -0.187032      0.367036
C        0.033328     -0.171119      1.598002
C        0.082731     -0.162512      3.025531
H        0.088819      1.975466      3.212912
C        0.108756      1.028133      3.745882
C        0.161755     -1.414672      5.123169
C        0.162026      1.039741      5.162242
C        0.109015     -1.398496      3.751196
C        0.190294     -0.205785      5.873421
C        0.189946      2.252868      5.906655
H        0.087480     -2.329402      3.191783
H        0.267821     -1.137263      7.825928
H        0.182571     -2.362134      5.657010
C        0.244658      2.235304      7.283156
H        0.167887      3.196914      5.367085
H        0.266520      3.169295      7.838599
```

C	0.272952	1.002925	7.984415
H	0.315952	1.003109	9.070347
C	0.246222	-0.189509	7.292428
H	-0.217709	-0.322138	-7.511079

1e

```
55
Molecule 1e
Pt      -0.072252     -0.244250     -2.183673
C       -0.124086     -0.269297     -4.145825
C       -0.195042     -0.310242     -6.904290
C       -1.352458     -0.474251     -4.785823
C        1.069150     -0.081849     -4.854274
C        1.031063     -0.102704     -6.256838
C       -1.386193     -0.495904     -6.188582
H        1.929645      0.038410     -6.852418
H       -2.313928     -0.654216     -6.732958
H       -0.223058     -0.327255     -7.989969
N        1.936060      0.080607     -2.620589
C        4.556121      0.502692     -3.442947
C        2.228714      0.116423     -3.969797
C        2.916315      0.249437     -1.715330
C        4.239233      0.462992     -2.085006
C        3.545103      0.328933     -4.384603
H        2.605540      0.207778     -0.678096
H        4.995511      0.593262     -1.318822
H        3.769338      0.357307     -5.445479
H        5.579306      0.667372     -3.767049
N       -2.102354     -0.573182     -2.506104
C       -4.763745     -1.000793     -3.180657
C       -2.465078     -0.642257     -3.837007
C       -3.035101     -0.713918     -1.547322
C       -4.376331     -0.929330     -1.842500
C       -3.802320     -0.856471     -4.177524
H       -2.671420     -0.647364     -0.528751
H       -5.092353     -1.036252     -1.035116
H       -4.082033     -0.908314     -5.224235
H       -5.803212     -1.167401     -3.446790
C       -0.017828     -0.216176     -0.110591
C        0.017975     -0.195772      1.120757
H        0.061759      4.475482      2.624018
C        0.084446      3.550105      3.193343
H        0.020424      2.289634      1.455289
C        0.061198      2.332245      2.539453
C        0.165061      2.403863      5.320808
C        0.088949      1.112986      3.254834
C        0.137365      3.583931      4.598645
C        0.142142      1.140418      4.680827
C        0.063602     -0.162910      2.547123
H        0.156217      4.536679      5.121111
H        0.253435      0.741747      7.424262
H        0.204916      2.460076      6.403320
C        0.087251     -1.328804      3.276431
```

H	0.067002	-2.282792	2.756044
C	0.141109	-1.340279	4.704280
C	0.167121	-2.568720	5.410852
C	0.170613	-0.111226	5.428368
C	0.222684	-2.595569	6.790871
H	0.142954	-3.495341	4.842009
H	0.242863	-3.544392	7.320265
C	0.253346	-1.384381	7.509933
H	0.297516	-1.398598	8.595654
C	0.227744	-0.172168	6.840468

2

```
31
Molecule 2
Pt      -0.000695      0.000000      1.444113
C        0.000463      0.000000     -0.485708
C        0.002760      0.000000     -3.208350
C       -1.229435      0.000000     -1.155087
C        1.231533      0.000000     -1.152363
C        1.234293      0.000000     -2.554442
C       -1.230281      0.000000     -2.557112
H        2.144829      0.000000     -3.145473
H       -2.139866      0.000000     -3.149541
N        2.044324      0.000000      1.098842
C        4.720918      0.000000      0.361897
C        2.383457      0.000000     -0.237952
C        3.006561      0.000000      2.036674
C        4.358237      0.000000      1.708158
C        3.726188      0.000000     -0.613839
H        2.659026      0.000000      3.063405
H        5.101012      0.000000      2.498216
H        3.986412      0.000000     -1.666871
H        5.766959      0.000000      0.071011
N       -2.046105      0.000000      1.094736
C       -4.721872      0.000000      0.353038
C       -2.382963      0.000000     -0.242565
C       -3.009900      0.000000      2.030854
C       -4.361031      0.000000      1.699780
C       -3.725145      0.000000     -0.620618
H       -2.664190      0.000000      3.058175
H       -5.104939      0.000000      2.488816
H       -3.983178      0.000000     -1.674222
H       -5.769136      0.000000      0.059933
Cl      -0.002624      0.000000      3.960553
F        0.004390      0.000000     -4.564060
```

2a

```
43
Molecule 2a
Pt      -0.022623     -0.002862     -0.631900
C       -0.059717      0.000608     -2.592692
C       -0.107295      0.005397     -5.321586
C       -0.071564     -1.224813     -3.269465
C       -0.071163      1.228393     -3.265150
C       -0.095793      1.237178     -4.667631
C       -0.096189     -1.228687     -4.671974
H       -0.106160      2.146840     -5.260433
H       -0.106841     -2.136253     -5.267980
N       -0.029859      2.042332     -1.007412
C       -0.049782      4.719649     -1.749438
C       -0.055396      2.378857     -2.346810
C       -0.012996      3.008853     -0.072890
C       -0.023078      4.359717     -0.402480
C       -0.065410      3.722711     -2.722070
H        0.008755      2.662757      0.953800
H       -0.010199      5.103754      0.386357
H       -0.086134      3.981348     -3.775403
H       -0.058210      5.765091     -2.042613
N       -0.030331     -2.046555     -1.014587
C       -0.051587     -4.721267     -1.765673
C       -0.056183     -2.378477     -2.355131
C       -0.013857     -3.016214     -0.083313
C       -0.024591     -4.365934     -0.417482
C       -0.066857     -3.721045     -2.734938
H        0.008139     -2.673489      0.944506
H       -0.011992     -5.112682      0.368794
H       -0.087860     -3.976033     -3.789148
H       -0.060529     -5.765703     -2.062396
C        0.014651     -0.005453      1.440515
C        0.050071     -0.004895      2.671288
C        0.093705     -0.002818      4.100342
C        0.179614      0.003514      6.923757
C       -1.094512     -0.021148      4.862936
C        1.326158      0.018454      4.789292
C        1.365073      0.021515      6.182438
C       -1.048642     -0.017867      6.255818
H       -2.051203     -0.037705      4.348163
H        2.249735      0.032829      4.217111
H        2.325493      0.038324      6.691670
H       -1.976277     -0.032216      6.822606
H        0.212590      0.006155      8.010019
F       -0.131152      0.007842     -6.678817
```

2b

```
43
Molecule 2b
Pt      -0.052887     -0.003340     -0.639782
C       -0.073045      0.000510     -2.601677
C       -0.101473      0.005798     -5.361446
C       -0.077346     -1.225365     -3.278714
C       -0.076728      1.228949     -3.274008
C       -0.092596      1.229306     -4.677221
C       -0.093207     -1.220335     -4.681912
H       -0.097309      2.155860     -5.246050
H       -0.098372     -2.144679     -5.254327
N       -0.041033      2.042185     -1.014032
C       -0.044076      4.719145     -1.752542
C       -0.059541      2.378133     -2.354222
C       -0.019858      3.007771     -0.077592
C       -0.022343      4.358599     -0.405160
C       -0.061552      3.723877     -2.726216
H       -0.004023      2.658168      0.948201
H       -0.008651      5.102227      0.384086
H       -0.077922      3.984369     -3.779179
H       -0.047413      5.764990     -2.044693
N       -0.041756     -2.047155     -1.021905
C       -0.046731     -4.721265     -1.770219
C       -0.060737     -2.378076     -2.363323
C       -0.021112     -3.016099     -0.088944
C       -0.024552     -4.365701     -0.421489
C       -0.063702     -3.722438     -2.740263
H       -0.004874     -2.670024      0.938063
H       -0.011241     -5.112261      0.364990
H       -0.080464     -3.978982     -3.794184
H       -0.050815     -5.766024     -2.066228
C       -0.000623     -0.006789      1.436339
C        0.044023     -0.006473      2.667068
C        0.092582     -0.003959      4.094971
C        0.186239      0.005632      6.918284
C       -1.093508     -0.023287      4.861083
C        1.326997      0.019499      4.780550
C        1.369809      0.024053      6.173612
C       -1.043864     -0.018309      6.253741
H       -2.051630     -0.042129      4.349106
H        2.249073      0.034305      4.205986
H        2.331628      0.041827      6.680170
H       -1.969988     -0.032077      6.822909
F        0.231748      0.011105      8.300525
F       -0.117128      0.008460     -6.744355
```

2c

```
40
Molecule 2c
Pt      -0.021542     -0.112323     -0.097643
C       -0.053781     -0.447879     -2.030583
C       -0.097656     -0.908662     -4.721416
C        1.161182     -0.571491     -2.714460
C       -1.290560     -0.552507     -2.677360
C       -1.319448     -0.789701     -4.059766
C        1.145256     -0.808059     -4.097149
H       -2.238099     -0.883975     -4.630963
H        2.045052     -0.914999     -4.695402
N       -2.072613     -0.162642     -0.441659
C       -4.760661     -0.251470     -1.145533
C       -2.427441     -0.385448     -1.757739
C       -3.027614      0.010656      0.488405
C       -4.382604     -0.026156      0.177640
C       -3.775824     -0.431592     -2.113988
H       -2.671292      0.183112      1.496989
H       -5.116018      0.119869      0.962932
H       -4.046169     -0.607974     -3.149515
H       -5.809818     -0.286070     -1.423065
N        2.017200     -0.194957     -0.504075
C        4.680710     -0.340842     -1.286649
C        2.328118     -0.425302     -1.829618
C        3.002404     -0.042073      0.397560
C        4.346521     -0.107457      0.046967
C        3.664161     -0.500303     -2.225552
H        2.679325      0.136481      1.416294
H        5.105655      0.022372      0.810391
H        3.899258     -0.684372     -3.268283
H        5.720325     -0.398163     -1.594610
C        0.015562      0.261462      1.943987
C        0.039381      0.509792      3.149382
C        0.074831      0.827895      4.538771
C       -0.202001     -0.057774      5.561309
H       -0.478161     -1.100018      5.474955
C        0.411644      2.136192      5.054880
H        0.660381      2.974862      4.413495
C        0.383647      2.206564      6.415885
H        0.592136      3.058381      7.049886
S       -0.061441      0.681343      7.124555
F       -0.120003     -1.136773     -6.059254
```

2d

```
49
Molecule 2d
Pt      -0.062241     -0.216912     -1.698181
C       -0.115332     -0.250379     -3.660364
C       -0.187433     -0.301276     -6.387500
C       -1.343989     -0.456185     -4.298501
C        1.077341     -0.068000     -4.370223
C        1.046625     -0.092558     -5.772299
C       -1.387078     -0.484445     -5.700303
H        1.927591      0.041665     -6.392667
H       -2.299196     -0.641475     -6.268145
N        1.946004      0.098893     -2.138969
C        4.567163      0.509761     -2.965597
C        2.239056      0.129742     -3.488021
C        2.927674      0.265966     -1.235524
C        4.251085      0.474228     -1.607774
C        3.554395      0.336704     -3.906056
H        2.618022      0.226825     -0.197885
H        5.008380      0.603531     -0.842501
H        3.777502      0.361515     -4.967258
H        5.590432      0.670204     -3.291169
N       -2.091575     -0.547332     -2.019012
C       -4.754080     -0.976217     -2.690843
C       -2.456773     -0.620694     -3.348809
C       -3.023190     -0.686517     -1.059487
C       -4.364972     -0.902149     -1.353737
C       -3.793183     -0.834864     -3.689163
H       -2.658294     -0.618947     -0.041411
H       -5.080077     -1.007322     -0.545371
H       -4.073630     -0.888967     -4.735564
H       -5.793763     -1.142803     -2.955681
C       -0.005685     -0.186642      0.372193
C        0.033310     -0.170631      1.602990
C        0.082742     -0.162216      3.030673
H        0.089206      1.975793      3.218161
C        0.109006      1.028370      3.750969
C        0.161621     -1.414715      5.127981
C        0.162352      1.039777      5.167329
C        0.108823     -1.398382      3.756015
C        0.190417     -0.205903      5.878360
C        0.190526      2.252846      5.911826
H        0.087125     -2.329220      3.196495
H        0.267875     -1.137624      7.830753
H        0.182299     -2.362247      5.661685
C        0.245290      2.235074      7.288313
H        0.168606      3.196950      5.372366
H        0.267345      3.168986      7.843887
```

C	0.273398	1.002566	7.989426
H	0.316440	1.002629	9.075362
C	0.246419	-0.189813	7.297364
F	-0.223580	-0.327590	-7.744133

2e

55
Molecule 2e

Pt	-0.072441	-0.242657	-2.176836
C	-0.124356	-0.267945	-4.139350
C	-0.194264	-0.308969	-6.867044
C	-1.352081	-0.474018	-4.779279
C	1.068377	-0.079992	-4.847698
C	1.038813	-0.099568	-6.250061
C	-1.394022	-0.497335	-6.181360
H	1.920037	0.039038	-6.869082
H	-2.305428	-0.654314	-6.750349
N	1.935377	0.082348	-2.615248
C	4.555578	0.505445	-3.439535
C	2.228993	0.118703	-3.964039
C	2.916037	0.251023	-1.710919
C	4.238915	0.465156	-2.081992
C	3.543792	0.331443	-4.380910
H	2.606317	0.209013	-0.673422
H	4.995460	0.595417	-1.316084
H	3.767264	0.359994	-5.441935
H	5.578436	0.670561	-3.764134
N	-2.101980	-0.573407	-2.500756
C	-4.763624	-1.002133	-3.177044
C	-2.465730	-0.642898	-3.831212
C	-3.034969	-0.715015	-1.542811
C	-4.376265	-0.930861	-1.839294
C	-3.801515	-0.857382	-4.173758
H	-2.672127	-0.648681	-0.523976
H	-5.092462	-1.038218	-1.032137
H	-4.080633	-0.909102	-5.220632
H	-5.802858	-1.168872	-3.443627
C	-0.017683	-0.216636	-0.105991
C	0.018554	-0.198013	1.125203
H	0.067038	4.473448	2.625485
C	0.088579	3.548384	3.195343
H	0.023899	2.286748	1.458016
C	0.064327	2.330268	2.542072
C	0.167301	2.403537	5.323843
C	0.090630	1.111619	3.258429
C	0.141016	3.583042	4.600868
C	0.143375	1.139839	4.684567
C	0.064279	-0.164849	2.551685
H	0.160597	4.536133	5.122631
H	0.252799	0.742768	7.428359
H	0.206832	2.460310	6.406273
C	0.086819	-1.330407	3.281679
H	0.065912	-2.284751	2.762004

C	0.140198	-1.341035	4.709810
C	0.164892	-2.569004	5.417442
C	0.170399	-0.111505	5.433109
C	0.219722	-2.594976	6.797598
H	0.140288	-3.496016	4.849292
H	0.238849	-3.543429	7.327631
C	0.250949	-1.383364	7.515894
H	0.294457	-1.396812	8.601610
C	0.226709	-0.171651	6.845433
F	-0.229378	-0.330424	-8.223759

Optimized geometries (triplet state)

1

```
31
Molecule 1 (triplet)
Pt       0.002326      0.003181     -1.423569
C       -0.014046     -0.000948      0.480449
C        0.030392     -0.007680      3.234366
C        1.281994     -0.002579      1.152452
C       -1.241894     -0.002620      1.153221
C       -1.238005     -0.006016      2.548834
C        1.245476     -0.006037      2.590014
H       -2.153343     -0.007346      3.131321
H        2.164494     -0.007518      3.168034
H        0.015614     -0.010434      4.320910
N       -2.063336      0.002539     -1.093450
C       -4.735836      0.000971     -0.356131
C       -2.399048     -0.000531      0.241765
C       -3.022541      0.004747     -2.031642
C       -4.375697      0.004076     -1.701715
C       -3.739269     -0.001370      0.620644
H       -2.674458      0.007054     -3.058309
H       -5.119423      0.005970     -2.490872
H       -3.998631     -0.003839      1.673755
H       -5.781493      0.000368     -0.063768
N        2.027689      0.002466     -1.112612
C        4.735165      0.000858     -0.326812
C        2.377029     -0.000669      0.278914
C        3.001299      0.004723     -2.031589
C        4.353025      0.004073     -1.698811
C        3.759675     -0.001466      0.637424
H        2.668735      0.007066     -3.064160
H        5.093108      0.005997     -2.490992
H        4.020324     -0.003940      1.691274
H        5.784690      0.000262     -0.050928
Cl      -0.004016      0.008643     -3.928015
```

1a

```
43
Molecule 1a (triplet)
Pt      -0.097784     -0.020649     -0.634395
C       -0.065349     -0.005991     -2.569973
C       -0.016767      0.000068     -5.319749
C       -0.186791     -1.263020     -3.255339
C        0.083655      1.227860     -3.242278
C        0.109406      1.239734     -4.631258
C       -0.163046     -1.222430     -4.666972
H        0.223038      2.155735     -5.203451
H       -0.256619     -2.129491     -5.258803
H        0.002114      0.010711     -6.406250
N        0.131654      2.033721     -0.985815
C        0.439771      4.701323     -1.724107
C        0.192648      2.373258     -2.323806
C        0.222159      2.996586     -0.049616
C        0.376809      4.338913     -0.378309
C        0.346380      3.705997     -2.698133
H        0.168527      2.657976      0.978204
H        0.444899      5.077201      0.413360
H        0.392676      3.959899     -3.751749
H        0.560208      5.740310     -2.014339
N       -0.306392     -2.036159     -0.994282
C       -0.572538     -4.726347     -1.782258
C       -0.317428     -2.382571     -2.372155
C       -0.429285     -3.023170     -0.072353
C       -0.561837     -4.354022     -0.410145
C       -0.450956     -3.735776     -2.737820
H       -0.415052     -2.695398      0.960825
H       -0.655571     -5.094608      0.376897
H       -0.456614     -3.987397     -3.794707
H       -0.673542     -5.766891     -2.073530
C       -0.057649     -0.026550      1.397114
C        0.011835     -0.018910      2.639521
C        0.087632      0.000426      4.049737
C        0.245983      0.041677      6.860476
C       -0.004485     -1.200318      4.798954
C        0.260138      1.222500      4.748519
C        0.338077      1.237107      6.135878
C        0.074737     -1.174075      6.186232
H       -0.137842     -2.140383      4.272000
H        0.331925      2.146043      4.182032
H        0.471296      2.180309      6.658512
H        0.002568     -2.101060      6.748276
H        0.307414      0.057861      7.945052
```

1b

```
43
Molecule 1b (triplet)
Pt      -0.042644     -0.006073     -0.639677
C       -0.063293      0.000462     -2.576234
C       -0.090243     -0.001245     -5.325873
C       -0.204378     -1.256261     -3.253190
C        0.063182      1.233278     -3.256951
C        0.050593      1.239196     -4.646132
C       -0.215709     -1.222490     -4.662354
H        0.145498      2.153575     -5.224502
H       -0.321267     -2.132421     -5.247869
H       -0.100528      0.004259     -6.412481
N        0.174565      2.045244     -1.005391
C        0.456032      4.712801     -1.762416
C        0.196013      2.381708     -2.346637
C        0.291944      3.013258     -0.075947
C        0.434001      4.354064     -0.413701
C        0.335959      3.713492     -2.729255
H        0.268942      2.679400      0.954511
H        0.524280      5.094995      0.373287
H        0.350870      3.963817     -3.784701
H        0.565137      5.750996     -2.059745
N       -0.263784     -2.022718     -0.987438
C       -0.562422     -4.715198     -1.756440
C       -0.314656     -2.375021     -2.361255
C       -0.363681     -3.007138     -0.057827
C       -0.510804     -4.337842     -0.387260
C       -0.463609     -3.726680     -2.718607
H       -0.319399     -2.675437      0.973217
H       -0.584284     -5.075576      0.404781
H       -0.500630     -3.981153     -3.774213
H       -0.677790     -5.755965     -2.041279
C       -0.001146     -0.008481      1.388879
C        0.039702     -0.004198      2.633599
C        0.090193     -0.003433      4.042287
C        0.198317     -0.003251      6.824754
C       -0.013086     -1.215859      4.773764
C        0.248953      1.209038      4.763416
C        0.303715      1.212471      6.149882
C        0.040686     -1.218952      6.160292
H       -0.135438     -2.149295      4.233783
H        0.329348      2.142393      4.215526
H        0.426606      2.132006      6.712679
H       -0.037292     -2.138429      6.731055
F        0.251545     -0.003339      8.171663
```

1c

```
40
Molecule 1c (triplet)
Pt      -0.032609     -0.026371     -0.116384
C       -0.053621     -0.457421     -2.011498
C       -0.084350     -1.050177     -4.698908
C        1.182657     -0.539787     -2.702291
C       -1.305645     -0.668887     -2.645070
C       -1.308250     -0.968190     -4.010580
C        1.155420     -0.842021     -4.066098
H       -2.233630     -1.137491     -4.555269
H        2.067879     -0.920015     -4.651678
N       -2.074982     -0.213941     -0.424641
C       -4.778607     -0.523484     -1.112880
C       -2.437621     -0.530813     -1.739594
C       -3.053336     -0.058539      0.502592
C       -4.395147     -0.202484      0.203900
C       -3.785037     -0.684045     -2.073103
H       -2.713438      0.188382      1.501429
H       -5.130947     -0.065951      0.989503
H       -4.043911     -0.931952     -3.097920
H       -5.824824     -0.643279     -1.374939
N        2.001596     -0.002443     -0.518702
C        4.687892     -0.028757     -1.327384
C        2.335104     -0.282080     -1.848249
C        2.998396      0.253875      0.364861
C        4.333588      0.250598      0.005855
C        3.674115     -0.293331     -2.243047
H        2.679580      0.464261      1.378866
H        5.085654      0.463767      0.758254
H        3.911596     -0.513362     -3.279290
H        5.727850     -0.038232     -1.637342
C       -0.017138      0.419875      1.846852
C       -0.017510      0.692164      3.068502
C       -0.018423      0.992185      4.431373
C       -1.183393      1.104811      5.202921
H       -2.202670      0.980488      4.862867
C        1.166865      1.225671      5.233427
H        2.170003      1.185223      4.824778
C        0.884382      1.496524      6.533091
H        1.569401      1.707527      7.343742
S       -0.840484      1.480426      6.842186
H       -0.096407     -1.282726     -5.760129
```

1d

49			
Molecule 1d (triplet)			
Pt	-0.190933	-0.162221	-1.687274
C	-0.120049	-0.283896	-3.642690
C	-0.018145	-0.465823	-6.394841
C	-1.248076	-0.755805	-4.331184
C	1.057920	0.097890	-4.301906
C	1.105946	0.004633	-5.700503
C	-1.192654	-0.846911	-5.729666
H	1.995048	0.287633	-6.258398
H	-2.039264	-1.206077	-6.309338
N	1.743533	0.518222	-2.041854
C	4.287218	1.391233	-2.762483
C	2.104192	0.551624	-3.377944
C	2.626954	0.907243	-1.101882
C	3.904123	1.348828	-1.418840
C	3.380454	0.990258	-3.738930
H	2.274791	0.851997	-0.078652
H	4.577964	1.651182	-0.624719
H	3.653915	1.013441	-4.788521
H	5.278743	1.731783	-3.044839
N	-2.096627	-0.888270	-2.091367
C	-4.584309	-1.861154	-2.873822
C	-2.360148	-1.094264	-3.434492
C	-3.046665	-1.157007	-1.174721
C	-4.299438	-1.642889	-1.522979
C	-3.608520	-1.583453	-3.826406
H	-2.767415	-0.968079	-0.144889
H	-5.029662	-1.844179	-0.746915
H	-3.806685	-1.742684	-4.881118
H	-5.553951	-2.241282	-3.180595
C	-0.263639	-0.047066	0.344787
C	-0.312905	0.010578	1.598804
C	-0.342539	0.080491	2.972883
H	1.711723	0.870604	3.158467
C	0.832802	0.569441	3.720899
C	-1.492501	-0.232231	5.103777
C	0.834606	0.650946	5.118445
C	-1.486594	-0.309233	3.737419
C	-0.342707	0.247315	5.854957
C	1.962353	1.121356	5.853731
H	-2.362430	-0.672421	3.206701
H	-1.227803	0.024141	7.790964
H	-2.374335	-0.534552	5.663262
C	1.933948	1.192034	7.249194
H	2.851008	1.426625	5.306896
H	2.806504	1.554198	7.786515

C	0.794544	0.800668	7.948520
H	0.768046	0.853816	9.033020
C	-0.340575	0.328649	7.240410
H	0.022233	-0.537335	-7.477881

1e

```
55
Molecule 1e (triplet)
Pt      -0.096382     -0.169540     -2.178196
C       -0.187690     -0.279455     -4.133976
C       -0.325717     -0.421361     -6.887260
C       -1.281063     -0.930489     -4.723431
C        0.838542      0.302175     -4.892764
C        0.765064      0.228372     -6.291623
C       -1.348578     -1.000882     -6.122749
H        1.534699      0.663220     -6.924388
H       -2.176127     -1.493030     -6.627805
H       -0.379815     -0.476768     -7.970663
N        1.661987      0.817912     -2.698684
C        3.943337      2.106372     -3.637627
C        1.879475      0.919515     -4.061179
C        2.559579      1.336280     -1.838664
C        3.709410      1.987127     -2.265593
C        3.023493      1.568150     -4.532435
H        2.327444      1.211082     -0.788006
H        4.400886      2.388087     -1.532662
H        3.185930      1.644993     -5.602286
H        4.832142      2.609988     -4.005473
N       -1.885440     -1.207914     -2.417824
C       -4.232023     -2.589889     -2.987909
C       -2.231866     -1.454000     -3.734077
C       -2.687238     -1.634696     -1.423772
C       -3.866289     -2.327569     -1.665395
C       -3.409244     -2.148923     -4.020077
H       -2.351255     -1.400856     -0.420425
H       -4.478051     -2.650696     -0.830317
H       -3.671898     -2.339983     -5.055096
H       -5.146591     -3.130336     -3.212446
C       -0.026020     -0.049033     -0.140049
C       -0.008374      0.020396      1.114234
H        3.242394      3.341501      2.834512
C        2.568343      2.665472      3.354024
H        1.751577      1.843574      1.542122
C        1.734756      1.822362      2.627656
C        1.651643      1.766542      5.400777
C        0.845616      0.931263      3.266100
C        2.529149      2.638368      4.749605
C        0.799994      0.904362      4.695067
C       -0.019727      0.058851      2.487645
H        3.172240      3.290974      5.333546
H        0.422246      0.514944      7.407418
H        1.639624      1.770984      6.485432
C       -0.946557     -0.822494      3.214719
```

H	-1.598012	-1.465733	2.631007
C	-0.999248	-0.861772	4.603655
C	-1.905684	-1.743796	5.279964
C	-0.131484	-0.014498	5.394872
C	-1.957567	-1.798328	6.659605
H	-2.553944	-2.374714	4.676688
H	-2.650455	-2.475151	7.152668
C	-1.110453	-0.976777	7.422076
H	-1.141673	-1.012373	8.507053
C	-0.215101	-0.101812	6.782407

2

```
31
Molecule 2 (triplet)
Pt      -0.000001      0.002520     -1.414895
C        0.000004     -0.000424      0.482658
C       -0.000010     -0.005301      3.189263
C        1.265433     -0.001589      1.146416
C       -1.265439     -0.001589      1.146418
C       -1.258358     -0.004129      2.563547
C        1.258353     -0.004128      2.563538
H       -2.153711     -0.005314      3.172954
H        2.153704     -0.005314      3.172949
N       -2.055599      0.001851     -1.107876
C       -4.731313      0.000510     -0.358126
C       -2.386567     -0.000250      0.245849
C       -3.015290      0.003155     -2.040749
C       -4.369325      0.002551     -1.713958
C       -3.745551     -0.000873      0.616392
H       -2.671300      0.004826     -3.068921
H       -5.111732      0.003673     -2.503639
H       -4.007740     -0.002467      1.669043
H       -5.778255     -0.000001     -0.069642
N        2.055606      0.001851     -1.107875
C        4.731313      0.000510     -0.358133
C        2.386573     -0.000250      0.245841
C        3.015294      0.003155     -2.040751
C        4.369330      0.002551     -1.713959
C        3.745547     -0.000873      0.616388
H        2.671302      0.004826     -3.068922
H        5.111737      0.003673     -2.503640
H        4.007737     -0.002467      1.669038
H        5.778255     -0.000001     -0.069645
Cl       0.000004      0.007176     -3.889265
F       -0.000002     -0.007856      4.529700
```

2a

```
43
Molecule 2a (triplet)
Pt      -0.013223      0.000886     -0.634672
C       -0.068658      0.010438     -2.568051
C       -0.167716     -0.011071     -5.285772
C       -0.226650     -1.263647     -3.237443
C        0.040561      1.233920     -3.249667
C       -0.006662      1.250642     -4.643946
C       -0.275666     -1.237158     -4.660081
H        0.069822      2.144112     -5.252801
H       -0.396588     -2.128653     -5.266803
N        0.202979      2.060554     -1.003666
C        0.494047      4.712299     -1.770494
C        0.198762      2.385104     -2.342177
C        0.353440      3.020409     -0.079629
C        0.502432      4.363106     -0.423259
C        0.341466      3.714142     -2.734790
H        0.349924      2.686063      0.951100
H        0.621295      5.105521      0.358067
H        0.335873      3.963629     -3.790176
H        0.605855      5.748562     -2.074547
N       -0.237145     -2.018666     -0.968049
C       -0.514983     -4.708578     -1.734035
C       -0.310511     -2.363745     -2.348321
C       -0.297154     -2.995069     -0.041040
C       -0.433948     -4.333831     -0.363951
C       -0.453353     -3.730853     -2.700125
H       -0.232376     -2.661373      0.988534
H       -0.475756     -5.072039      0.429164
H       -0.510261     -3.990106     -3.753301
H       -0.623243     -5.752173     -2.012844
C        0.034585     -0.000981      1.408921
C        0.055094      0.004587      2.647731
C        0.082227     -0.003040      4.066304
C        0.137002     -0.021396      6.881697
C       -0.042328     -1.216184      4.785040
C        0.234231      1.200571      4.795143
C        0.261643      1.186709      6.185789
C       -0.014887     -1.220385      6.175726
H       -0.160988     -2.144798      4.234413
H        0.332291      2.136358      4.252656
H        0.381576      2.118680      6.731658
H       -0.113631     -2.159537      6.713432
H        0.158920     -0.028769      7.968021
F       -0.218300      0.015762     -6.633753
```

2b

```
43
Molecule 2b (triplet)
Pt      -0.021314    -0.004304    -0.640837
C       -0.058754     0.009547    -2.574222
C       -0.119879    -0.005155    -5.293299
C       -0.031626    -1.268796    -3.252311
C       -0.114793     1.241066    -3.248094
C       -0.147759     1.261201    -4.642539
C       -0.064137    -1.239019    -4.674626
H       -0.194042     2.161140    -5.244863
H       -0.047776    -2.133995    -5.288082
N       -0.098047     2.066208    -0.996025
C       -0.191589     4.737908    -1.745366
C       -0.133115     2.396662    -2.333096
C       -0.108617     3.031228    -0.065140
C       -0.155298     4.383681    -0.399883
C       -0.180482     3.734978    -2.716774
H       -0.078801     2.692473     0.963726
H       -0.162179     5.129725     0.387111
H       -0.208060     3.988083    -3.771040
H       -0.228060     5.781521    -2.042630
N        0.039535    -2.032732    -0.987064
C        0.110487    -4.731647    -1.767231
C        0.021936    -2.376525    -2.369087
C        0.090657    -3.014725    -0.064536
C        0.127421    -4.357830    -0.394585
C        0.058673    -3.748140    -2.728263
H        0.101389    -2.680652     0.966926
H        0.167681    -5.100270     0.394657
H        0.044935    -4.007147    -3.783039
H        0.138232    -5.778807    -2.052347
C        0.019738    -0.007763     1.402731
C        0.051022    -0.004483     2.641516
C        0.089853    -0.007807     4.058249
C        0.171677    -0.017031     6.842674
C        0.065963     1.205244     4.788641
C        0.155232    -1.225730     4.778073
C        0.196513    -1.234763     6.166940
C        0.106905     1.205055     6.177578
H        0.015128     2.146211     4.249990
H        0.174166    -2.162945     4.230960
H        0.247953    -2.162118     6.728050
H        0.089230     2.128587     6.746994
F        0.212324    -0.021378     8.192142
F       -0.152322     0.023242    -6.641980
```

2c

```
40
Molecule 2c (triplet)
Pt      -0.033190     -0.011366     -0.123096
C       -0.061327     -0.450553     -2.009602
C       -0.074247     -1.048947     -4.662557
C        1.206395     -0.533480     -2.695368
C       -1.296684     -0.665218     -2.641947
C       -1.330480     -0.972570     -4.003456
C        1.164298     -0.848319     -4.081707
H       -2.236764     -1.148563     -4.571403
H        2.053641     -0.937007     -4.697261
N       -2.106027     -0.203884     -0.431490
C       -4.779940     -0.518529     -1.115543
C       -2.444070     -0.522966     -1.728048
C       -3.065758     -0.044959      0.491731
C       -4.418221     -0.194030      0.188864
C       -3.782941     -0.683690     -2.078960
H       -2.724208      0.207065      1.488749
H       -5.159010     -0.055927      0.968631
H       -4.038943     -0.937111     -3.101811
H       -5.824043     -0.642709     -1.385936
N        1.990101      0.013934     -0.502552
C        4.680285     -0.020475     -1.317332
C        2.322284     -0.277655     -1.855703
C        2.980912      0.271379      0.377620
C        4.318457      0.267260      0.028954
C        3.688166     -0.286524     -2.233629
H        2.657657      0.485757      1.390149
H        5.066991      0.482761      0.783294
H        3.935903     -0.510057     -3.267303
H        5.723823     -0.030098     -1.616500
C       -0.018156      0.434384      1.863694
C       -0.022095      0.693783      3.077165
C       -0.013470      0.986229      4.455905
C       -1.160639      1.096010      5.234082
H       -2.185488      0.978753      4.908500
C        1.181372      1.208033      5.242207
H        2.179241      1.166551      4.820396
C        0.918249      1.470396      6.550560
H        1.615979      1.671754      7.352667
S       -0.796995      1.458482      6.877324
F       -0.111063     -1.347895     -5.979289
```

2d

```
49
Molecule 2d (triplet)
Pt      -0.206789     -0.145673     -1.678518
C       -0.135694     -0.258136     -3.634094
C       -0.032613     -0.429340     -6.355554
C       -1.259717     -0.737563     -4.324303
C        1.039468      0.135558     -4.291813
C        1.096119      0.049645     -5.689900
C       -1.210749     -0.826879     -5.722532
H        1.968230      0.333466     -6.271024
H       -2.037064     -1.187279     -6.327584
N        1.721119      0.551838     -2.031441
C        4.257460      1.452598     -2.751629
C        2.082639      0.594870     -3.367657
C        2.602393      0.945635     -1.091110
C        3.874828      1.400691     -1.407954
C        3.353441      1.046605     -3.728882
H        2.252253      0.883120     -0.067646
H        4.546130      1.706241     -0.612894
H        3.625485      1.076611     -4.778718
H        5.245297      1.803932     -3.033224
N       -2.105774     -0.885457     -2.085994
C       -4.585572     -1.879408     -2.874407
C       -2.369202     -1.089515     -3.430098
C       -3.054397     -1.167024     -1.171387
C       -4.302205     -1.663361     -1.522702
C       -3.611743     -1.588536     -3.825407
H       -2.778151     -0.980005     -0.140453
H       -5.030527     -1.874597     -0.747482
H       -3.807690     -1.745462     -4.880924
H       -5.551335     -2.267580     -3.182897
C       -0.276090     -0.043214      0.349834
C       -0.319630      0.004784      1.604904
C       -0.337977      0.065053      2.978788
H        1.714227      0.857959      3.151630
C        0.840946      0.549687      3.718971
C       -1.469532     -0.268395      5.116310
C        0.855513      0.618058      5.117148
C       -1.476040     -0.333722      3.750294
C       -0.313851      0.205846      5.860425
C        1.990087      1.082924      5.845675
H       -2.355512     -0.693839      3.223699
H       -1.180493     -0.036757      7.803229
H       -2.345624     -0.577006      5.681352
C        1.974967      1.139857      7.240701
H        2.872830      1.394480      5.292889
H        2.851911      1.497686      7.773661
```

C	0.841529	0.740095	7.947753
H	0.826075	0.783126	9.032898
C	-0.298779	0.274085	7.247436
F	0.019801	-0.515702	-7.709367

2e

```
55
Molecule 2e (triplet)
Pt      -0.109735     -0.146720     -2.172162
C       -0.189605     -0.275005     -4.127422
C       -0.308399     -0.444155     -6.849007
C       -1.273759     -0.941239     -4.716651
C        0.836406      0.306970     -4.885984
C        0.779129      0.222730     -6.284496
C       -1.339165     -1.030695     -6.114397
H        1.533044      0.647941     -6.940007
H       -2.146020     -1.527494     -6.644694
N        1.640592      0.854119     -2.693779
C        3.917950      2.150976     -3.634769
C        1.867710      0.942142     -4.055770
C        2.527444      1.392332     -1.835012
C        3.674380      2.047886     -2.263329
C        3.008571      1.593332     -4.528846
H        2.289396      1.279604     -0.784334
H        4.356884      2.464826     -1.530931
H        3.177628      1.657505     -5.598511
H        4.805147      2.657042     -4.002841
N       -1.888478     -1.201831     -2.413145
C       -4.227045     -2.599111     -2.986056
C       -2.227521     -1.462147     -3.728732
C       -2.695079     -1.623197     -1.420791
C       -3.869897     -2.323022     -1.664158
C       -3.399163     -2.164210     -4.017107
H       -2.367033     -1.379353     -0.417248
H       -4.485539     -2.640796     -0.829876
H       -3.654674     -2.364887     -5.052100
H       -5.138237     -3.144747     -3.211302
C       -0.044340     -0.018542     -0.136975
C       -0.022870      0.045757      1.117323
H        3.268803      3.318811      2.854014
C        2.591712      2.643301      3.370132
H        1.750795      1.854426      1.554592
C        1.743367      1.818743      2.639827
C        1.681563      1.728111      5.412851
C        0.849934      0.928985      3.274061
C        2.562917      2.598812      4.765729
C        0.815780      0.883307      4.702837
C       -0.031376      0.076847      2.491256
H        3.217287      3.237306      5.352710
H        0.468348      0.448783      7.412969
H        1.677339      1.719624      6.497516
C       -0.970501     -0.795626      3.214475
H       -1.641407     -1.415526      2.627650
```

C	-1.009042	-0.855816	4.603468
C	-1.923452	-1.733094	5.275371
C	-0.118934	-0.035390	5.398133
C	-1.960052	-1.810324	6.654351
H	-2.589677	-2.341978	4.669129
H	-2.658783	-2.483258	7.144377
C	-1.089349	-1.016996	7.420265
H	-1.107795	-1.071463	8.504748
C	-0.187085	-0.145729	6.785182
F	-0.368113	-0.527868	-8.202533
